# Chiroptical Enhancement of Chiral Dicarboxylic Acids from Confinement in a Stereodynamic Supramolecular Cage

**DOI:** 10.1021/acssensors.2c00038

**Published:** 2022-04-26

**Authors:** Federico Begato, Roberto Penasa, Giulia Licini, Cristiano Zonta

**Affiliations:** Department of Chemical Sciences, University of Padova, via Marzolo 1, 35131 Padova, Italy

**Keywords:** chirality, host−guest chemistry, molecular recognition, self-assembly, supramolecular cages, supramolecular chemistry

## Abstract

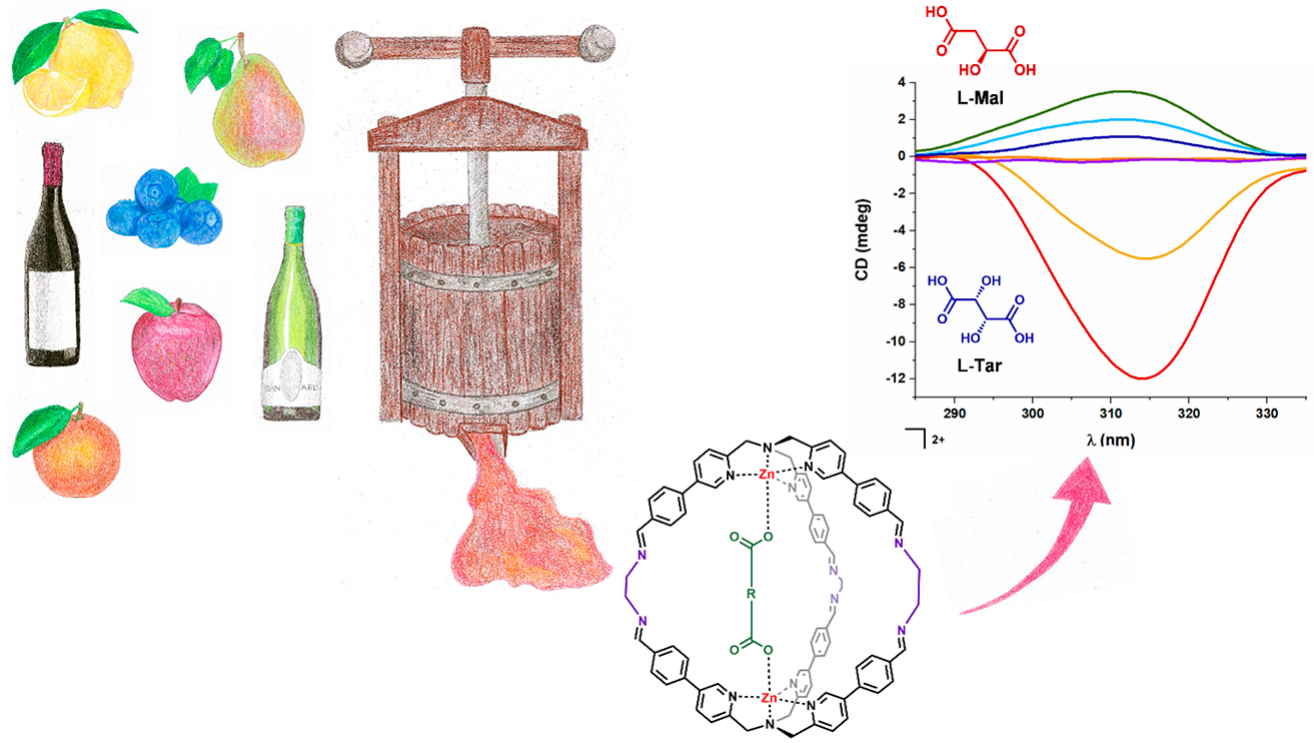

The fundamental implications that chirality has in science and technology require continuous efforts for the development of fast, economic, and reliable quantitative methods for enantiopurity assessment. Among the different analytical approaches, chiroptical techniques in combination with supramolecular methodologies have shown promising results in terms of both costs and time analysis. In this article, a tris(2-pyridylmethyl)amines (**TPMA**)-based supramolecular cage is able to amplify the circular dichroism (CD) signal of a series of chiral dicarboxylic acids also in the presence of a complex mixture. This feature has been used to quantify tartaric acid in wines and to discriminate different matrixes using principal component analysis (PCA) of the raw CD data.

Since Pasteur’s tartrate experiment highlighting the significance of “dissymmetry”, control of chirality at the molecular level has led to many technological and scientific advancements in physics, chemistry, and life sciences.^1^ Along with the progress of this area, the quantification of enantiomeric excess (e.e.) has urged the development of fast and effective methods. Within this context, promising results have been reported by the use of supramolecular approaches which have developed molecular sensors able to amplify chiroptical signal intensities.^2−11^ The leading strategy in this field is represented by the use of chemosensors carrying a chromophore unit and a labile stereogenic element in fast racemization.^12−17^ Interaction of these stereodynamic probes with a chiral analyte shifts the equilibrium among the two enantiomeric forms of the receptor toward a preferential diastereoisomer. The presence of chromophores allows to translate this bias into a signal which is detected using electronic circular dichroism (CD). Among the different molecular architectures exploiting this feature, metal complexes of tris(2-pyridylmethyl)amine (**TPMA**)^18^ ligands have gained considerable attention due to the seminal contributions of Zahn and Canary et al.,^19,20^ Anslynet al.,^21,22^ and, more recently, by our group.^23,24^ These complexes exploit the propeller-like arrangement of the ligand around the metal center, whose configuration is controlled by the interaction with the chiral analyte. However, it should be noted that while these probes have shown a good capability to amplify CD signals of a wide variety of molecular systems, one unresolved issue remains—their application in the presence of other possible interfering analytes. Indeed, while the versatility toward different functional groups can be considered an analytical strength, low specificity in the presence of complex mixtures or reaction crudes, just to cite some practical examples, can represent a hampering weakness. In particular, the main drawback comes in those cases in which the presence of other chiral components within the analytical mixture can interfere with the chiroptical output.

We recently reported the use of **TPMA**-based supramolecular cages able to self-assemble in the presence of a complex mixture like wine or fruit juices.^25^ In these mixtures, cages were able to selectively encapsulate dicarboxylic acids present in the matrixes. Herein, we report the chiroptical analytical employment of a **TPMA** cage, which highlighted that a confined stereodynamic structure can allow the e.e. determination of chiral dicarboxylic acids also in complex mixtures. The reported system displayed a preferential enhancement of the dichroic signal for tartaric acid which is more than 1 order of magnitude higher than the structural closest system malic acid.

## Results and Discussion

In recent years, we have been interested in carboxylic acids sensing^26−29^ using **TPMA**-based supramolecular cages.^30−32^ The high affinity and selectivity of our system toward diacids, together with the capability to form in complex mixtures, prompted us to investigate if it was possible to take advantage also of the stereodynamic features of the two **TPMA** units in chiral sensing.^33 −35^ For these reasons, we investigated the recognition capabilities and chiroptical properties of the molecular cage **1** toward: l-malic acid (**l**-**Mal**), l-tartaric acid (l-**Tar**), the amino acids *Boc*-l-glutamic acid (**l**-**Glu**), *Boc*-l-aspartic acid (**l**-**Asp**), and (1*R*,3*S*)-camphoric acid (***R*,S**-**Cam**).

**Figure 1:**
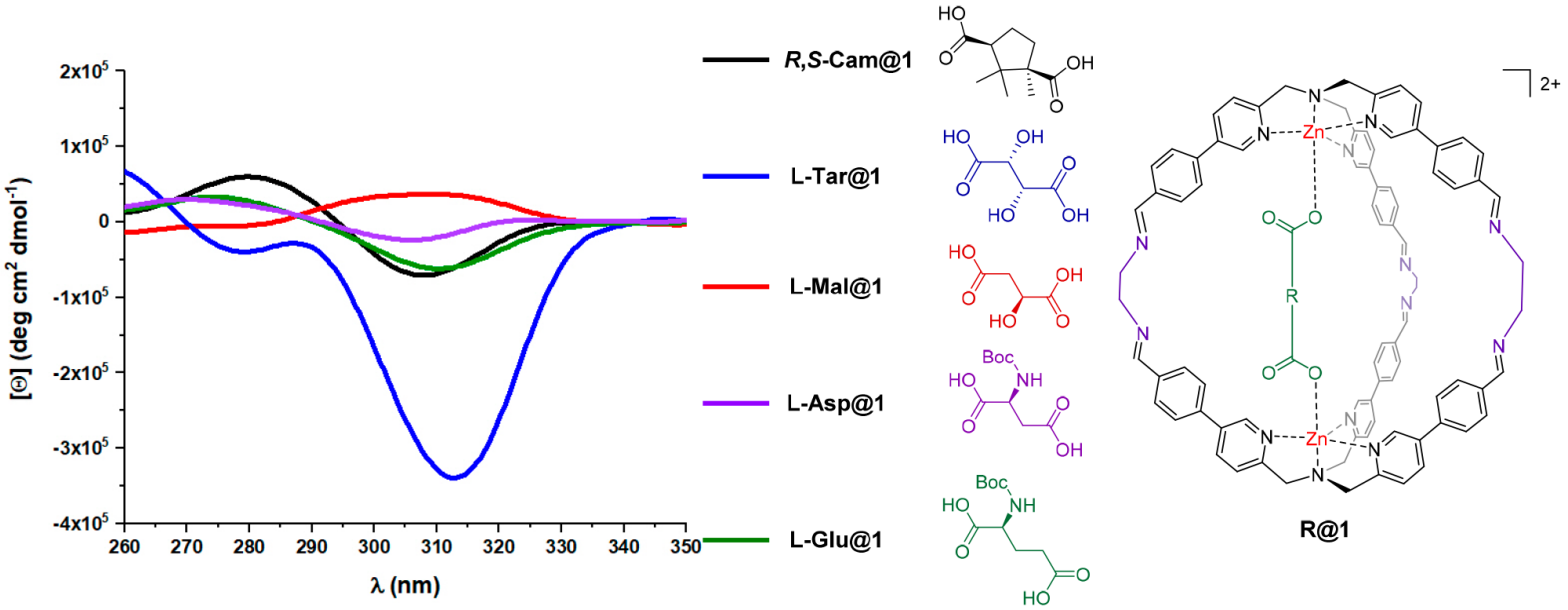
Circular dichroism spectra for the **R@1** series. Solution of molecular cages containing the different diacids have been analyzed using CD spectroscopy. Dichroic signals are observed for all diacid. Among them, **l**-**Tar** acid is furnishing the stronger signal. CD measurements were performed by diluting the synthesized cage with anhydrous DMSO to obtain a final concentration equal to 1.0 × 10^–5^ M (0.1 cm cuvette). The counterions are perchlorate for the cage.

The enclosed cages were formed taking advantage of the imine-based dynamic covalent chemistry process obtained by mixing the aldehyde precursor with ethylenediamine in the presence of the chiral diacid in DMSO-*d*_6_. After 12 h, the formation of the cages was confirmed for all the systems by ^1^H NMR, 2D-NMR (COSY, DOSY), and ESI-MS analyses (Figures S18–S25). Once cage systems were formed, dichroic signals were observed for all five differently included cages in the spectral region between 260 and 350 nm, a region where the free diacids do not display any meaningful signal. Additional investigations revealed a linearity in the CD intensity response as a function of the e.e. of the guest (Figure S2). Unexpectedly, while for four embedded diacids the intensities are in line with previously reported **TPMA** probes,^23^ a higher signal enhancement was observed in the case of encapsulated tartaric acid **l**-**Tar@1** (Figure 1). This feature was also more remarkable considering that the closely related system incorporating malic acid **l**-**Mal@1**, which displayed a binding constant similar to **l**-**Tar@1** (Table S6), had a signal intensity 1 order of magnitude lower (**l**-**Tar@1** [θ] = −3.5 and **l**-**Mal@1** [θ] = 0.36 deg cm^2^ dmol^–1^ 10^5^ at 314 nm).

**Figure 2:**
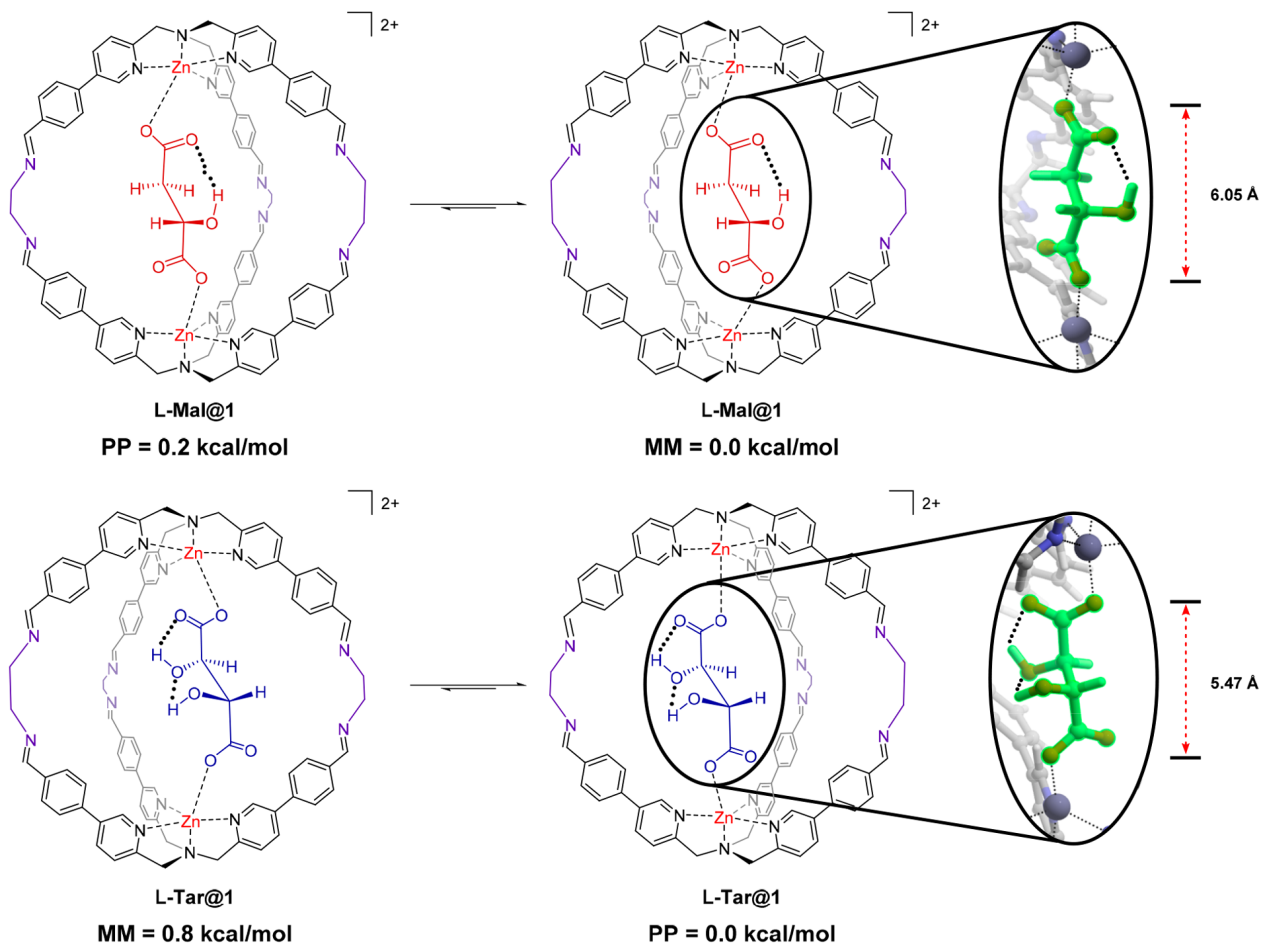
Diacids lead mainly to the formation of two diastereomeric conformations characterized by the opposite helicity of the **TPMA** unit (*MM* or *PP*) according to DFT calculations. Energy difference among the diastereomeric structures is 0.2 kcal/mol for the **l**-**Mal** and 0.8 kcal/mol for the **l**-**Tar** acids. The higher energy difference calculated in the latter case is ascribable to the formation of two intramolecular hydrogen bonds, which results in a tightening of the cage (representative distances in **l**-**Mal@1** O–O 6.05 Å and Zn–Zn 9.67 Å, l-Tar@1 O–O 5.47 Å and Zn–Zn 9.11 Å).

To clarify the origin of the signal enhancement in the case of **l**-**Tar**, TD-DFT calculations on the **l**-**Mal@1** and **l**-**Tar@1** cages were carried out (Section S4). In more detail, initially a conformational search was performed to identify the structures responsible for the observed signals. Possible conformations are ruled by the propeller-like arrangement of the ligand around the metal and the conformations of the enclosed diacids. The latter were essentially dictated by the intramolecular network of hydrogen bonds among hydroxyls and carboxylates (Figure 2 and Section S4.1). The lowest energy structures found for the two inclusion systems highlighted intramolecular hydrogen bonds within the diacids, two in the case of **l**-**Tar** versus one in the case of **l**-**Mal**. These hydrogen bonds are responsible for a shorter length of the guest in the case of **l**-**Tar@1** in comparison with l-**Mal@1**. This influences the size of the cage and in its capability to adopt the two enantiomeric forms. The extra hydrogen bond in **l**-**Tar** induces a tightening in **l**-**Tar@1**, which corresponds to a higher thermodynamic differentiation among the two diastereomeric forms of the cage in comparison to **l**-**Mal@1**. This difference in population is responsible for signal intensities as confirmed by the overlap between the calculated and the experimental CD spectra (Figures S10–S11).

As mentioned in the introduction, we already reported the capability of this system to form also in the presence of complex matrixes, such as fruit juices and wines, taking advantage of the templating capability of dicarboxylic acids present in these solutions.^25^ Due to the “natural” chiral character of these templates, the chiroptical probe was tested using complex mixtures as sources of the diacids. In the first experiment, the capability of the cage to preferentially enhance the **l**-**Tar** signal was exploited to quantify the tartaric acid content of the wines using circular dichroism. In more detail, the standard addition method was used to minimize the effect of the sample matrix.

In particular, cage synthesis has been optimized in the presence of wine, reducing the time of formation to 20 min (Figure S28), and they have been assembled using the different wines and increasing aliquots of commercially available optically pure **l**-**Tar** (Figures S12–S17).

The **l**-**Tar** content obtained by the CD investigation has been compared with the **l**-**Tar** content measured using ^1^H NMR with an internal standard (Table 1).^25^

**Table 1:** **l**-**Tar** Acid Content in Different Wines Obtained with Standard Addition Method and ^1^H-NMR Peak Integration in the Presence of an Internal Standard

	tartaric acid content	
wine	CD(g/L)	NMR(g/L)^a^
Prosecco	1.1	1.3
Chianti	2.3	2.4
Chardonnay	1.7	1.5
Barbera	2.5	2.5
Müller-Thurghau	1.2	1.5
Valpolicella	2.2	2.0

a Values have been taken from ref (21).

**Figure 3:**
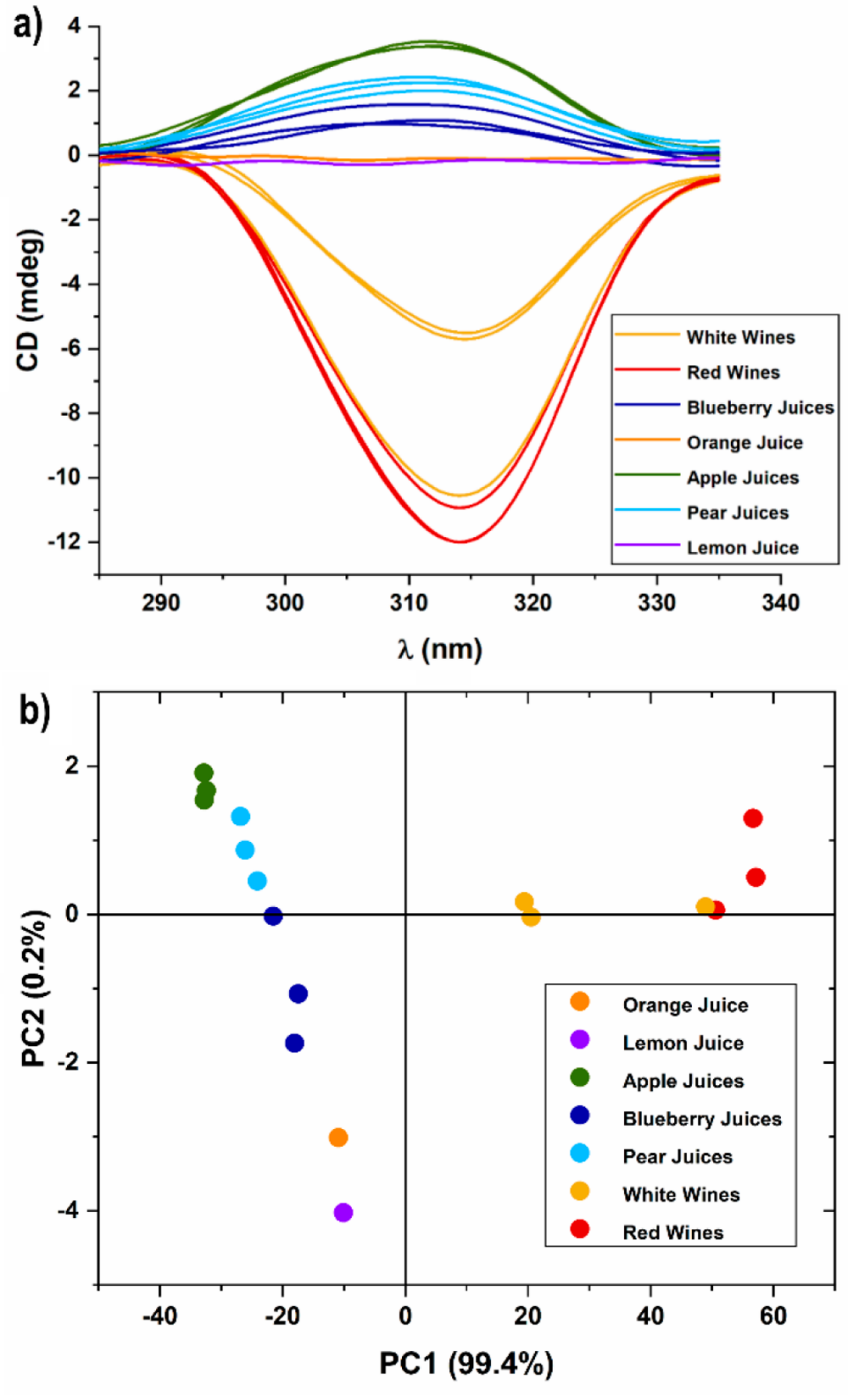
(a) CD spectra of the supramolecular cage 1 formed upon addition of 15 μL of different fruit juices and wines without pretreatment to a DMSO-*d*_6_ solution containing 500 μL of the aldehyde zinc complex and 125 μL of ethylenediamine and (b) PCA analysis. ^1^H NMR of all the formed cages present in the PCA and relative **l**-**Tar** and **l**-**Mal** values are reported in Section S10.

In a second experiment, cage synthesis was performed using 11 different juices and 6 wines as the source of templating agent. CD of the resulting mixtures were registered and the collected data analyzed using the Principal Component Analysis (PCA) method (Figure 3).^36,37^ Even though the CD spectra seem mainly dictated by the **l**-**Tar** (*viz*. negative curves) and **l**-**Mal** (*viz*. positive curves) contents, PCA showed an effective degree of separation allowing discrimination among the different “templating” matrixes. PC1, which accounted for more than 99% of the total variation, showed a direct correlation with the **l**-**Tar** acid content.

Wines with high l-Tar content are in the positive region of PC1, while two white wines are present in an intermediate region of PC1 axis. In this case, as shown by ^1^H NMR analysis, **l**-**Tar** content is low. All the other juices display a negative PC1, and discrimination is obtained along PC2. High **l**-**Mal** content systems (pears and apples) are in the positive PC2 region, while systems that do not present high contents of either **l**-**Mal** or **l**-**Tar** are in the negative PC2 region. It is also interesting to notice that PC1 and PC2 loadings strongly resembled CD spectra of **l**-**Tar** and **l**-**Mal**, respectively (Figures S29–S47).

It should be highlighted that even if a naked eye impression over the CD spectra in Figure 3 seemed uninformative, unexpectedly, the differences in CD spectra of the two natural diacids, (e.g., absolute value, intensity, and maximum absorbance wavelength) were sufficient to furnish a distinct discrimination among the different natural matrixes.

## Conclusions

In conclusion, we reported a supramolecular cage able: (i) to act as sensor for chiral diacids, (ii) to display a CD signal 1 order of magnitude higher for **l**-**Tar** in comparison with the structurally related **l**-**Mal**, (iii) to report **l**-**Tar** content in wines, and (iv) to discriminate different juices using PCA. These results have been obtained combining the stereodynamic properties of the two **TPMA** units together with the properties arising from cage confinement. It should be stressed that dynamic covalent chemistry has already been successfully exploited in complex mixtures taking advantage of differential sensing in dynamic chemical networks.^38 −40^ However, the possibility to master the self-assembly of a defined molecular architecture in the presence of a complex mixture and to report a signal urges novel opportunities in the preparation of innovative functional supramolecular systems in more challenging matrices.

## Supporting Information Available

Experimental details and characterization of all new compounds; selected 2D-NMR experiments (COSY, DOSY) and the detailed conformational search among the structures (DFT, TD-DFT calculations) (PDF)
