# Supplementary material for: Chiroptical Enhancement of Chiral Dicarboxylic Acids from Confinement in a Stereodynamic Supramolecular Cage
Source: ACS Sens. 2022 Apr 26;7(5):1390–4. doi: 10.1021/acssensors.2c00038 (PMC9150167; doi:10.1021/acssensors.2c00038)
Supplement: Supplementary file 1 — se2c00038_si_001.pdf [file se2c00038_si_001.pdf]

Supporting Information for

Chiroptical Enhancement of Chiral Dicarboxylic Acids from Confinement in a Stereodynamic Supramolecular Cage

Authors: Federico Begato,[1] Roberto Penasa,[1] Giulia Licini,[1] and Cristiano Zonta[1]*

[1] Department of Chemical Sciences, University of Padova, via Marzolo 1, 35131 Padova

*Correspondence to: cristiano.zonta@unipd.it

Contents

Supporting Information for 1
1 General Methods 4
2 Synthesis and Characterization 5
2.1 Synthesis of Cages R@1 5
2.2 Synthesis of Cages in the presence of complex mixtures 5
2.2.1 L-Tar@1 6
2.2.2 L-Mal@1 6
2.2.3 *R*,*S*-Cam@1 6
2.2.4 L-Glu@1 6
2.2.5 L-Asp@1 6
3 CD measurements 7
3.1 CD spectra of Cages R@1 7
3.2 Enantiomeric Excess curve for Tar@1 8
4 Computational section 9
4.1 Conformational Analysis 9
4.1.1 Dicarboxylic Acid Coordination 11
4.1.2 Meso Structures 12
4.1.3 Energy calculation of the most stable conformers 13
4.2 B3LYP/6-31G(d) Best minimized structures 15
4.3 TD-DFT Calculations 17
5 Calibration Curves for the different wines 19
5.1 Prosecco 19
5.2 Chianti 20
5.3 Chardonnay 20
5.4 Valpolicella 21

5.5 Müller-Thurgau 21
5.6 Barbera 22
6 NMR and ESI-MS characterization 23
6.1 L-Tar@1 23
6.2 L-Mal@1 24
6.3 *R*,*S*-Cam@1 25
6.4 L-Glu@1 25
6.5 L-Asp@1 26
6.6 ESI-MS spectrum L-Tar@1 27
7 Binding constant determination 28
7.1 Titration Experiment of L-Tartaric Acid with Molecular Cage 1 29
7.2 Titration Experiment of L-Malic Acid with Molecular Cage 1 30
8 Kinetic Experiment of Cage Formation Using Wine as Template 31
9 Procedure for CD Data analysis with PCA 32
9.1 Circular Dichroism Spectra 34
9.1.1 Prosecco@1 34
9.1.2 Chardonnay@1 34
9.1.3 Chianti@1 35
9.1.4 Valpolicella@1 35
9.1.5 Müller-Thurgau@1 36
9.1.6 Barbera@1 36
9.1.7 Blueberry juice-1@1 37
9.1.8 Blueberry juice-2@1 37
9.1.9 Blueberry juice-3@1 38
9.1.10 Apple juice-1@1 38
9.1.11 Apple juice-2@1 39
9.1.12 Apple juice-3@1 39
9.1.13 Pear juice-1@1 40
9.1.14 Pear juice-2@1 40
9.1.15 Pear juice-3@1 41
9.1.16 Orange Juice@1 41
9.1.17 Lemon Juice@1 42
10 Quantification of Dicarboxylic acids Content in the Complex Mixtures for the PCA Analysis 43
10.1 Prosecco@1 44
10.2 Chianti@1 44
10.3 Chardonnay@1 45
10.4 Valpolicella@1 45
10.5 Müller-Thurgau@1 46

10.6 Barbera@1 46
10.7 Blueberry juice-1@1 47
10.8 Blueberry juice-2@1 47
10.9 Blueberry juice-3@1 48
10.10 Apple Juice-1@1 48
10.11 Apple Juice-2@1 49
10.12 Apple Juice-3@1 49
10.13 Pear Juice-1@1 50
10.14 Pear Juice-2@1 50
10.15 Pear Juice-3@1 51
10.16 Lemon Juice@1 51
10.17 Orange Juice@1 52
11 Coordinates of optimized best structures 53
11.1 Tar@1 entry 10 53
11.2 Tar@1 entry 17 57
11.3 Tar@1 entry 9 61
11.4 Mal@1 entry 15 65
11.5 Mal@1 entry 16 69
11.6 Mal@1 entry 11 73
12 References 77

1 General Methods

NMR spectra were recorded at 301 K on Bruker 400 Avance III BBi-z grad 5 mm and Bruker Avance-III 500MHz instruments. All the ^{1}H-NMR spectra were referenced to residual isotopic impurity of DMSO-d_6 (2.50 ppm). The following abbreviations are used in reporting the multiplicity for NMR resonances; s=single, d=doublet, t= triplet, and m=multiplet. The NMR data were processed using Bruker Topspin 3.5 pl2 and MestReNova 12.0.0.
High resolution electrospray ionization mass spectrometry HRMS (ESI-TOF) analyses were performed in positive mode with Waters Xevo G2-S QTof. The analysis was performed with Fast Flow Injection: 10 µL of sample injected ACN at 30 ul/min. Capillary: 3000V, Sample cone: 30V, Source temperature: 80 °C, Desolvation temperature: 250 °C. ECD spectra were recorded with a Jasco J-1500 spectrometer and processed with Spectra Manager Version 2.15.3.1 or OriginPro 2018 (64-bit) SR1 b9.5.1.195.
The computational searches of the most stable structures and the TD-DFT calculations were carried out Gaussian 16 package[1] Revision C.01 and processed with GaussView 6.0.16[2] or CYL view BETA 1.0. The experimental/theoretical comparison of the CD spectra were carried out with SpecDis Version 1.71[3]. Chemicals were purchased from Merck, TCI, or Apollo Scientific and used without further purification.

2 Synthesis and Characterization

2.1 Synthesis of Cages R@1

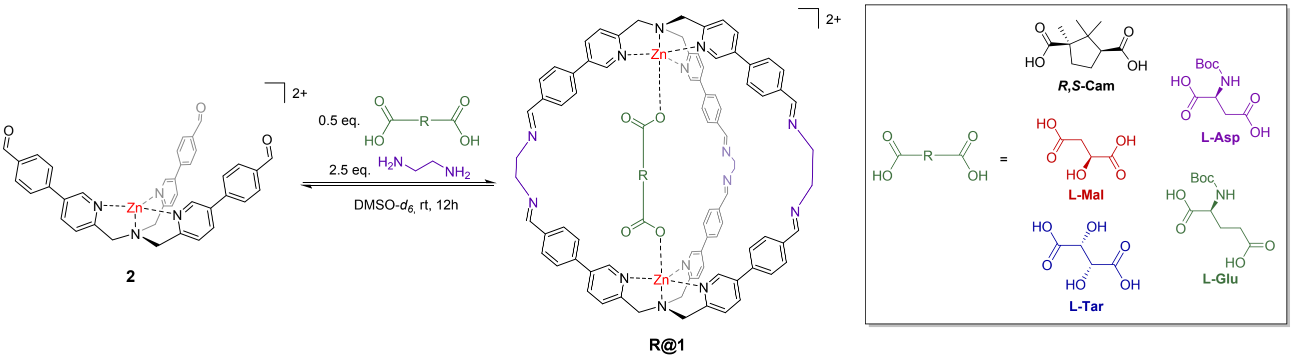

General procedure for the synthesis of molecular cages **R@1**. Perchlorate counterions are removed for clarity.

To 500 µL (1.0 µmol) of a solution 0.002 M of the aldehyde zinc complex **2** in DMSO-d_6, 50 µL (0.5 µmol) of a solution 0.01 M in DMSO-d_6 of a dicarboxylic acid **R** and 125 µL (2.5 µmol) of a solution 0.02 M in DMSO-d_6 of ethylenediamine were added in a NMR tube. The mixture was left overnight at room temperature and checked *via* ^{1}H-NMR and ESI-MS. The yield for all the cages is >90% (Determined *via* ^{1}H-NMR on internal standard *p*-xylene).

2.2 Synthesis of Cages in the presence of complex mixtures

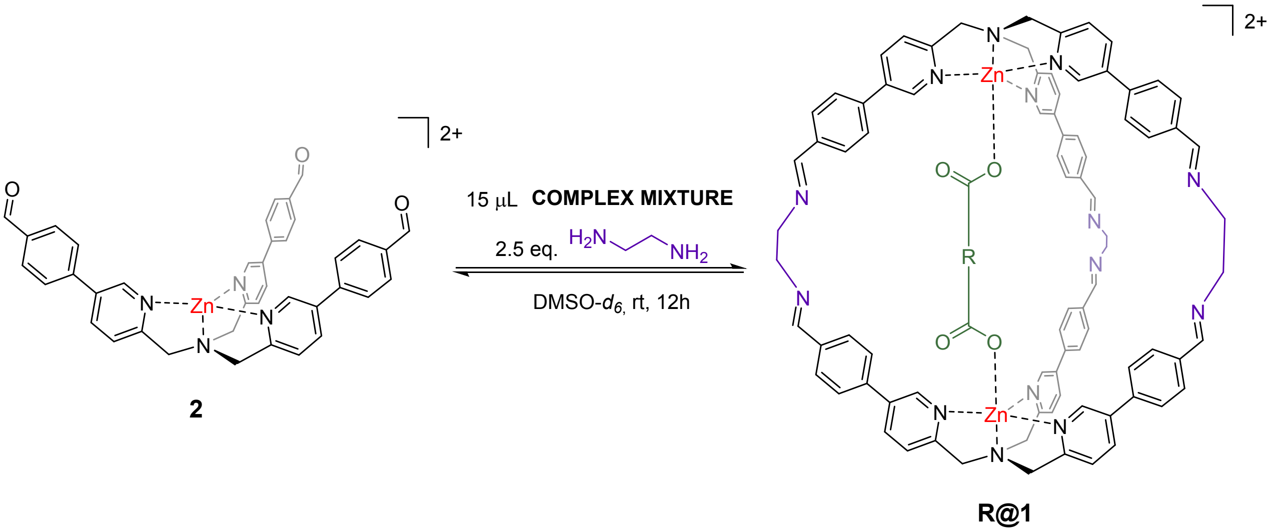

General procedure for the synthesis of molecular cages **R@1**. Perchlorate counterions are removed for clarity.

To 500 µL (1.0 µmol) of a solution 0.002 M the aldehyde zinc complex **2** in DMSO-d_6, 15 µL of complex mixture (wine or fruit juice) without pre-treatment and 125 µL (2.5 µmol) of a solution 0.02 M in DMSO-d_6 of ethylenediamine were added in a NMR tube. The mixture was left overnight compl at room temperature and checked *via* ^{1}H-NMR.

2.2.1 L-Tar@1

^{1}H-NMR (500 MHz, DMSO-d_6) δ (ppm): 9.37 (s, 6H, PyrH), 8.23 (s, 6H, NH_{imm}), 8.16 (d, J = 7.9 Hz, 6H, PyrH), 7.85 (d, J = 8.0 Hz, 12H, ArH), 7.70 (d, J = 7.4 Hz, 12H, ArH), 7.49 (s, 6H, PyrH), 4.98 (s, 2H, OH_{TAR}), 4.83 (d, J = 4.8 Hz, 2H, CH_{TAR}), 4.64 – 4.21 (m, 12H, $CH_{2\text{-}TPMA}$), 3.98 (d, J = 7.5 Hz, 6H, $CH_{2\text{-}EDA}$), 3.86 (d, J = 7.5 Hz, 6H, $CH_{2\text{-}EDA}$).
MS (ESI-MS) (*m/z*): [M^{2+}] calcd. for $[C_{88}H_{76}N_{14}O_6Zn_2]^{2+}$, 776.2328; found 776.2319

2.2.2 L-Mal@1

^{1}H-NMR (500 MHz, DMSO-d_6) δ (ppm): 9.39 (s, 3H, PyrH), 9.30 (s, 3H, PyrH), 8.28 (s, 3H, NH_{imm}), 8.26 (s, 3H, NH_{imm}), 8.18 (d, 8.8 Hz, 3H, PyrH), 8.13 (d, 8.8 Hz, 3H, PyrH), 7.85 (d, J = 7.8 Hz, 12H, ArH), 7.66 – 7.63 (m, 12H, ArH), 7.52 (d, 8.2 Hz, 3H, PyrH), 7.48 (d, 8.2 Hz, 3H, PyrH), 4.92 – 4.90 (m, 2H, OH_{MAL} – CH_{MAL}), 4.45 – 4.42 (m, 12H, $CH_{2\text{-}TPMA}$), 3.95 – 3.91 (m, 12H, $CH_{2\text{-}EDA}$), 2.84 – 2.82 (m, 2H, $CH_{2\text{-}MAL}$).
MS (ESI-MS) (*m/z*): [M^{2+}] calcd. for $[C_{88}H_{76}N_{14}O_5Zn_2]^{2+}$, 768.2347; found 768.2348

2.2.3 *R,S*-Cam@1

^{1}H-NMR (500 MHz, DMSO-d_6) δ (ppm): 9.26 (s, 3H, PyrH), 9.23 (s, 3H, PyrH), 8.51 – 8.31 (m, 12H, NH_{imm} – PyrH), 7.90 – 7.84 (m, 12H, ArH), 7.80 – 7.69 (m, 12H, ArH), 7.65 – 7.62 (m, 6H, PyrH), 4.39 (b, m, 12H, $CH_{2\ TPMA}$), 3.91 (b, m, 12H, $CH_{2\text{-}EDA}$), 2.85 (m, 2H, CH_{CAM}), 2.37 (m, 1H, CH_{CAM}), 1.99 (m, 1H, CH_{CAM}), 1.76 (m, 1H, CH_{CAM}), 1.60 – 1.53(m, 6H, CH_{CAM}), 1.23 – 1.13 (m, 3H, CH_{CAM}).
MS (ESI-MS) (*m/z*): [M^{2+}] calcd. for $[C_{94}H_{86}N_{14}O_4Zn_2]^{2+}$, 801.2764; found 801.2739

2.2.4 L-Glu@1

^{1}H-NMR (500 MHz, DMSO-d_6) δ (ppm): 9.31 (s, 3H, PyrH), 9.18 (s, 3H, PyrH), 8.23 – 8.21 (m, 12H, NH_{imm} – PyrH),), 7.80 – 7.75 (m, 12H, ArH), 7.72 – 7.60 (m, 12H, ArH), 7.57 – 7.54 (m, 6H, PyrH), 7.14 – 7.06 (b, m, 1H, NH_{GLU}), 4.32 – 4.24 (m, 3H, $CH_{2\text{-}EDA}$ – CH_{GLU}), 3.83 (b, m, 12H, $CH_{2\text{-}EDA}$), 1.27 (b, m, 2H, CH_{GLU}), 1.15 (b, m, 1H, CH_{GLU}), 1.08 (b, m, 1H, CH_{GLU}), 0.84 (b, m, 1H, CH_{GLU}), 0.75 (s, 9H, $CH_{3\text{-}GLU}$).
MS (ESI-MS) (*m/z*): [M^{2+}] calcd. for $[C_{94}H_{87}N_{15}O_6Zn_2]^{2+}$, 824.7768; found 824.7758

2.2.5 L-Asp@1

^{1}H-NMR (500 MHz, DMSO-d_6) δ (ppm): 9.34 – 9.31 (m, 6H, PyrH), 8.30 – 8.27 (m, 6H, NH_{imm}), 8.18 – 8.17 (m, 6H, PyrH), 7.84 – 7.82 (m,12H, ArH), 7.73 – 7.63 (m, 12H, ArH), 7.53 – 7.51 (m, 6H, PyrH), 7.30 – 7.28 (b, m, 1H, NH_{ASP}), 4.68 (b, m, 1H, CH_{ASP}), 4.44 (s, 12H, $CH_{2\text{-}TPMA}$), 3.94 (s, 12H, $CH_{2\text{-}EDA}$), 2.74 (s, 1H, CH_{ASP}), 1.24 (s, 1H, CH_{ASP}), 0.89 – 0.85 (b, m, CH_{ASP}), 0.62 (s, 9H, CH_{ASP}).
MS (ESI-MS) (*m/z*): [M^{2+}] calcd. for $[C_{93}H_{85}N_{15}O_6Zn_2]^{2+}$, 817.7690; found 817.7681

3 CD measurements

CD measurements were performed diluting with anhydrous DMSO the synthesized cage to obtain a final concentration equal to $1.0 \cdot 10^{-5}$M (0.1 cm cuvette). The CD spectra were measured in millidegrees, normalized for the concentration of the cage and reported as [Θ], following the formula:

$$[\Theta] = \frac{\Theta}{C \cdot l}$$

Where [Θ] is the molar ellipticity, Θ is the CD value registered from the instrument (expressed in mdeg), *C* is the concentration of the sample, expressed in (mol/L) and *l* is the optical path (expressed in cm).

3.1 CD spectra of Cages R@1

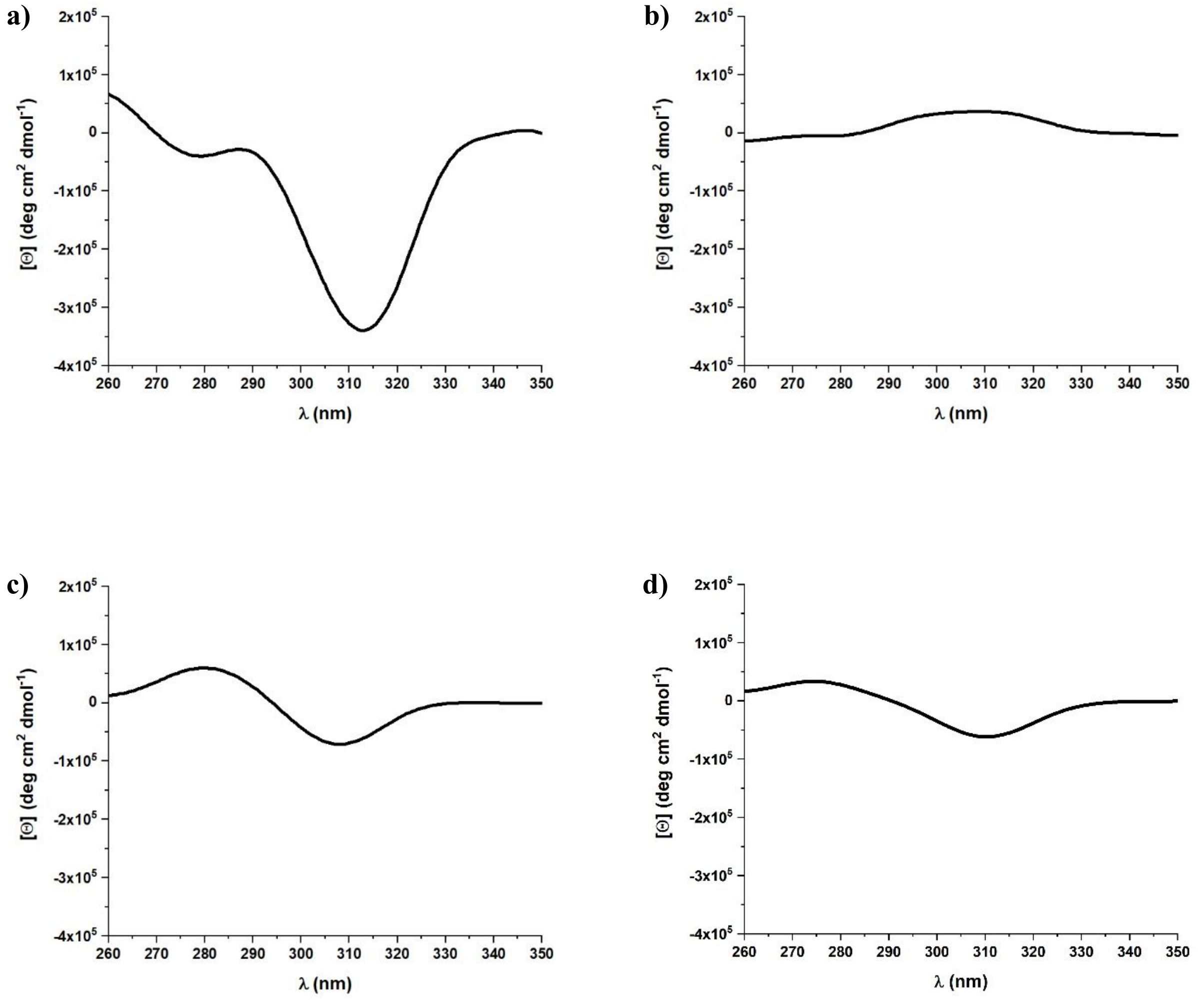

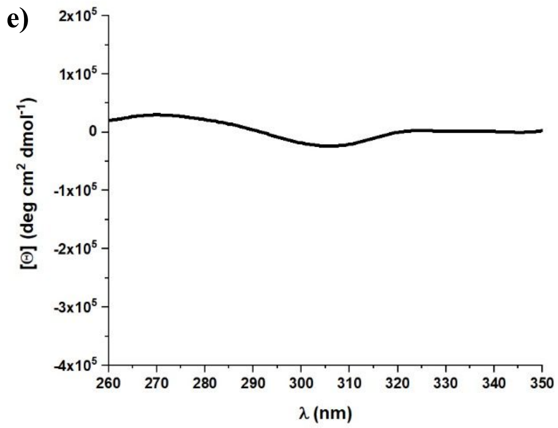

Figure S1 Molar ellipticity spectra of cage **R@1**, a) L-**Tar@1**, b) L-**Mal@1**, c) ***R*,*S*-Cam@1**, d) L-**Glu@1**, e) L-**Asp@1**. The CD spectra were recorded in anhydrous DMSO at 25°C ($1.0\cdot10^{-5}$ M, l=0.1 cm).

3.2 Enantiomeric Excess curve for Tar@1

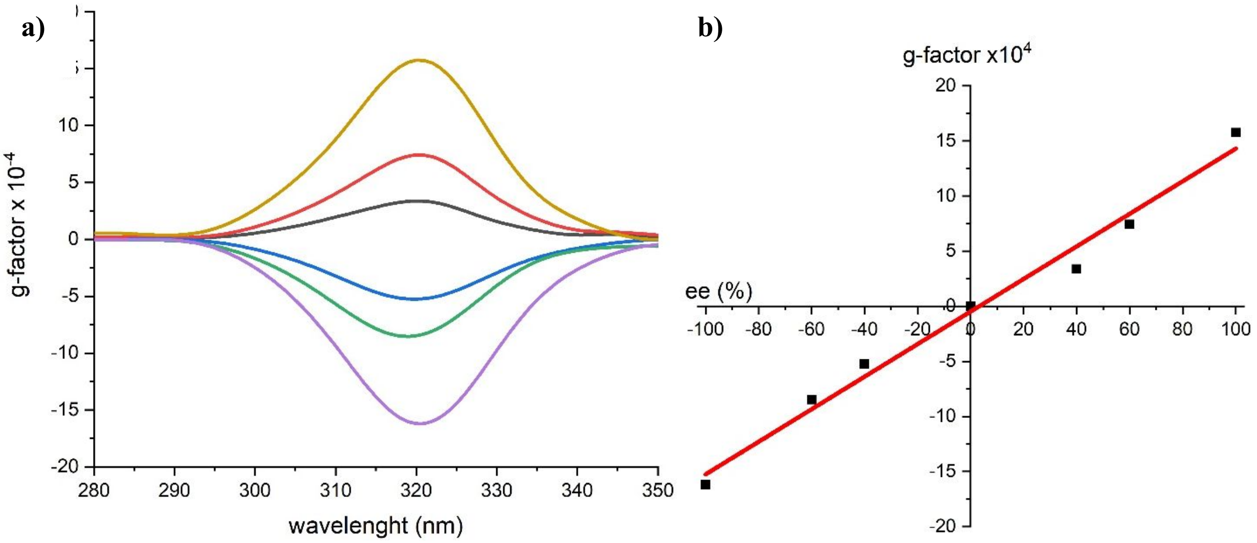

Figure S2 a) Anisotropy g-factor for the six samples of D-tartaric acid analyzed, respectively with an ee (%) equal to +100, +60, +40, -40, -60 and -100; b) calibration curve obtained with the g-factor of the samples at 320 nm. The fitting equation curve is y=0.1478(±0.0083)x-0.48(±0.55), R^2=0.9814.

4 Computational section

4.1 Conformational Analysis

Manual conformational search was run with Gaussian 16 package (DFT B3LYP/6-31G(d)), with default grids and loose convergence criteria[4]. The guests chosen for the computational investigation are the D-Tartaric acid and L-malic acid. Three stereogenic elements have been varied to generate eight different starting structures for the conformational search. In particular: *i*) the TPMA helix helicity sense (Figure S3), *ii*) the dihedral angle between the pyridine ring and the imine ring (Figure S4), *iii*) the dihedral angle of the imine bond relative to the aromatic ring (Figure S5).

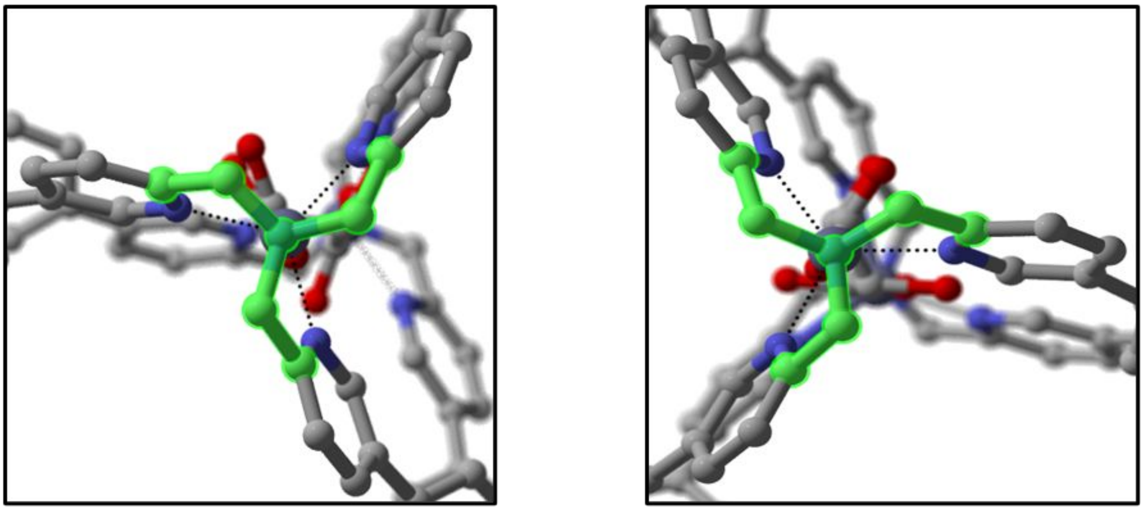

Figure S3 Helix conformation clockwise **Δ** (left) and counterclockwise **Λ** (right) of **TPMA**. Hydrogen atoms are removed for clarity.

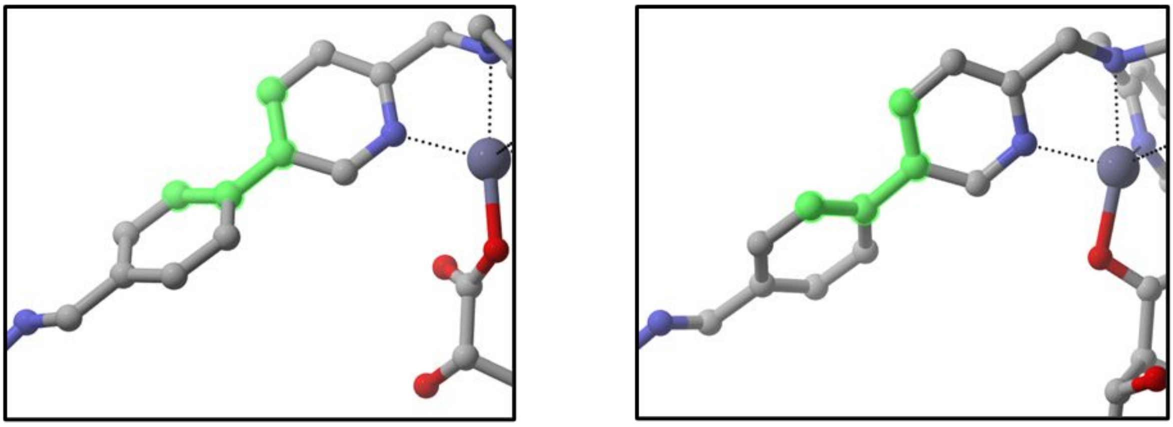

Figure S4 Dihedral angle formed between the pyridine-ring of **TPMA** and the phenyl-ring: example of positive angle on left (+) and negative angle on right (-). Hydrogen atoms are removed for clarity.

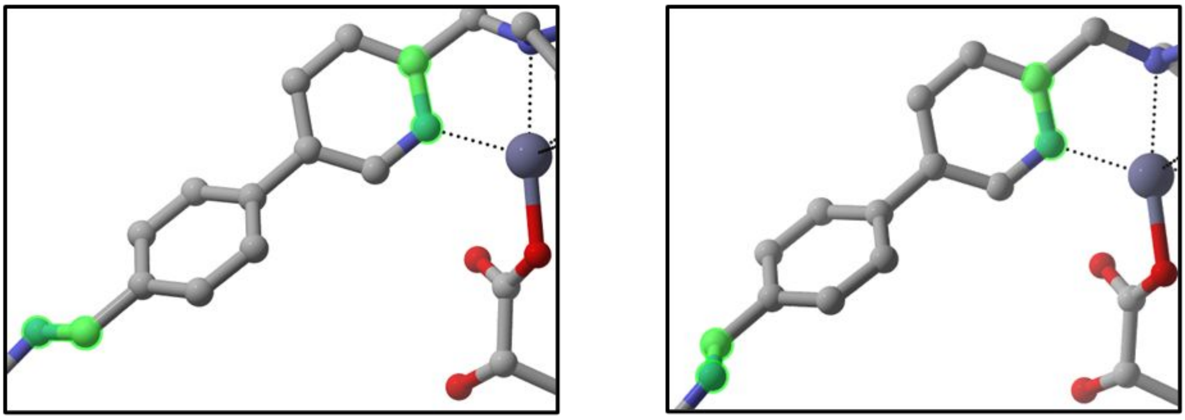

Figure S5. Orientation of the C=N bond of the imine group respect the C-N highlighted on the **TPMA** pyridine ring: C=N forms an angle >|**90°**| (left); C=N forms an angle <|**90°**| (right). Hydrogen atoms are removed for clarity.

Conformers	Helix	Dihedral Angle Pyr-Ar	Dihedral Angle Pry-Imine
**I**	Λ	-	<|90°|
**II**	Δ	-	<|90°|
**III**	Λ	+	<|90°|
**IV**	Λ	+	>|90°|
**V**	Δ	+	<|90°|
**VI**	Δ	+	>|90°|
**VII**	Λ	-	>|90°|
**VIII**	Δ	-	>|90°|

Table S1. Classification of conformations assumed by cages stereoisomers: the propeller direction of **TPMA**, **Λ** (counterclockwise), **Δ** (clockwise); the sign of dihedral angle formed between the pyridine-ring of **TPMA** and the phenyl-ring (**+** for a positive angle, **-** for a negative angle); the orientation of the C=N bond of the imine group respect the C-N bond on the **TPMA** pyridine ring (angle <|**90°**| or >|**90°**|).

As shown in Table S3, relative energies for the eight different conformers clearly indicate a preference of the cage to adopt mainly two conformations. These two conformations, namely **I** and **V** are the lowest in energy for both the diacid under investigation. These two conformations were subsequently used to vary the conformations of the diacid within the cage.

4.1.1 Dicarboxylic Acid Coordination

The search for the more stable cage conformation was then followed by a conformational analysis over the dicarboxylic acids, trying to expand the number minima explored on the potential energy surface. Due to the large energy difference of the two conformations **I** and **V** among the others, this search was done only with these two conformations of the cage. In Figure S6 and Figure S7 the four possible conformers for D-Tartaric acid and five for L-Malic acid are shown. The changes are mainly driven by the different network of hydrogen bonds formed within the molecule itself.

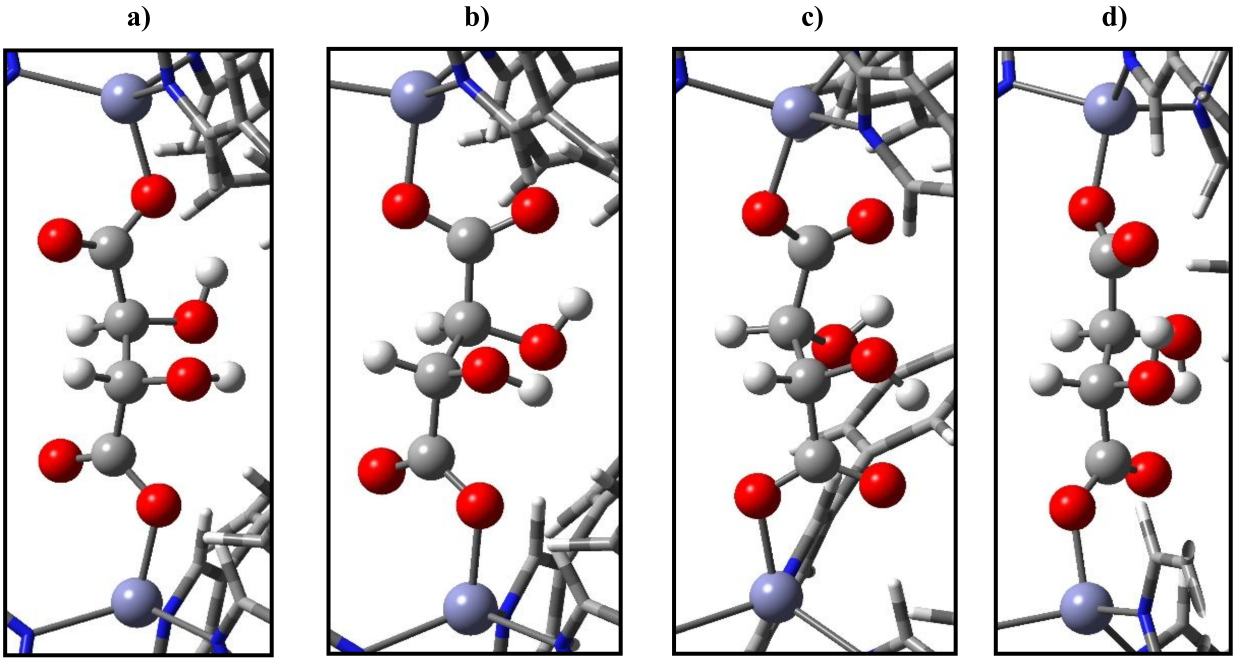

Figure S6 Most stable monodentate oxygen coordination of the D-Tartaric acid within the supramolecular cage **1** (a-d).

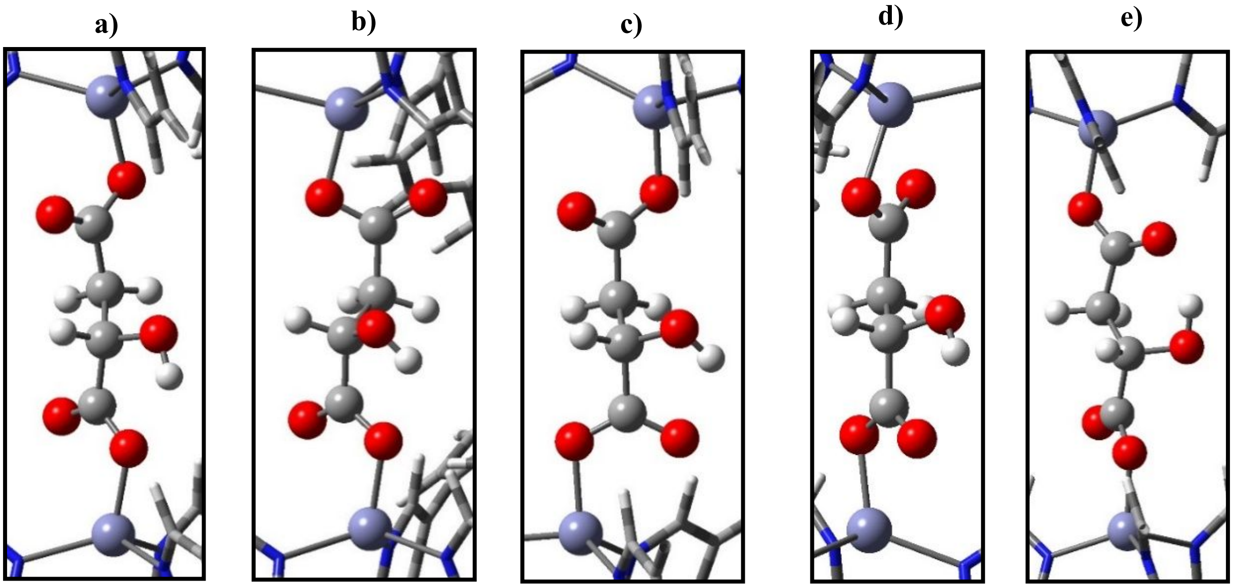

Figure S7 Most stable monodentate oxygen coordination of the L-Malic acid within the supramolecular cage **1** (a-e).

4.1.2 Meso Structures

In the last part of the computational search structures with opposite helicity of the two **TPMA** zinc complexes have been considered (pseudo-meso). The dihedral angle between the pyridine ring and the phenyl ring (Figure S4) were also manually varied while the dihedral angle of the imine bond relative to the pyridine ring (Figure S5) was kept <|90°| since the most stable conformers calculated so far had this stereoconformation. The most stable dicarboxylic acid coordination was used for the calculation, i.e. **c)** for the D-Tartaric acid (Figure S6) and **e)** for the L-Malic acid (Figure S7). The classification of the different meso conformers is shown in Table S2.

Conformers	Helicity	Dihedral Angle Pyr-Ar
IX	Δ - Λ	+ / +
X	Δ - Λ	+ / -
XI	Λ - Δ	- / -
XII	Λ - Δ	+ / -

Table S2 Classification of the four meso conformers computed: the propeller direction of **TPMA**, **Λ** (counterclockwise), **Δ** (clockwise) and the sign of dihedral angle formed between the pyridine-ring of **TPMA** and the phenyl-ring (+ for a positive angle, **-** for a negative angle)**.** The dihedral angle of the imine bond relative to the pyridine ring was kept **<|90°|**.

The conformational search over the four meso structures reveal the presence of other two good conformers, namely the IX and XI (Table S3). For both the diacid tested, they resulted close in energy to the best stable structures previously found.

4.1.3 Energy calculation of the most stable conformers

Sixteen different conformations were obtained by changing the three stereogenic elements of the cage and the diacid coordination. Relative energies are calculated by the difference from the most stable conformation energy. **D-Tar@1** conformers summarized in Table S3 and **L-Mal@1** summarized in Table S4.

Entry	D-Tar@1 Cage Conformers	Diacid Coordination	Energy	Relative Energy (kcal/mol)
1	I	a)	-8175.138165	1.4
2	II	a)	-8175.101767	24.3
3	III	a)	-8175.102165	24.0
4	IV	a)	-8175.094548	28.8
5	V	a)	-8175.138076	1.5
6	VI	a)	-8175.108308	20.2
7	VII	a)	-8175.107316	20.8
8	VIII	a)	-8175.105409	22.0
9	I	b)	-8175.139124	0.8
10	V	b)	-8175.140442	0.0
11	I	c)	-8175.137799	1.7
12	V	c)	-8175.136376	2.6
13	I	d)	-8175.138976	0.9
14	V	d)	-8175.137965	1.6
15	I	e)	-	-
16	V	e)	-	-
17	IX	b)	-8175.139461	0.6
18	X	b)	-8175.116636	14.9
19	XI	b)	-8175.138348	1.3
20	XII	b)	-8175.118944	13.5

Table S3 Energy difference calculated from the minimum energy value for each conformation (kcal/mol). Highlighted in green the lowest energetical conformation and in yellow the second and third best.

Entry	L-Mal@1 Cage Conformers	Diacid Coordination	Energy	Relative Energy (kcal/mol)
1	I	a)	-8099.925810	1.9
2	II	a)	-8099.888734	25.2
3	III	a)	-8099.894008	21.9
4	IV	a)	-8099.893184	22.4
5	V	a)	-8099.925888	1.9
6	VI	a)	-8099.899173	18.7
7	VII	a)	-8099.897083	20.0
8	VIII	a)	-8099.895155	21.2
9	I	b)	-8099.921843	4.4
10	V	b)	-8099.923900	3.1
11	I	c)	-8099.927789	0.7
12	V	c)	-8099.926030	1.8
13	I	d)	-8099.921695	4.5
14	V	d)	-8099.920683	5.2
15	I	e)	-8099.928891	0.0
16	V	e)	-8099.928610	0.2
17	IX	e)	-8099.926993/ -8099.925047[a]	1.2 / 2.4[a]
18	X	e)	-8099.905595/ -8099.904361[a]	14.6 / 15.4[a]
19	XI	e)	-8099.926250/ -8099.926511[a]	1.7 / 1.5[a]
20	XII	e)	-8099.916133/ -8099.924247[a]	8.0 / 2.9[a]

Table S4 Energy difference calculated from the minimum energy value for each conformation (kcal/mol). Highlighted in green the lowest energetical conformation and in yellow the second and third best.

[a)]: two coordination of the malic acid are possible, the left one with the hydroxyl group pointing the **Δ** conformation of the zinc complex, the right one pointing the **Λ**.

Table S3 and Table S4 summarize the conformational search conducted for the supramolecular cages **D-Tar@1** and **L-Mal@1**. As it is possible to see, there are some conformations relatively close in energy and others considerably far from the best one. To consider that the energy difference is expressed in kcal/mol, and therefore a small value difference determines a relatively large difference in the Boltzmann population. As example, considering two best structures with an energy difference equal to 0.5 kcal/mol, their corresponding population will be 70% for the best conformation and 30% for the second one.

In the case of **L-Mal@1**, two structures resulted significantly close in energy (Entry 15 and 16). Noteworthy, these two conformations are characterized by an opposite **TPMA** helicity and thus opposite CD spectra. As a result, the contemporary presence of both the conformations in solution, could be the explanation of the low intensity of the experimental CD signal measured for this system.

4.2 B3LYP/6-31G(d) Best minimized structures

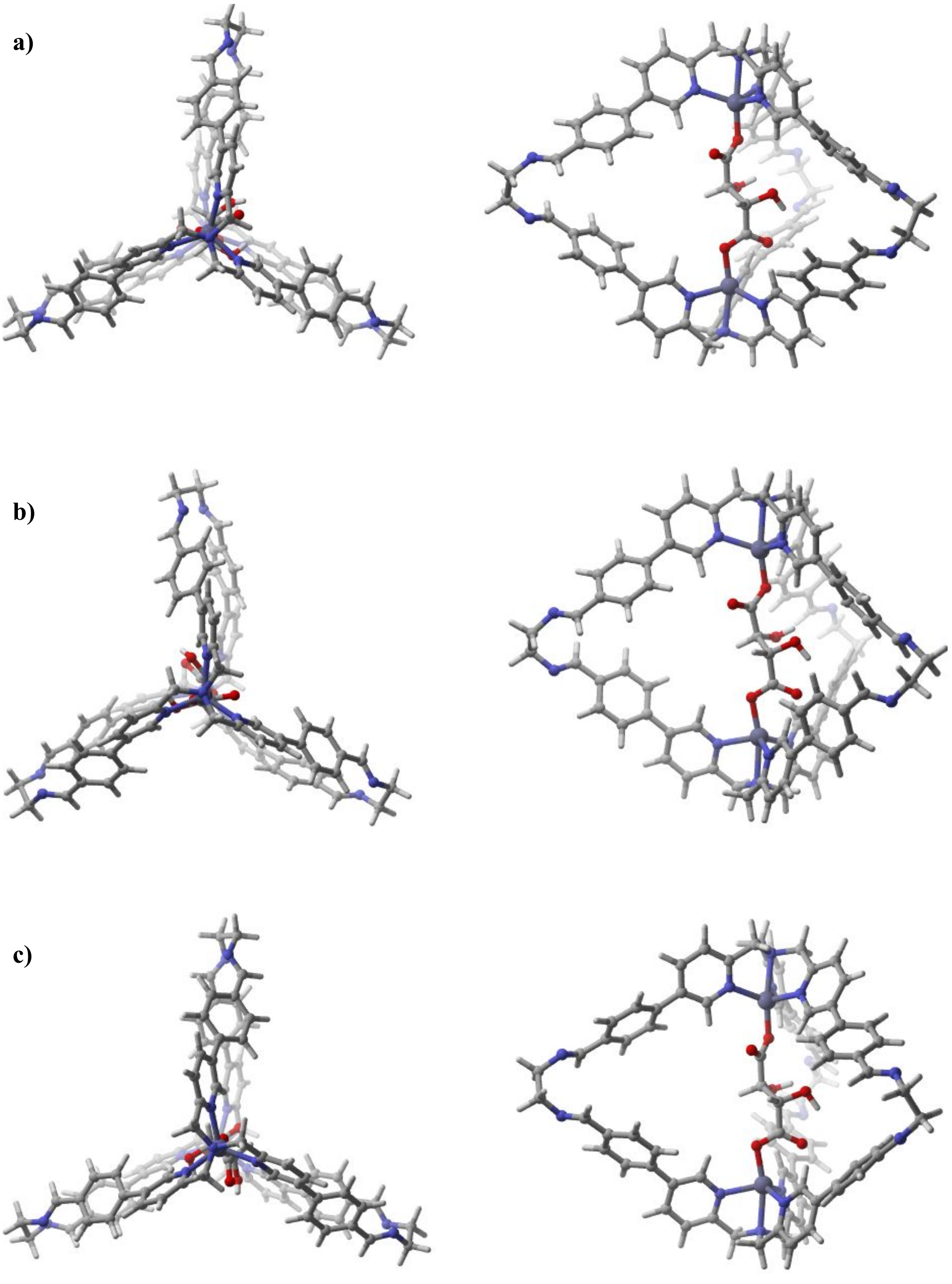

Figure S8. B3LYP/6-31G(d) minimized structures for the cage **D-Tar@1** a) entry 10, b) entry 17, c) entry 9.

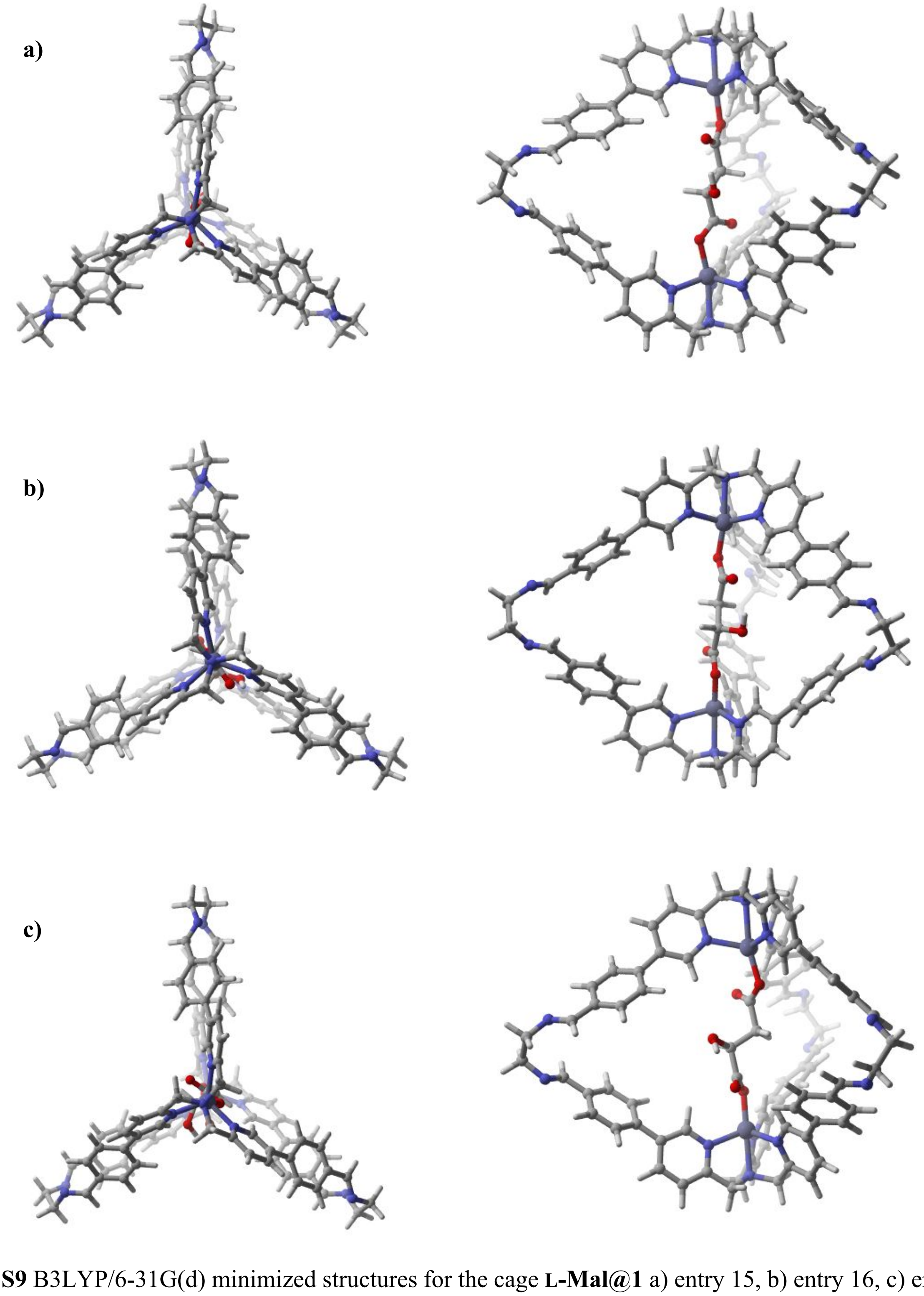

Figure S9 B3LYP/6-31G(d) minimized structures for the cage **L-Mal@1** a) entry 15, b) entry 16, c) entry 11.

4.3 TD-DFT Calculations

Once obtained the energy distribution of the conformers, TD-DFT B3LYP/6-31G(d) calculations were performed to simulate the CD spectrum. The calculations were carried out over 50 excited states for the three best conformational structures found for the supramolecular systems **D-Tar@1** and **L-Mal@1** and Boltzmann-weighted ECD spectrum was then obtained with SpecDis software,[5] by using the population data shown in Table S5 and the bandwidth σ that gives the best superimposition of the spectra. To do this, the program maximize the *similarity factor S*, a parameter proposed by Bultinck[6] based on the cosine similarity equation.

**D-Tar@1**		
**Entry**	**ΔG (kcal/mol)**	**Population (%)**
10	0.0	62
17	0.6	22
9	0.8	16

**L-Mal@1**		
**Entry**	**ΔG (kcal/mol)**	**Population (%)**
15	0.0	49
16	0.2	36
11	0.7	15

Table S5 Energy difference in kcal/mol with their respective population (%) for the three best structures of the supramolecular systems **D-Tar@1** and **L-Mal@1**.

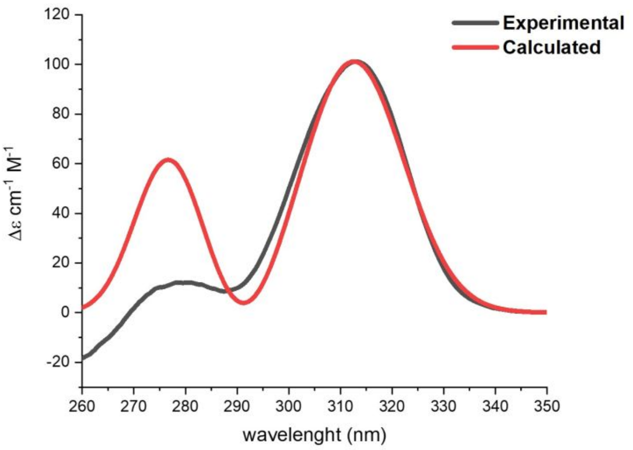

Figure S10 Experimental (black) molar ellipticity and calculated (red) CD spectra of **D-Tar@1**cage. The UV-shift and the best fitting bandwidth are equal to -29 nm and 0.15 eV respectively. The scale ratio experimental:calculated is 1:0.3521.

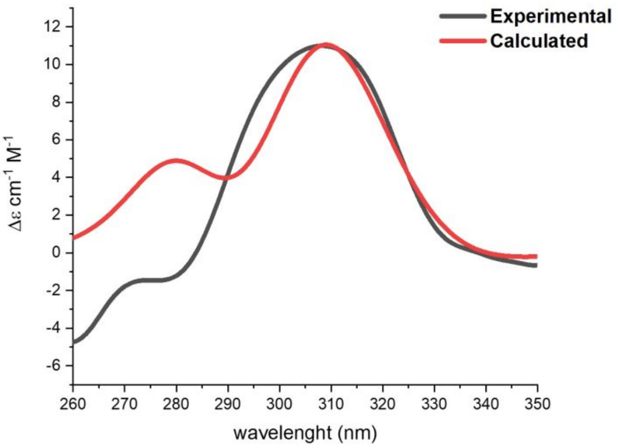

Figure S11 Overlay of experimental molar ellipticity and calculated CD spectra of **L-Mal@1** cage. The UV-shift and the best fitting bandwidth are equal to -29 nm and 0.20 eV respectively. The scale ratio experimental:calculated is 1:0.7724.

5 Calibration Curves for the different wines

General Procedure for the Standard addition Method

To 5 vials containing 500 µL (1.0 µmol) of a solution 0.002 M of the Zinc complex **2** in DMSO, 10 µL (Prosecco, Müller Thurgau) or 5 µL (Chardonnay, Chianti, Barbera, Valpolicella) of wine were added in each vial. Then, increasing amount of a solution 0.005 M of **L-Tar** in DMSO were added. Finally, 125 µL (2.5 µmol) of a solution 0.02 M in DMSO of ethylenediamine were added. For the CD analysis, 150 µL of the mixture were diluted to 1 mL with dry DMSO and analyzed. The measurements were repeated three times.

The standard deviation has been calculated using the equation:

$$s_x = \frac{t \cdot s_{y/x}}{m}\sqrt{\frac{1}{N} + \frac{(\bar{y})^2}{m^2 \sum (x_i - \bar{x})^2}}$$

5.1 Prosecco

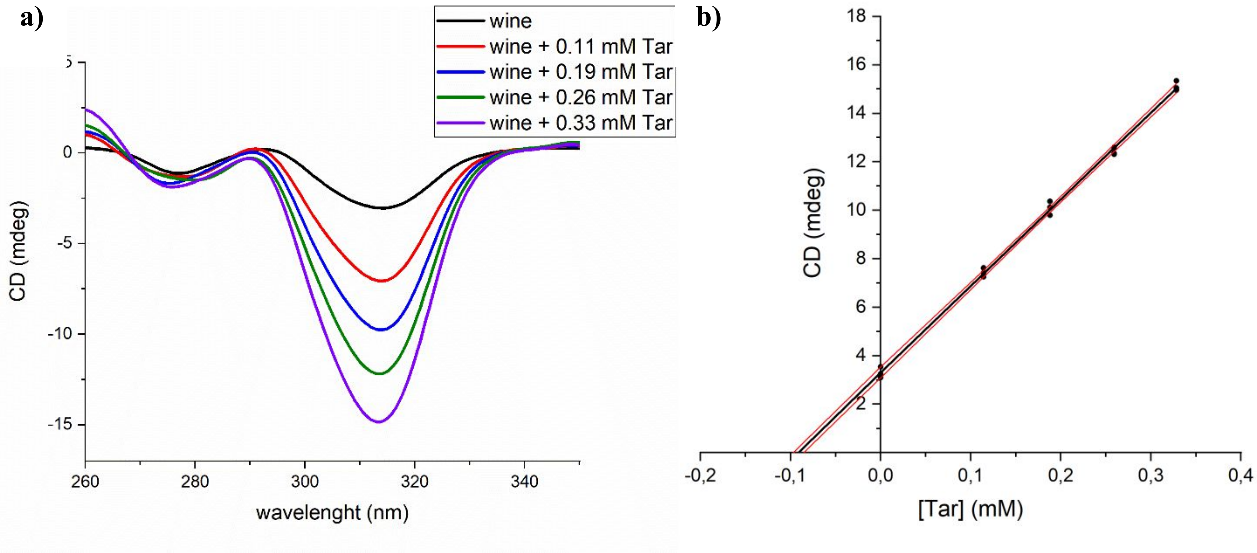

Figure S12 a) Circular Dichroism spectra in Ellipticity (mdeg) within the range 260-350 nm of the supramolecular system **1** with 10 µL of Prosecco wine and different content in L-tartaric acid, respectively equal to 0.0x10^{-4} M (black line), 1.1x10^{-4} M (red line), 1.9x10^{-4} M (blue line), 2.6x10^{-4} M (green line), 3.3x10^{-4} M (violet line); b) the linear fit of the CD signal response of the supramolecular system **2** taking the point of the maximum CD signal at 314 nm. The fitting equation curve is y=35.72x+3.31, R^2=0.997. The abscissa intercept is equal to -0.093 ± 0.008 mM, corresponding to a Tartaric acid content equal to 1.11 ± 0.05 g/L.

5.2 Chianti

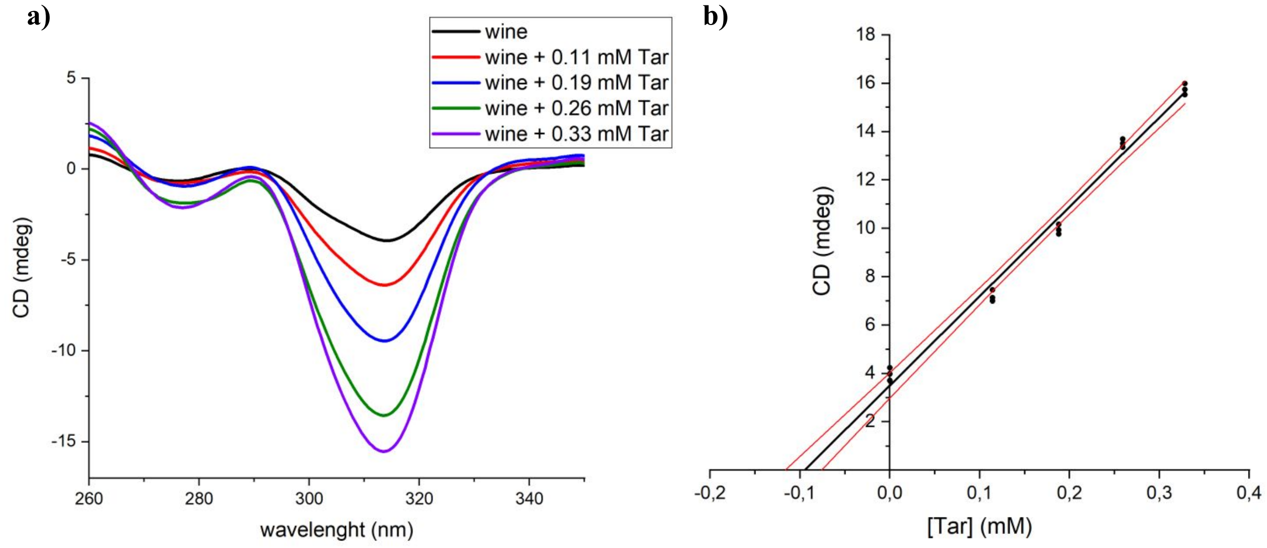

Figure S13 a) Circular Dichroism spectra in Ellipticity (mdeg) within the range 260-350 nm of the supramolecular system **1** with 5 μL of Chianti wine and different content in L-tartaric acid, respectively equal to 0.0×10^{-4} M (black line), 1.1×10^{-4} M (red line), 1.9×10^{-4} M (blue line), 2.6×10^{-4} M (green line), 3.3×10^{-4} M (violet line); b) the linear fit of the CD signal response of the supramolecular system **2** taking the point of the maximum CD signal at 314 nm. The fitting equation curve is $y=36.91x+3.50$, $R^2=0.986$. The abscissa intercept is equal to -0.095 ± 0.020 mM, corresponding to a Tartaric acid content equal to 2.28 ± 0.13 g/L.

5.3 Chardonnay

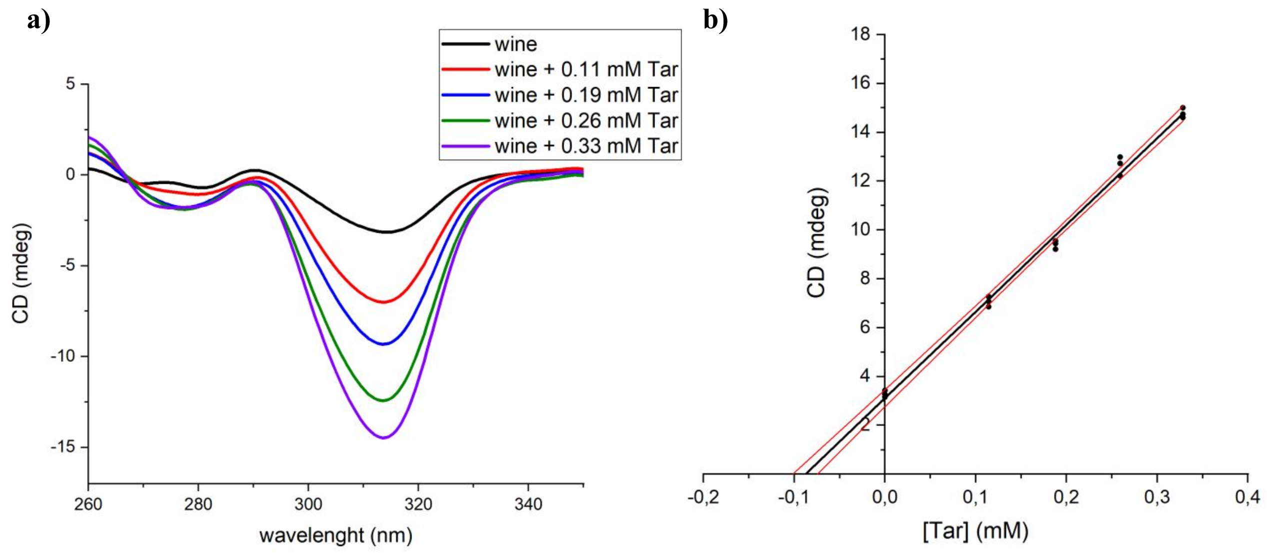

Figure S14 a) Circular Dichroism spectra in Ellipticity (mdeg) within the range 260-350 nm of the supramolecular system **1** with 5 μL of Chardonnay wine and different content in L-tartaric acid, respectively equal to 0.0×10^{-4} M (black line), 1.1×10^{-4} M (red line), 1.9×10^{-4} M (blue line), 2.6×10^{-4} M (green line), 3.3×10^{-4} M (violet line); b) the linear fit of the CD signal response of the supramolecular system **2** taking the point of the maximum CD signal at 314 nm. The fitting equation curve is $y=35.51x+3.11$, $R^2=0.993$. The abscissa intercept is equal to -0.087 ± 0.013 mM, corresponding to a Tartaric acid content equal to 1.68 ± 0.09 g/L.

5.4 Valpolicella

a) b)

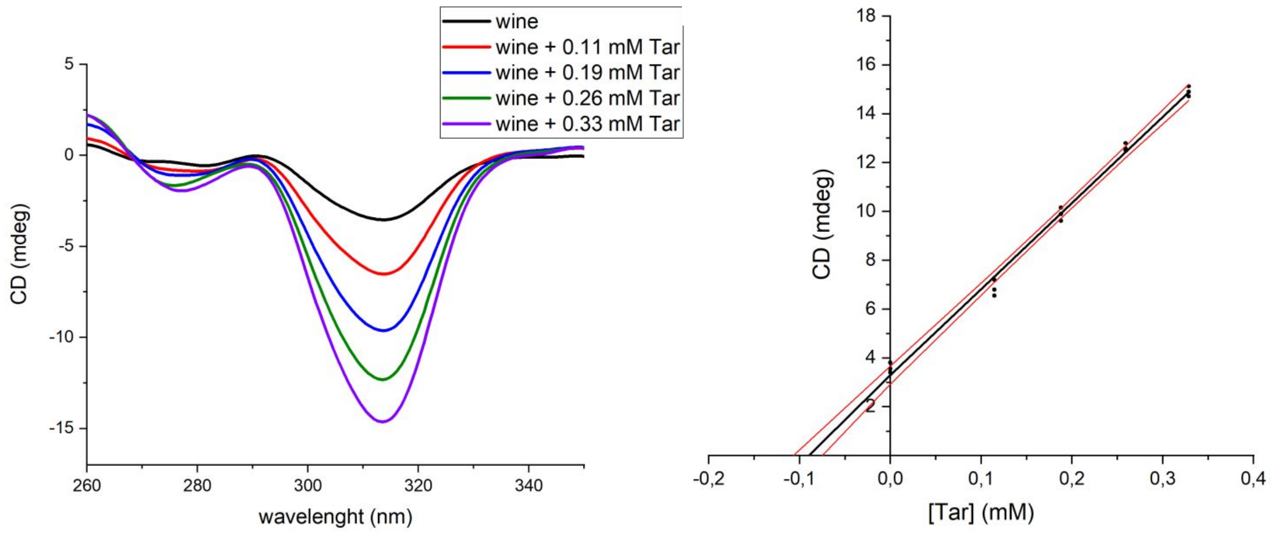

Figure S15 a) Circular Dichroism spectra in Ellipticity (mdeg) within the range 260-350 nm of the supramolecular system **1** with 5 µL of Valpolicella wine and different content in L-tartaric acid, respectively equal to $0.0x10^{-4}$ M (black line), $1.1x10^{-4}$ M (red line), $1.9x10^{-4}$ M (blue line), $2.6x10^{-4}$ M (green line), $3.3x10^{-4}$ M (violet line); b) the linear fit of the CD signal response of the supramolecular system **2** taking the point of the maximum CD signal at 314 nm. The fitting equation curve is y=35.24x+3.30, R^2=0.991. The abscissa intercept is equal to -0.094 ± 0.015 mM, corresponding to a Tartaric acid content equal to 2.24 ± 0.09 g/L.

5.5 Müller-Thurgau

a) b)

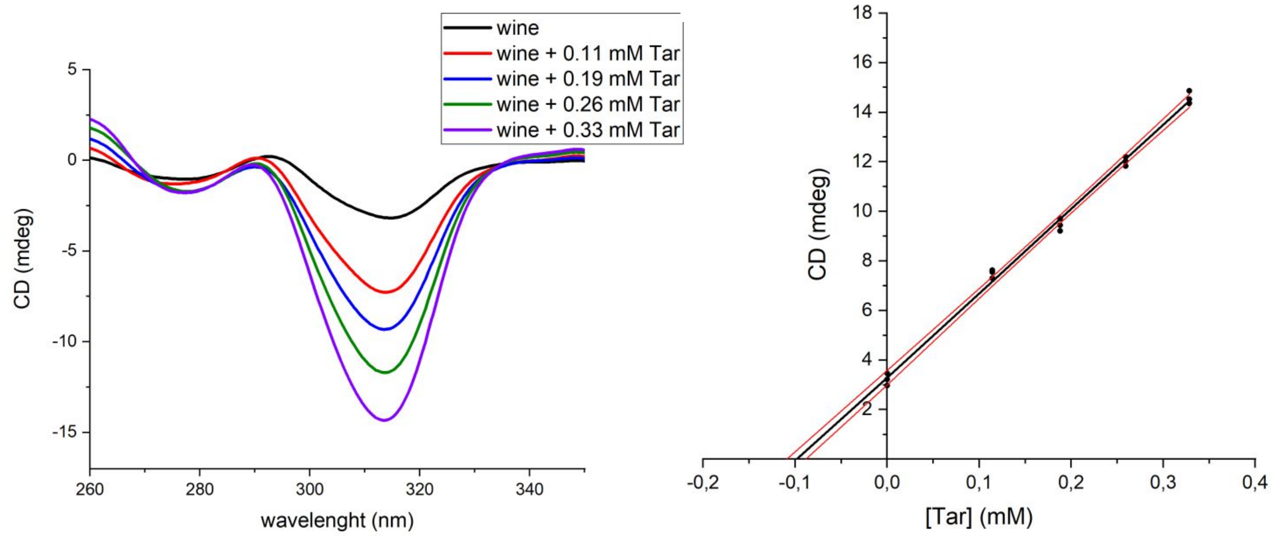

Figure S16 a) Circular Dichroism spectra in Ellipticity (mdeg) within the range 260-350 nm of the supramolecular system **1** with 10 µL of Müller-Thurgau wine and different content in L-tartaric acid, respectively equal to $0.0x10^{-4}$ M (black line), $1.1x10^{-4}$ M (red line), $1.9x10^{-4}$ M (blue line), $2.6x10^{-4}$ M (green line), $3.3x10^{-4}$ M (violet line); b) the linear fit of the CD signal response of the supramolecular system **2** taking the point of the maximum CD signal at 314 nm. The fitting equation curve is y=34.04x+3.29, R^2=0.995. The abscissa intercept is equal to -0.097± 0.012 mM, corresponding to a Tartaric acid content equal to 1.16 ± 0.08 g/L.

5.6 Barbera

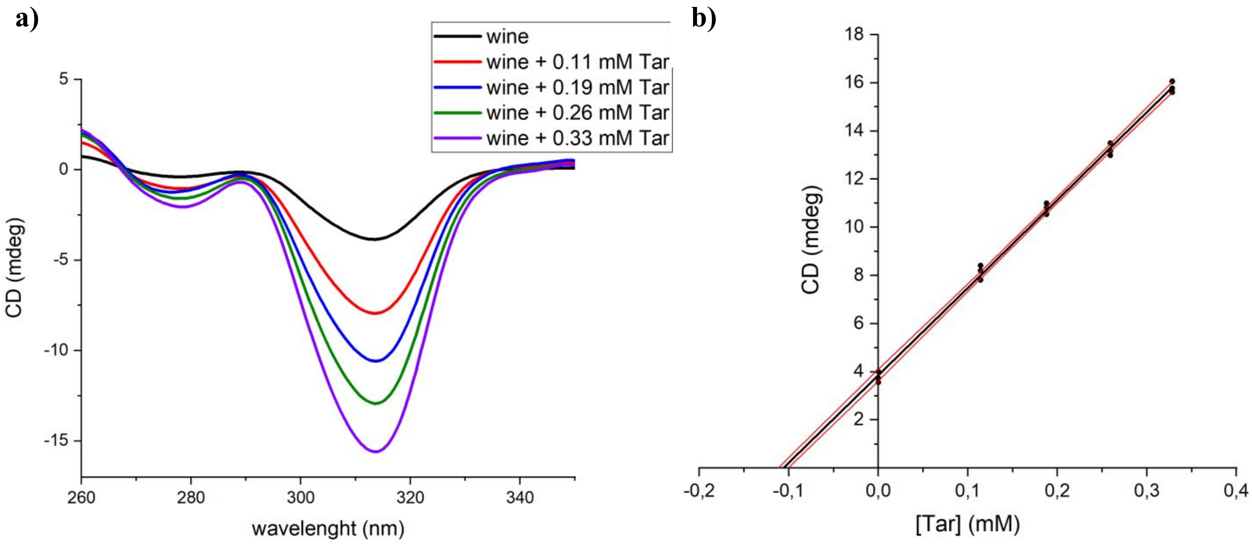

Figure S17 a) Circular Dichroism spectra in Ellipticity (mdeg) within the range 260-350 nm of the supramolecular system **1** with 5 µL of Barbera wine and different content in L-tartaric acid, respectively equal to 0.0x10^{-4} M (black line), 1.1x10^{-4} M (red line), 1.9x10^{-4} M (blue line), 2.6x10^{-4} M (green line), 3.3x10^{-4} M (violet line); b) the linear fit of the CD signal response of the supramolecular system **2** taking the point of the maximum CD signal at 314 nm. The fitting equation curve is y=36.44x+3.85, R^2=0.997. The abscissa intercept is equal to -0.106± 0.010 mM, corresponding to a Tartaric acid content equal to 2.54 ± 0.06 g/L.

6 NMR and ESI-MS characterization

6.1 L-Tar@1

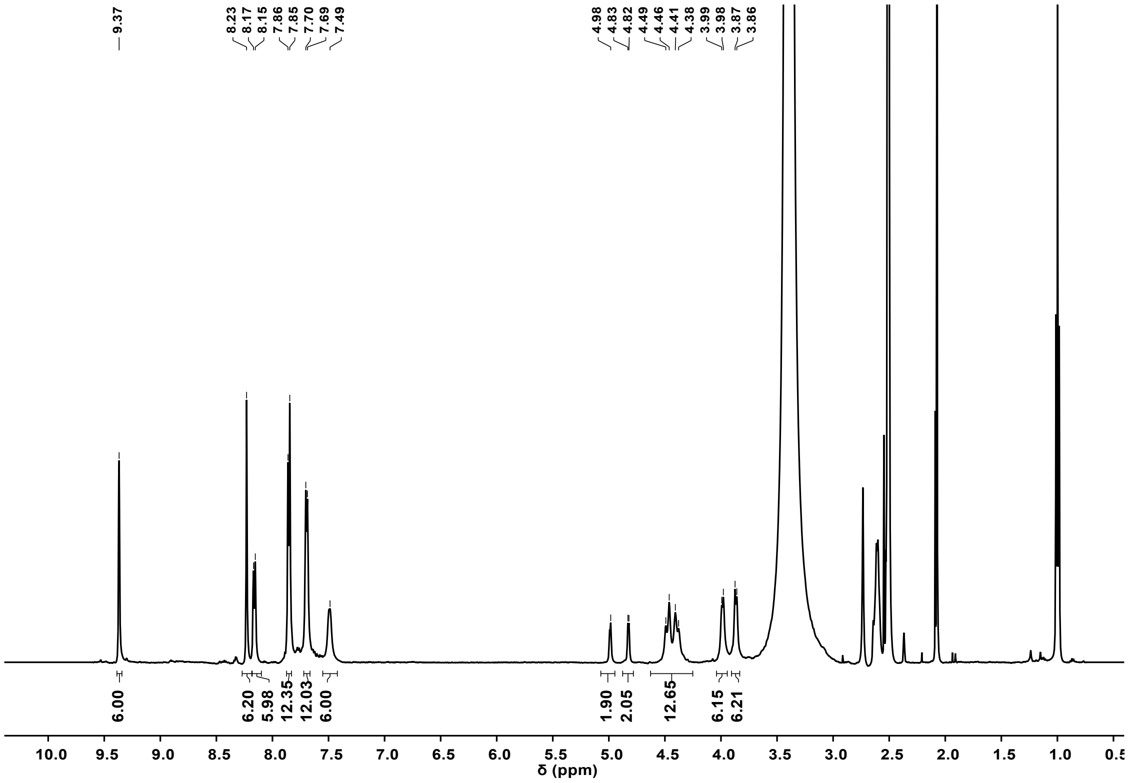

Figure S18 ^{1}H-NMR spectrum (500 MHz, 301 K, DMSO-d_6) of cage L-**Tar@1**.

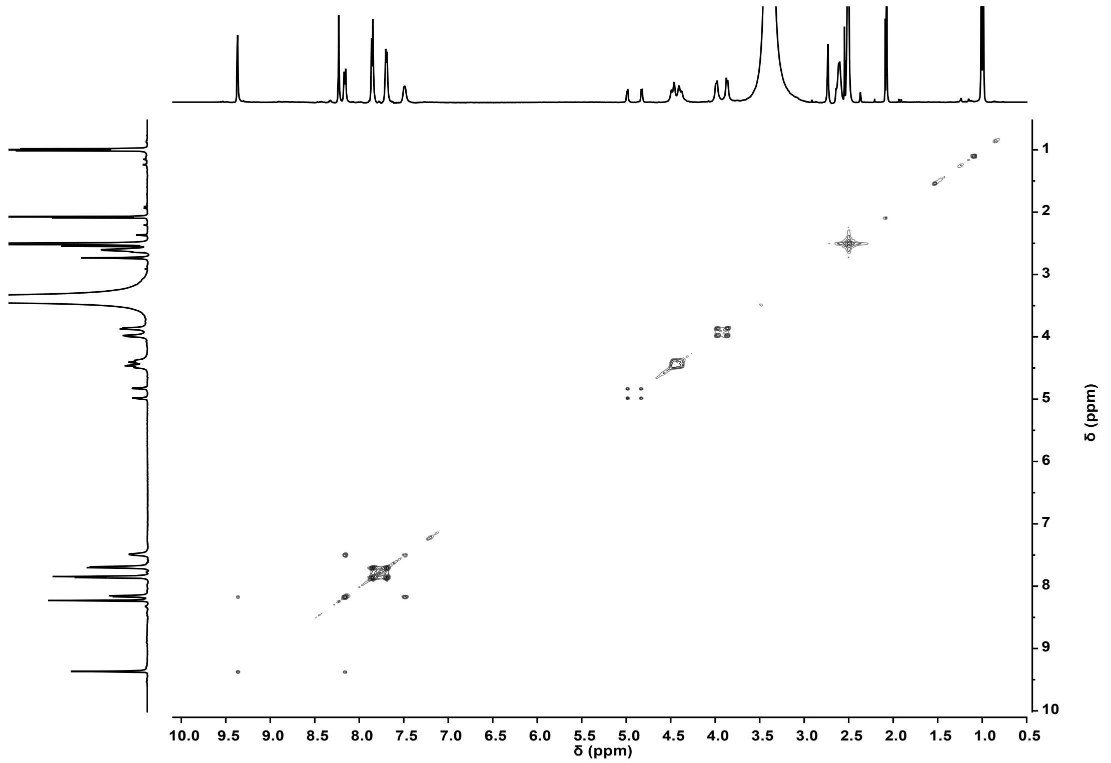

Figure S19 ^{1}H-^{1}H COSY spectrum (500 MHz, 301 K, DMSO-d_6) of cage L-**Tar@1**.

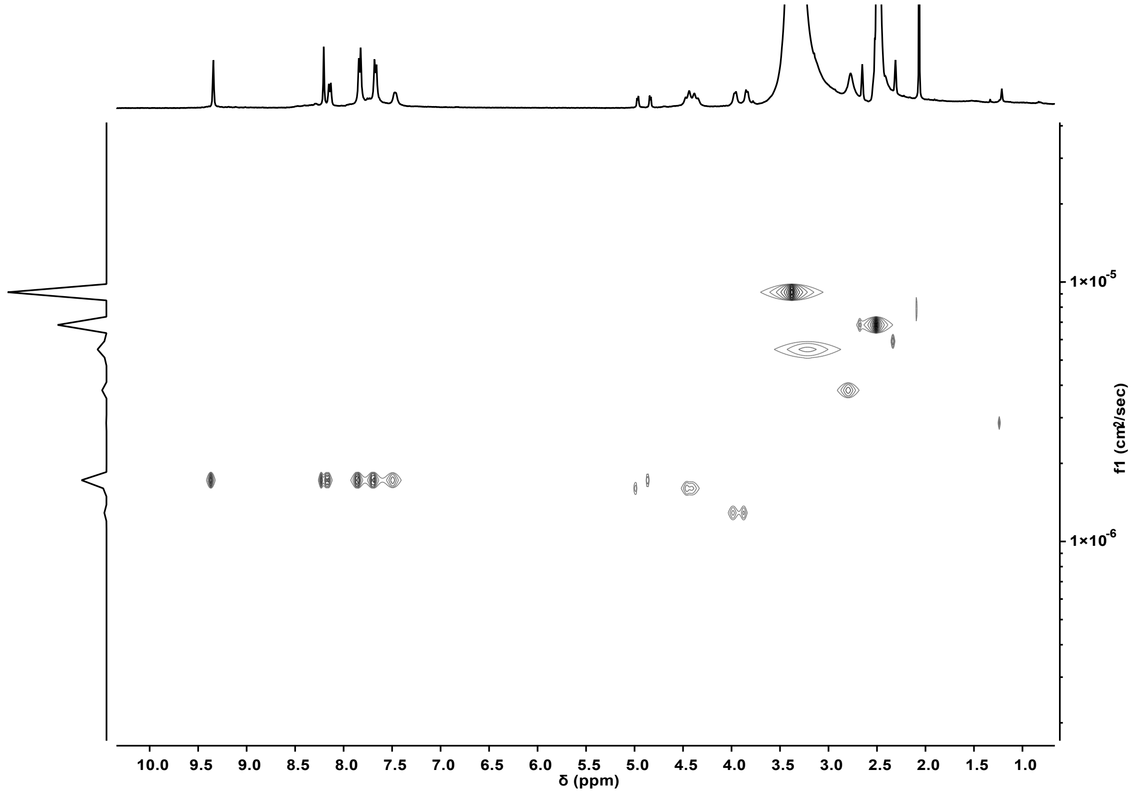

Figure S20 DOSY spectrum (400 MHz, 301 K, DMSO-d_6) of cage L-**Tar@1**. The diffusion coefficient for the molecular cage **Tar@1** was calculated to be 1.63 x 10^{-10} m^2 s^{-1}, corresponding to a hydrodynamic radius (r_H) of 9.8 Å. The hydrodynamic radius was calculated using Stokes-Einstein equation.[7,8]

6.2 L-Mal@1

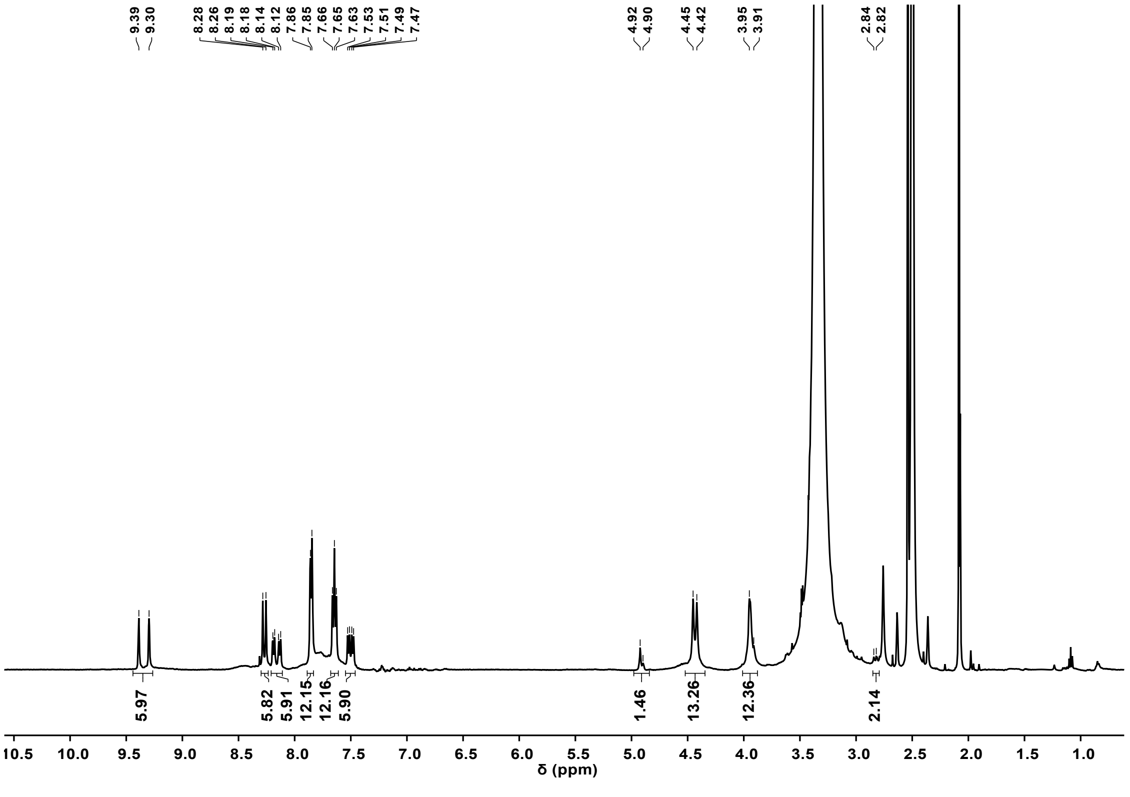

Figure S21 ^{1}H-NMR spectrum (500 MHz, 301 K, DMSO-d_6) of cage L-**Mal@1**.

6.3 *R,S*-Cam@1

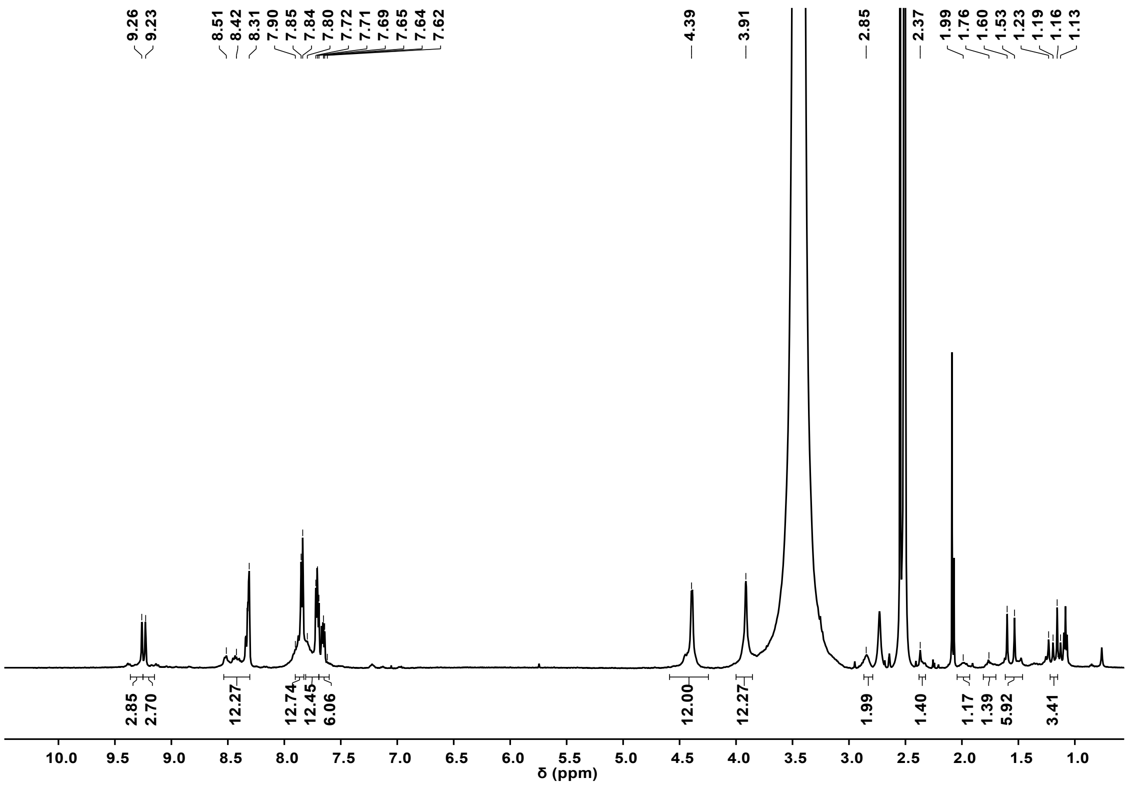

Figure S22 ^{1}H-NMR spectrum (500 MHz, 301 K, DMSO-d_6) of cage ***R,S*-Cam@1**.

6.4 L-Glu@1

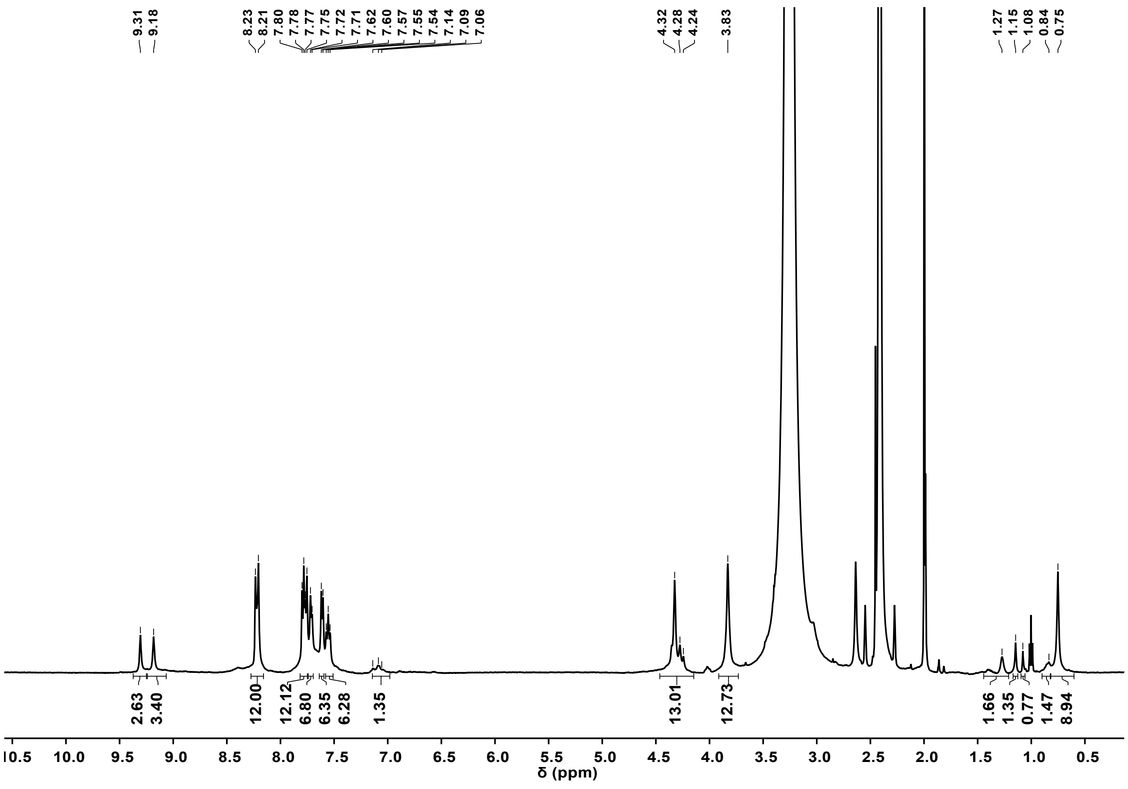

Figure S23 ^{1}H-NMR spectrum (500 MHz, 301 K, DMSO-d_6) of cage **L-Glu@1**.

6.5 L-Asp@1

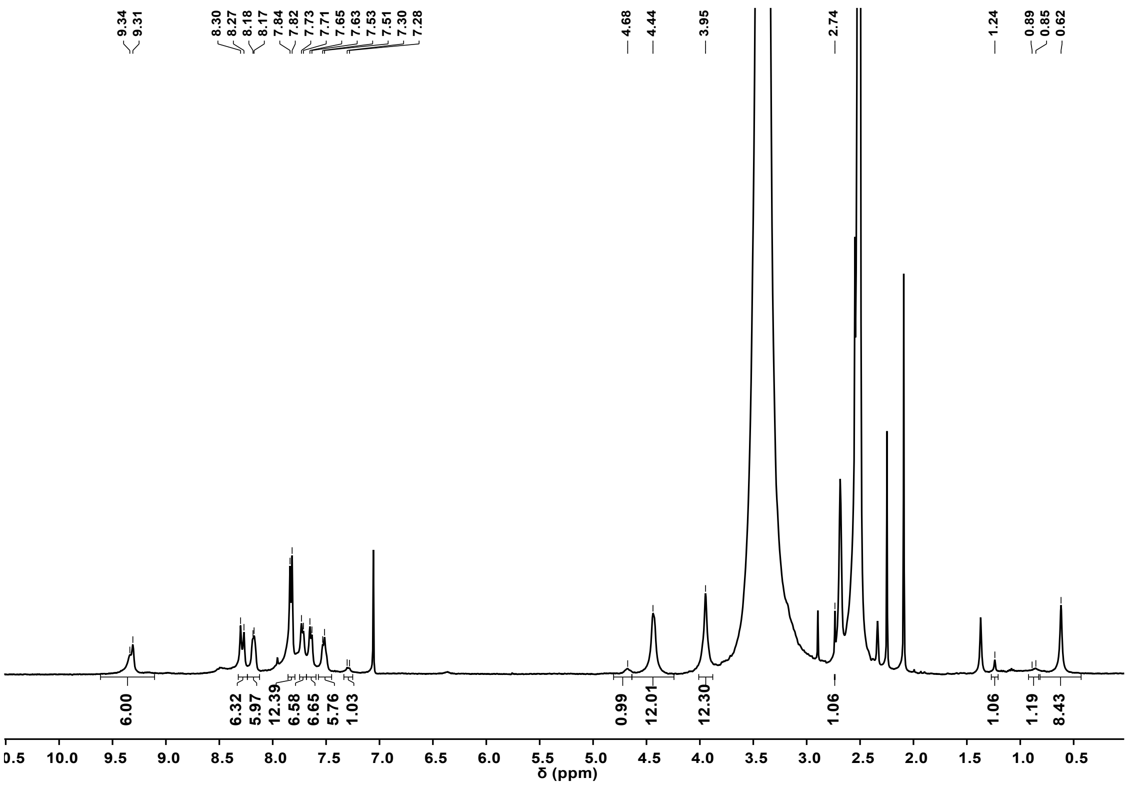

Figure S24 ^{1}H-NMR spectrum (500 MHz, 301 K, DMSO-d_6) of cage **L-Asp@1**.

6.6 ESI-MS spectrum L-Tar@1

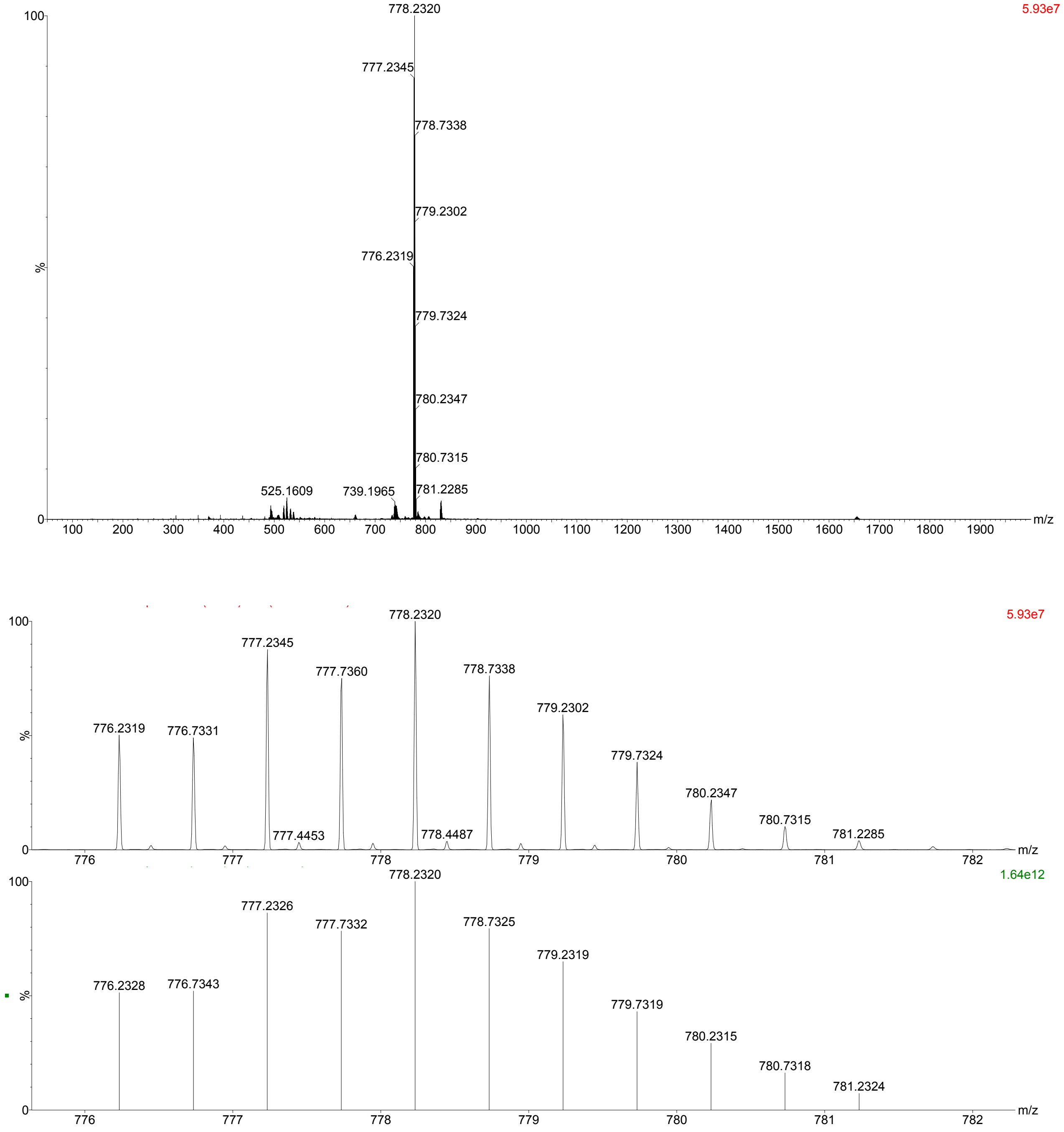

Figure S25 Experimental (top), and calculated (bottom) HRMS (ESI-TOF) pattern of **l-Tar@1** corresponding to $[C_{88}H_{76}N_{14}O_6Zn_2]^{2+}$ in CH_3CN/0.1% HCOOH.

7 Binding constant determination

To 500 µL (0.5 µmol) of a solution 0.001 M of the cage **1** (based on *p*-xylene standard) in DMSO-d_6, 30 µL (0.36 µmol) of a solution 0.012 M in DMSO-d_6 of *p*-xylene were added. Small aliquots of the guest solution were titrated into this NMR tube. The solution was allowed to equilibrate at room temperature during 30 seconds before acquiring the ^{1}H NMR spectrum.

Due to the slow exchange regime, the determination of binding constant was possible thanks to the direct integration of the signals of the pyridine ring α proton of the filled cage and the empty cage in the region between 9.5 ppm and 8.5 ppm using equation:

$$K_b = \frac{[HG]}{[H][G]}$$

where the concentrations of [H] and [HG] have been determined by direct integration of the peaks, [G] has been determined by difference between added $[G]_0$ and measured [HG]. The NMR spectrum of the empty cage shows unsymmetric signals in DMSO. In particular, in the region of the pyridine ring α proton 9.3 ppm and 8.5 ppm. [H] has been determined by the sum of the integrals of this region. Reported binding constants are the average value of the four binding constants measure after addition of 0.2, 0.4, 0.6 and 0.8 equivalents.

The binding constant values for each dicarboxylic acid (**L-Tar** and **L-Mal**) are displayed in Table S6.

**Diacid**	**K_b (M^{-1})**	**± σ (M^{-1})**
**L-Tar**	$1.1 \cdot 10^5$	$1.7 \cdot 10^4$
**L-Mal**	$1.3 \cdot 10^5$	$2.3 \cdot 10^4$

Table S6 Binding constant values for **L-Tar** and **L-Mal** acids.

7.1 Titration Experiment of L-Tartaric Acid with Molecular Cage 1

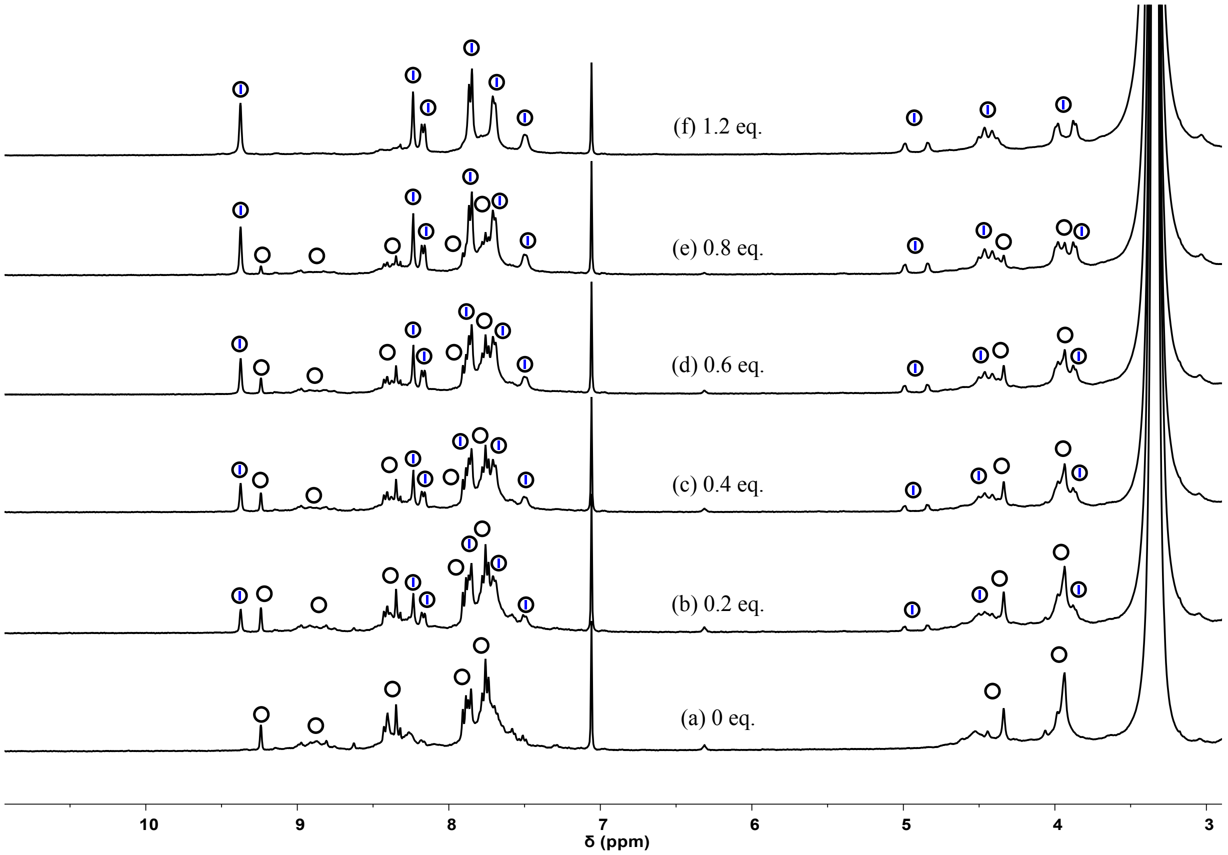

Figure S26 ^{1}H-NMR inclusion experiments (400 MHz, 301 K, DMSO-d_6). Addition of **L-Tar** in the preformed cage **1** in DMSO-d_6. The black circle indicates cage **1** blue stick indicates the dicarboxylic acid **L-Tar**. (a) Preformed cage **1** (0.001 M cage). (b)-(e) Addition of sub-stoichiometric amounts (0.2-0.8 equiv) of **L-Tar** results in the formation of a new species which could be attributed to 1:1 H:G complex. (f) Addition of 1.2 equiv of **L-Tar** totally shift the system to the new species **L-Tar@1**.

7.2 Titration Experiment of L-Malic Acid with Molecular Cage 1

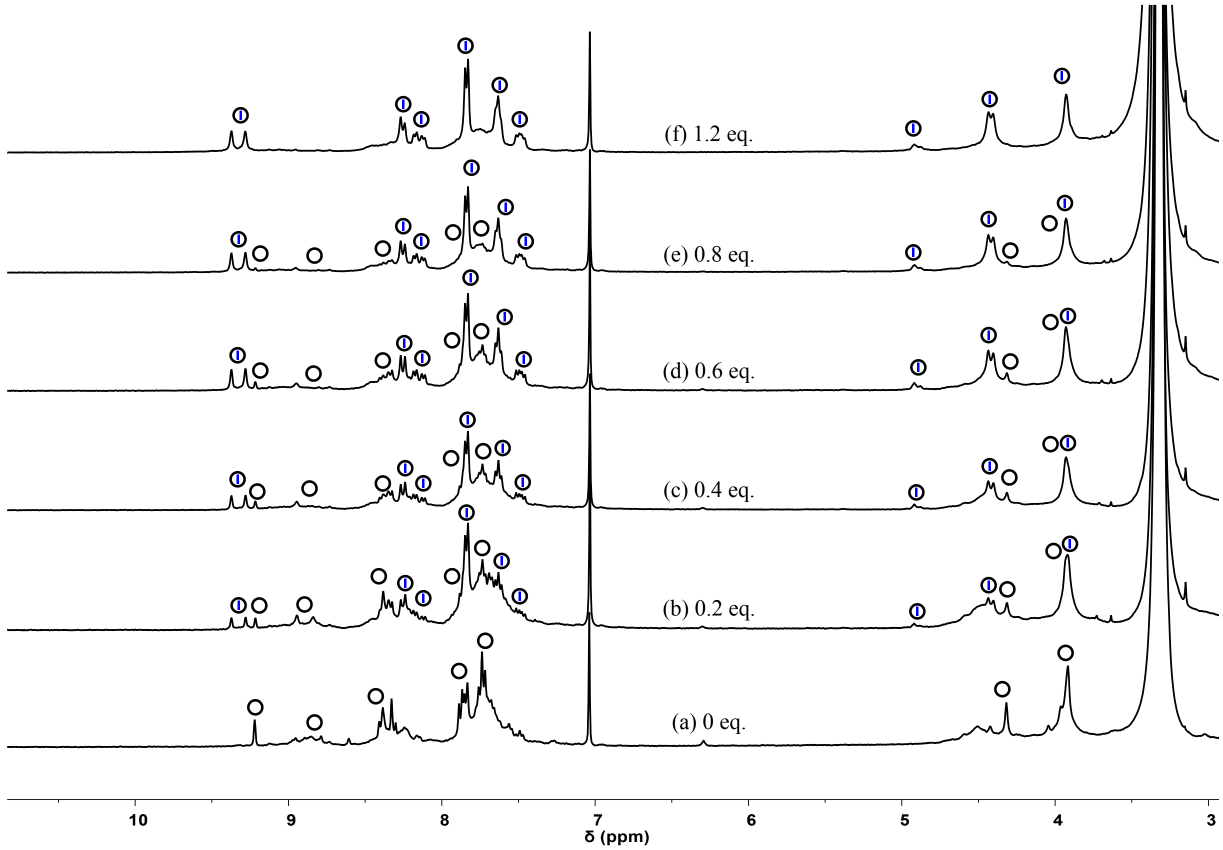

Figure S27 ^{1}H-NMR inclusion experiments (400 MHz, 301 K, DMSO-d_6). Addition of L-**Mal** in the preformed cage **1** in DMSO-d_6. The black circle indicates cage **1** blue stick indicates the dicarboxylic acid L-**Mal**. (a) Preformed cage **1** (0.001 M cage). (b)-(e) Addition of sub-stoichiometric amounts (0.2-0.8 equiv) of L-**Mal** results in the formation of a new species which could be attributed to 1:1 H:G complex. (f) Addition of 1.2 equiv of L- **Mal** totally shift the system to the new species L-**Mal@1**.

8 Kinetic Experiment of Cage Formation Using Wine as Template

To 500 µL (1.0 µmol) of a solution 0.002 M of the zinc complex **2** in DMSO-d_6, 15 µL of red wine without pre-treatment and 125 µL (2.5 µmol) of a solution 0.02 M in DMSO-d_6 of ethylenediamine were added in a NMR tube. The cage formation was checked *via* ^{1}H-NMR and the complete assembly and encapsulation of **L-Tar** acid was observed after 20 minutes.

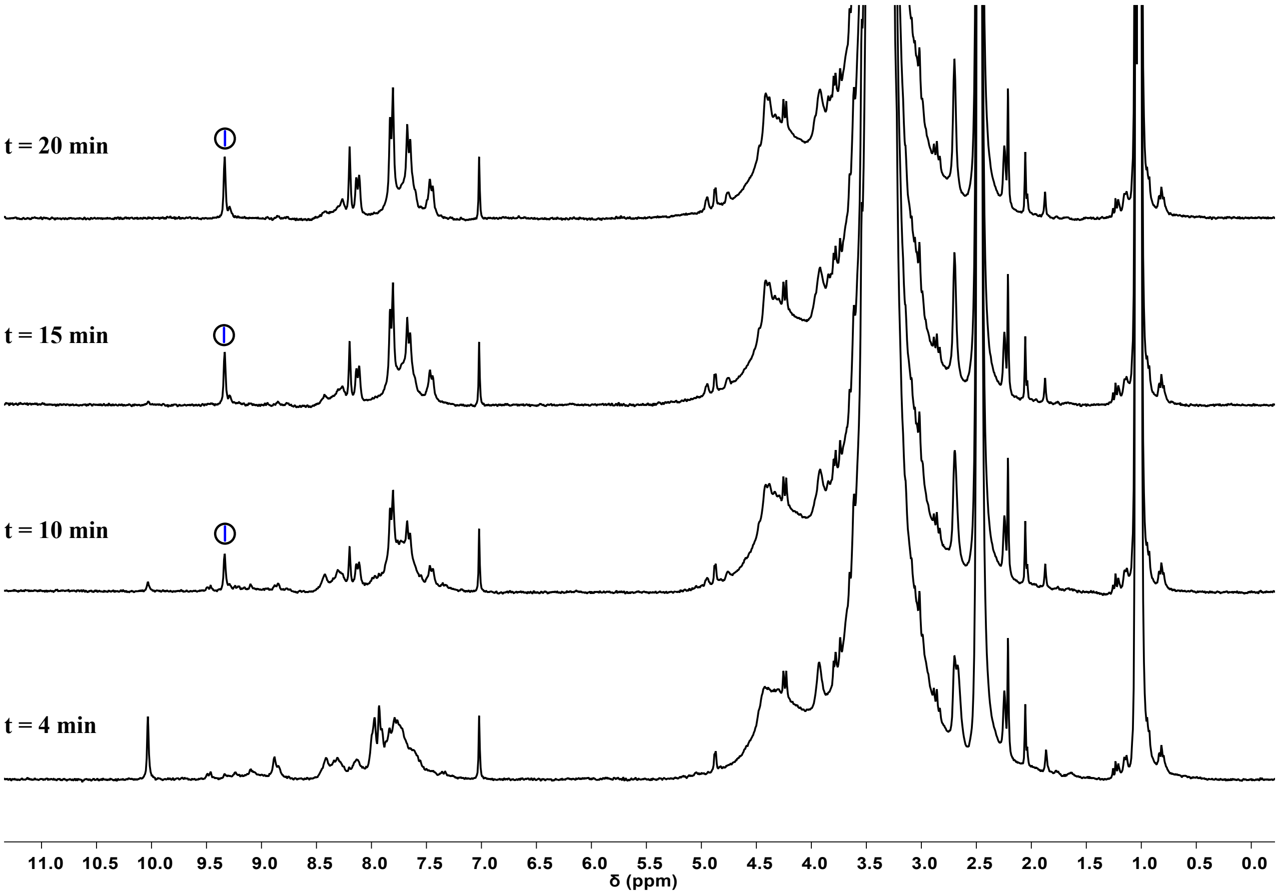

Figure S28 ^{1}H-NMR (300 MHz, 301 K, DMSO-d_6) of the cage formation at different times. The black circle indicates cage **1**, blue stick indicates the dicarboxylic acid **L-Tar**.

9 Procedure for CD Data analysis with PCA

General Procedure

To 500 µL (1.0 µmol) of a solution 0.002 M of zinc complex **2** in DMSO-d_6, 15 µL (0.5 µmol) of complex mixture and 125 µL (2.5 µmol) of a solution 0.02 M in DMSO-d_6 of ethylenediamine were added. The mixture was left for 12 hours at room temperature.
For the CD analysis, 200 µL of the mixture were diluted to 1 mL with dry DMSO and analyzed.
As complex mixtures, 3 red wines, 3 white wines, 3 Apple juices, 3 Pear juices, 3 Blueberry juices, 1 orange juice, and 1 lemon juice have been employed.
All the different mixtures were purchased at the local market in Padova.

The CD spectra were processed with Spectra Manager Version 2.15.3.1 and transformed into an xy coordinate sheet file. The non-vanishing portion of the spectra relative to the interval between 285 – 335 ppm were selected and analyzed with OriginPro 2018 (64-bit) SR1 b9.5.1.195. The data set was represented by a $n * m$ matrix X, where the n rows represent the number of points acquired during the measurement and the m columns represented the experiments characterized by a different complex mixture. In detail, the matrix was composed by a n=101 rows relative to the points of the spectra and m =13 columns relative to the number of complex mixtures utilized. Once the matrix was created, the Principal Component Analysis (PCA) was performed through the PCA for Spectroscopy script implemented into Origin which performs principal component analysis for spectra (IR, Fluorescence, UV-Vis, Raman, etc.). This tool allows to easily obtain a loading score as a plot in function of the PC1 (or PC2) and the wavelength of the spectra. In the case of PCA obtained from spectra data this kind of loading score result much clear and informative since the high number of the data points make the usual loading score (PC1 vs PC2) too crowded and complicated to understand.
The resulting PCA bidimensional plots were obtained from the score and coeff matrix from the pca function, considering the first and second principal components which account for the 99.6% of the total variance of the data set.

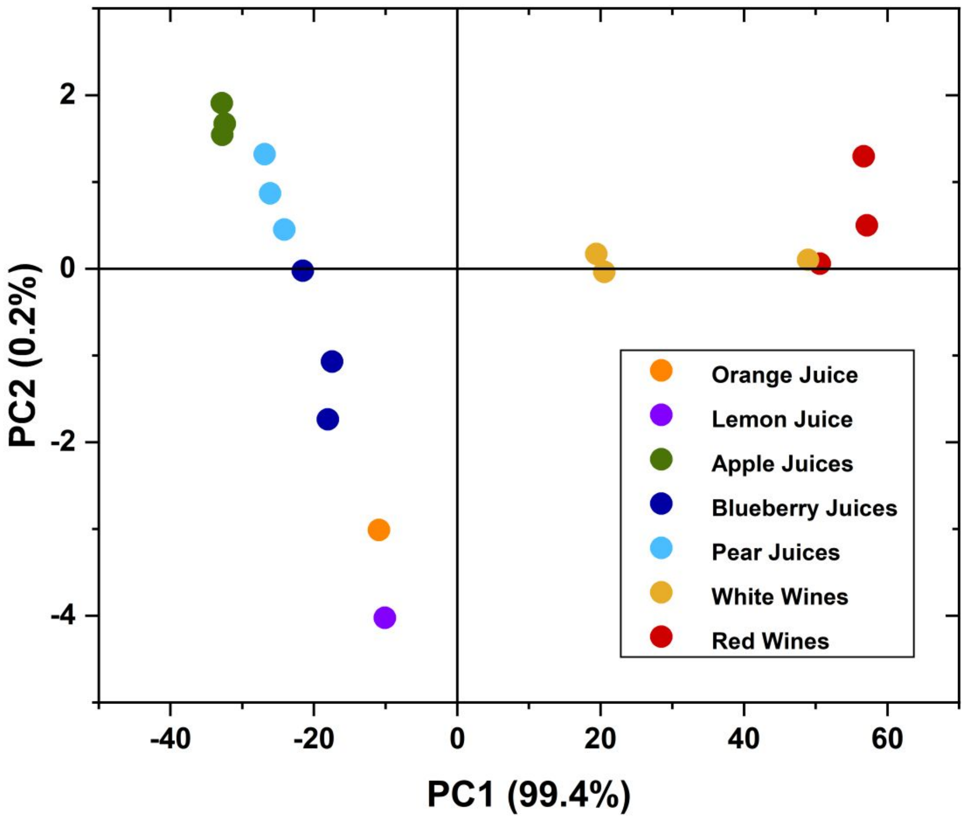

Figure S29 Scores plot of the first two principal components of the CD intensities matrix PCA.

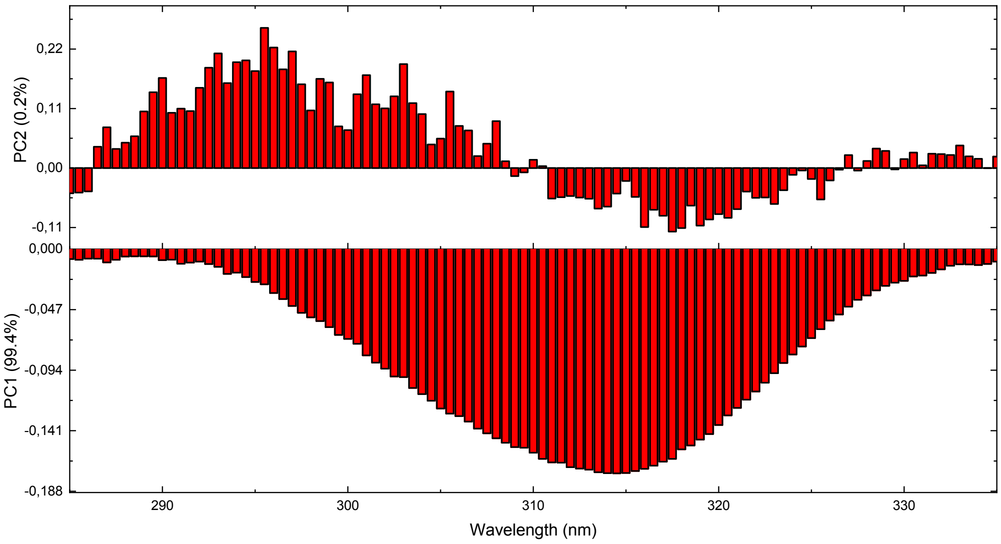

Figure S30 Loadings plot of the first two principal components along the wavelength interval 280-335 nm.

9.1 Circular Dichroism Spectra

9.1.1 Prosecco@1

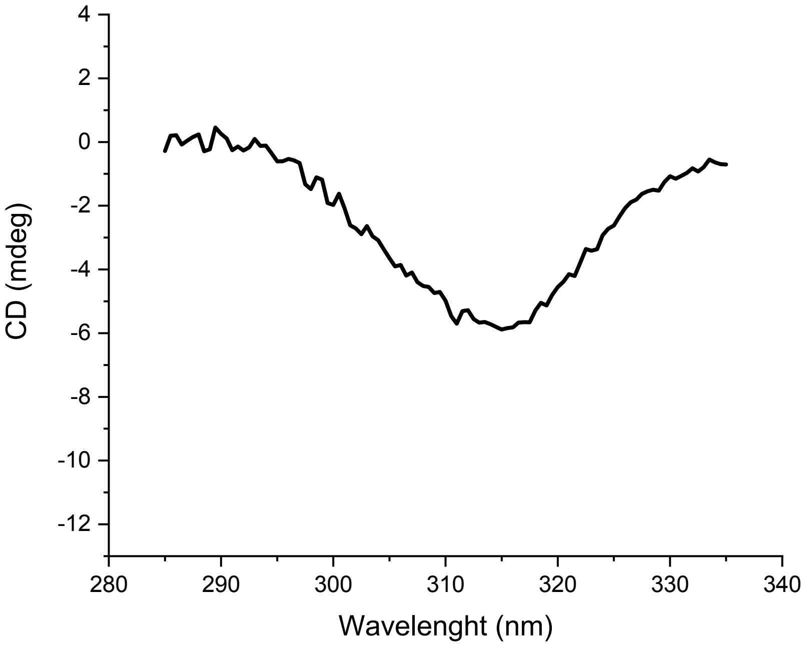

Figure S31 CD spectrum of cage **R@1** formed using Prosecco wine as complex mixture.

9.1.2 Chardonnay@1

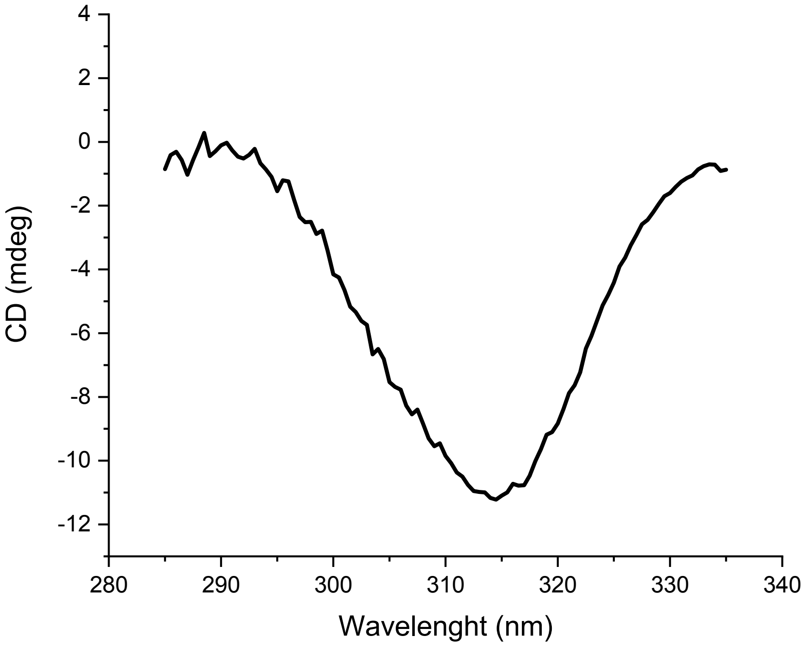

Figure S32 CD spectrum of cage **R@1** formed using Chardonnay wine as complex mixture.

9.1.3 Chianti@1

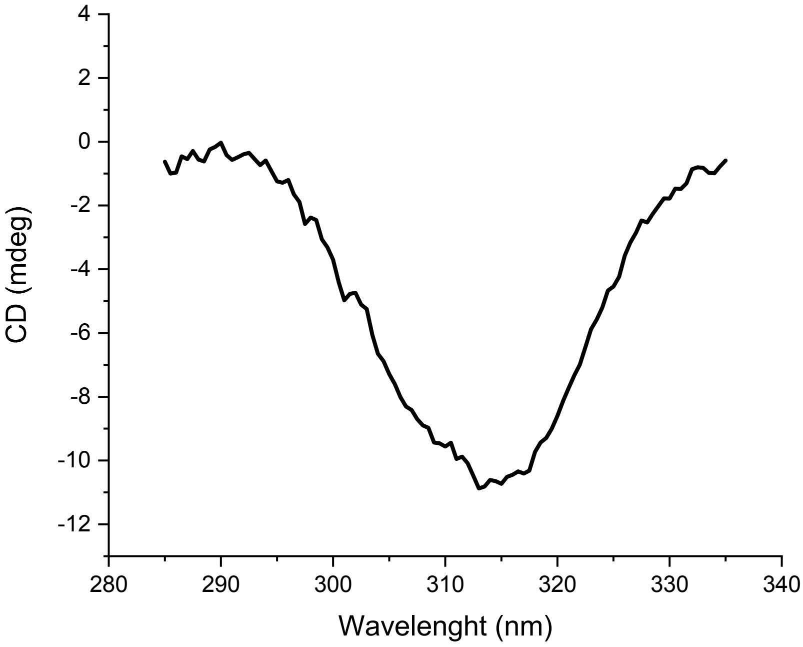

Figure S33 CD spectrum of cage **R@1** formed using Chianti wine as complex mixture.

9.1.4 Valpolicella@1

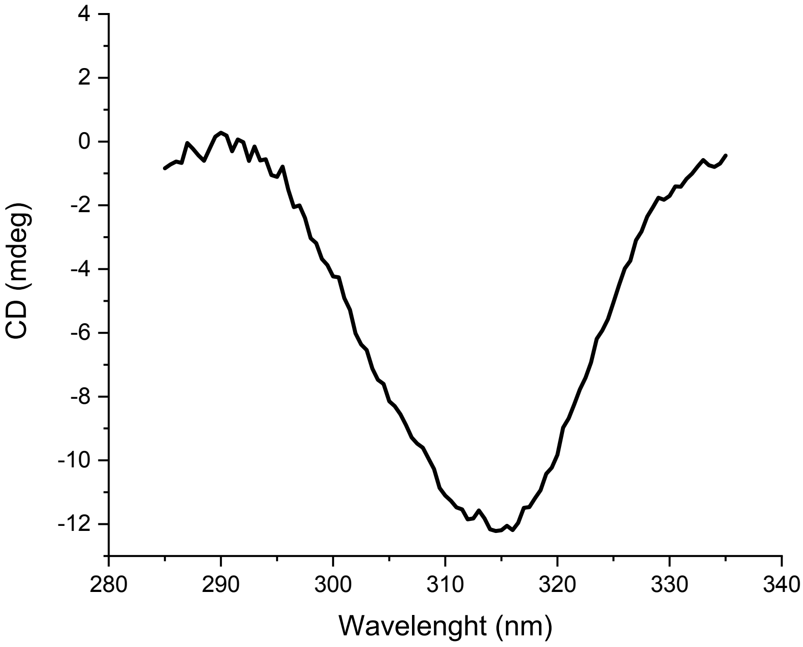

Figure S34 CD spectrum of cage **R@1** formed using Valpolicella wine as complex mixture.

9.1.5 Müller-Thurgau@1

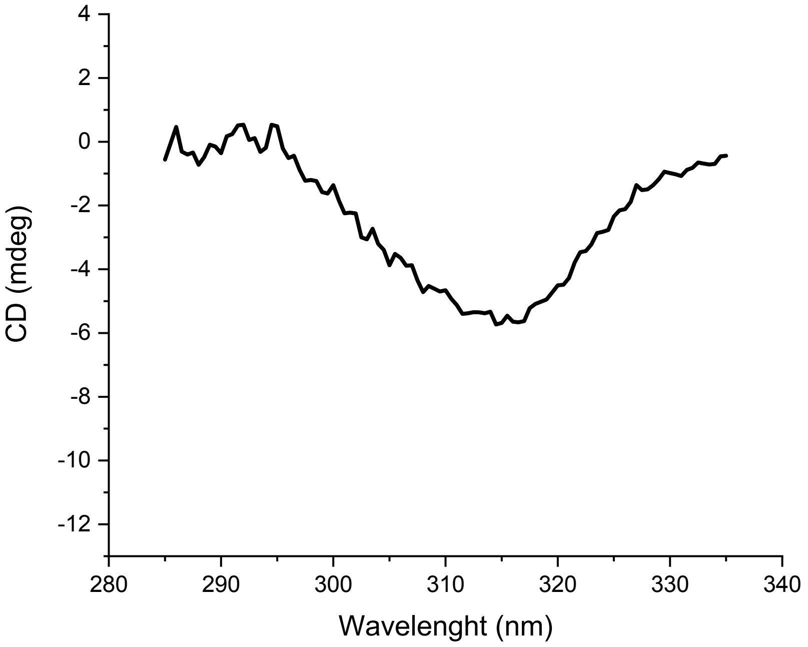

Figure S35 CD spectrum of cage **R@1** formed using Müller-Thurgau wine as complex mixture.

9.1.6 Barbera@1

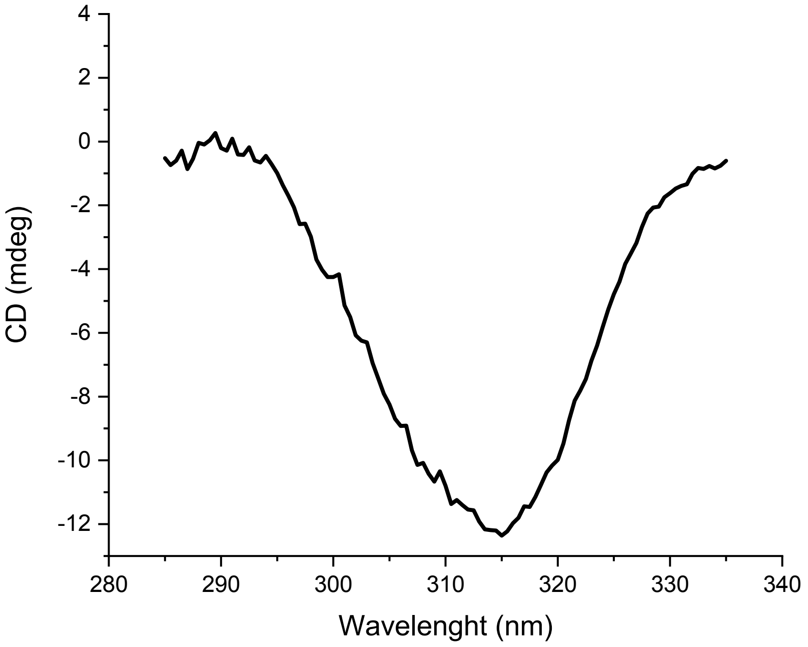

Figure S36 CD spectrum of cage **R@1** formed using Barbera wine as complex mixture.

9.1.7 Blueberry juice-1@1

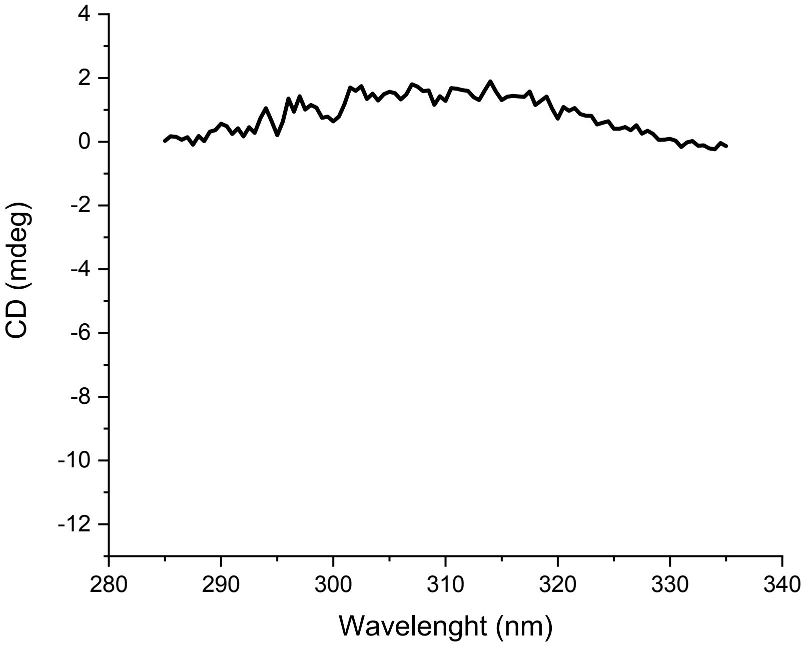

Figure S37 CD spectrum of cage **R@1** formed using Blueberry juice as complex mixture.

9.1.8 Blueberry juice-2@1

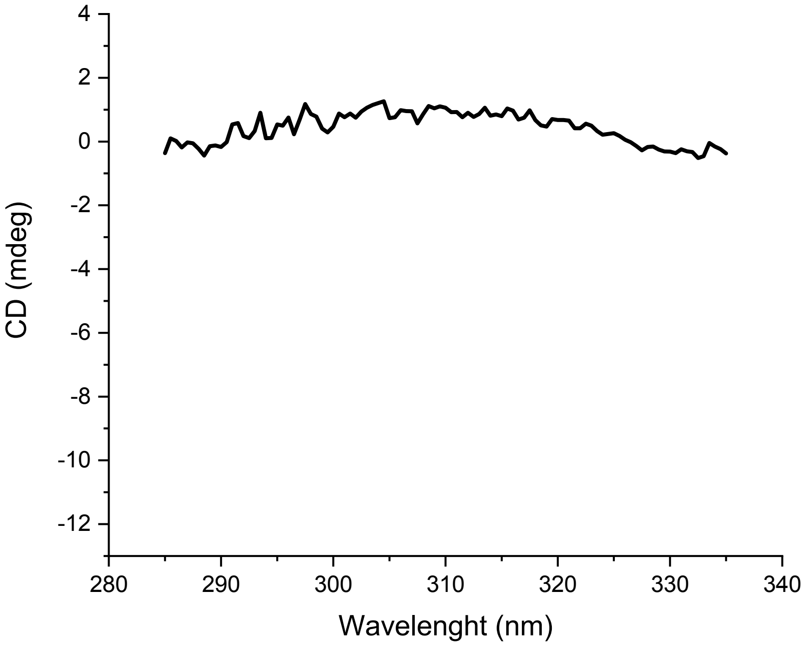

Figure S38 CD spectrum of cage **R@1** formed using Blueberry juice as complex mixture.

9.1.9 Blueberry juice-3@1

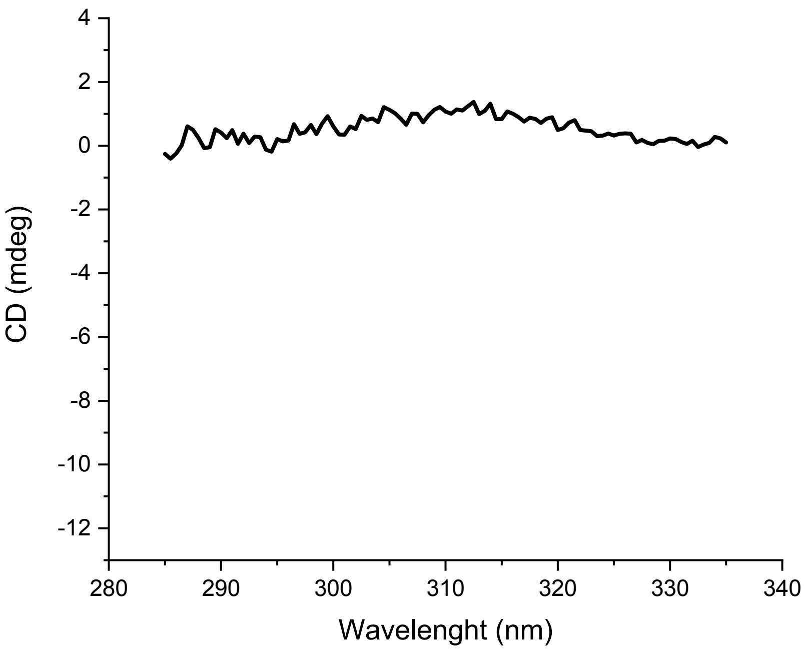

Figure S39 CD spectrum of cage **R@1** formed using Blueberry juice as complex mixture.

9.1.10 Apple juice-1@1

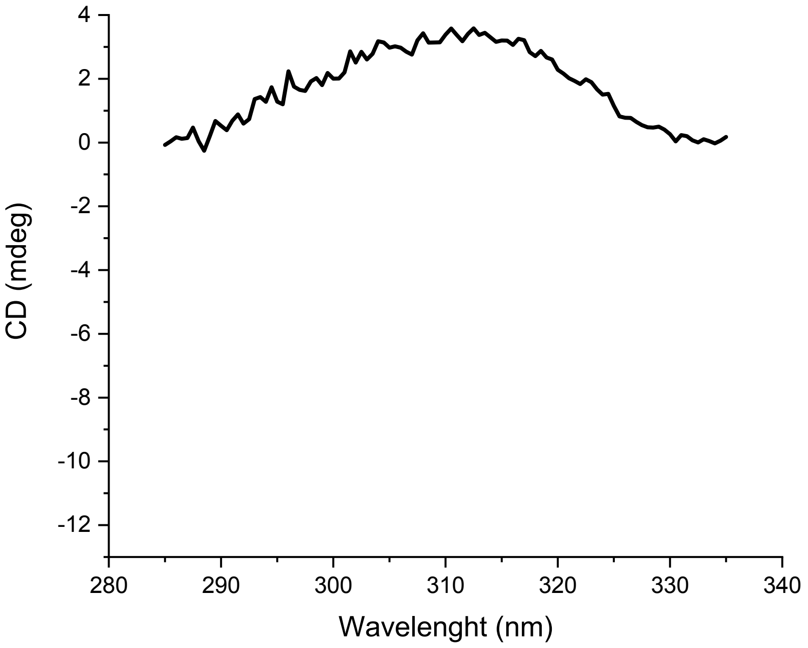

Figure S40 CD spectrum of cage **R@1** formed using Apple juice as complex mixture.

9.1.11 Apple juice-2@1

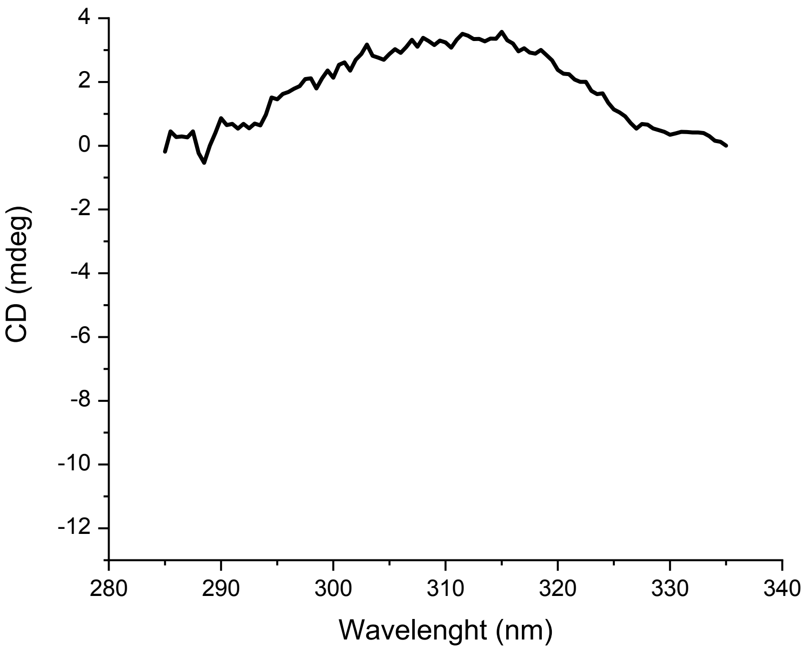

Figure S41 CD spectrum of cage **R@1** formed using Apple juice as complex mixture.

9.1.12 Apple juice-3@1

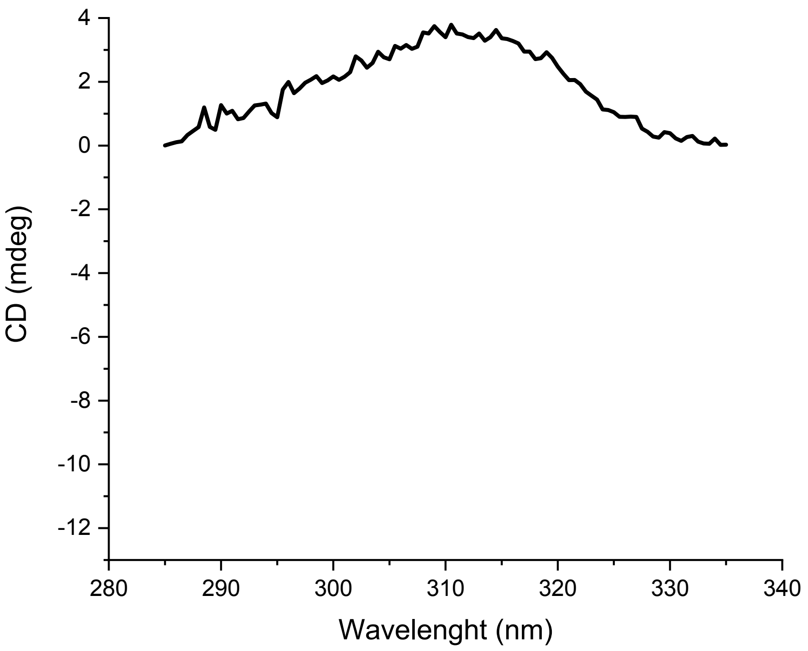

Figure S42 CD spectrum of cage **R@1** formed using Apple juice as complex mixture.

9.1.13 Pear juice-1@1

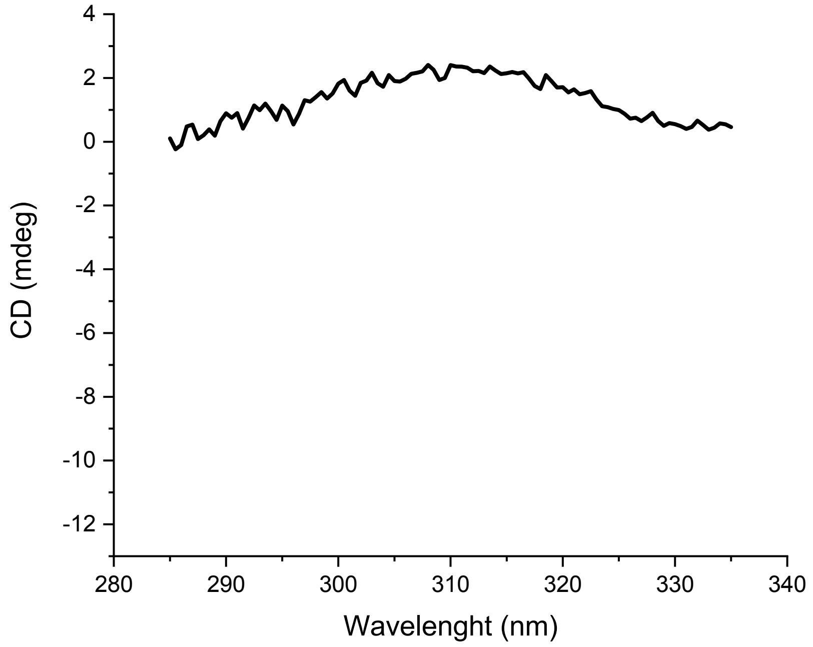

Figure S43 CD spectrum of cage **R@1** formed using Pear juice as complex mixture.

9.1.14 Pear juice-2@1

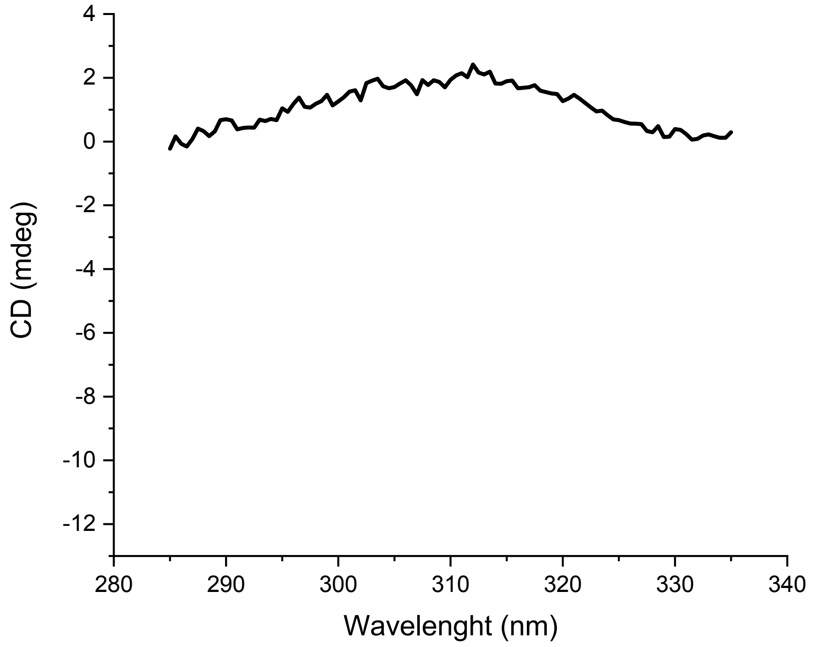

Figure S44 CD spectrum of cage **R@1** formed using Pear juice as complex mixture.

9.1.15 Pear juice-3@1

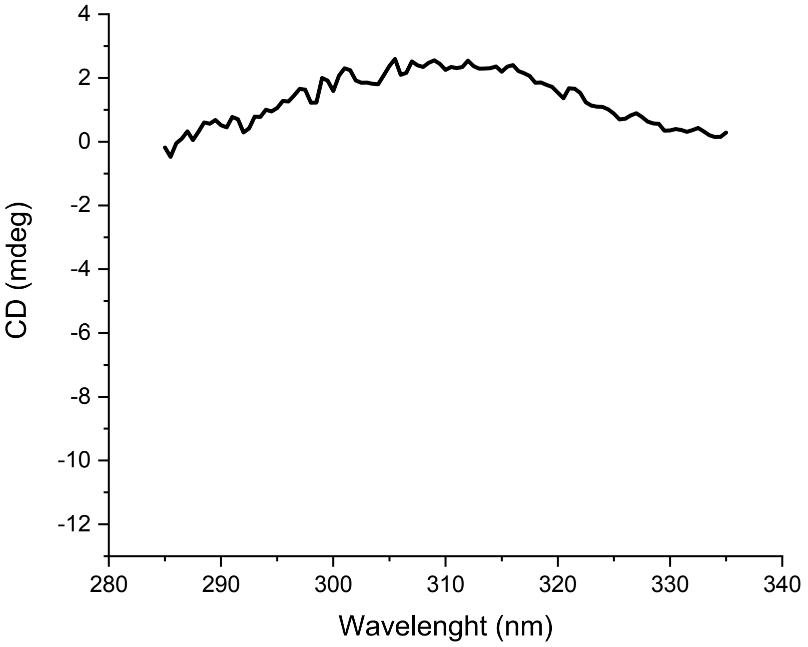

Figure S45 CD spectrum of cage **R@1** formed using Pear juice as complex mixture.

9.1.16 Orange Juice@1

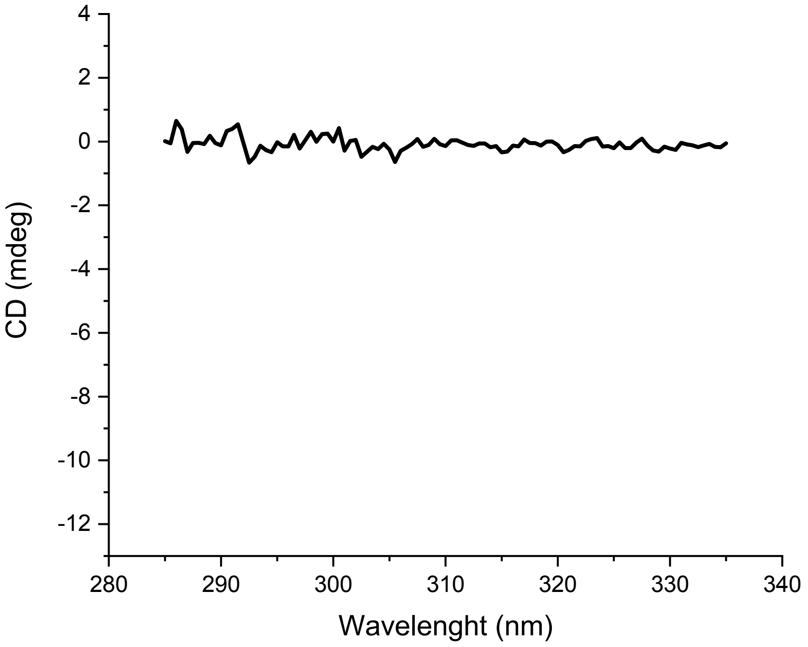

Figure S46 CD spectrum of cage **R@1** formed using Orange squeezed juice as complex mixture.

9.1.17 Lemon Juice@1

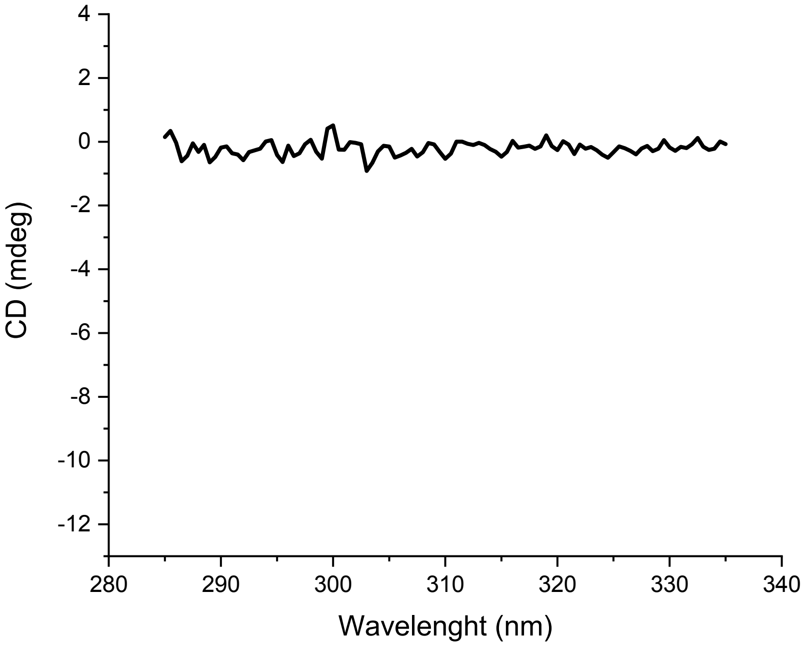

Figure S47 CD spectrum of cage **R@1** formed using Lemon squeezed juice as complex mixture.

10 Quantification of Dicarboxylic acids Content in the Complex Mixtures for the PCA Analysis

In order to determine the concertation of Tartaric and Malic in the complex mixtures, the concentration of cages **L-Tar@1** and **L-Mal@1** has been determined using p-xylene as internal standard.

Complex Mixture	L-Tartaric Acid (g/L)	L-Malic Acid (g/L)
**Prosecco**	1.3	1.6
**Chianti**	2.4	n.d
**Chardonnay**	1.5	1.6
**Valpolicella**	2.0	0.3
**Müller-Thurgau**	1.5	1.7
**Barbera**	2.5	n.d
**Blueberry Juice-1**	n.d	1.4
**Blueberry Juice-2**	n.d	2.5
**Blueberry Juice-3**	n.d	2.1
**Apple Juice-1**	n.d	4.7
**Apple Juice-2**	n.d	3.9
**Apple Juice-3**	n.d	4.0
**Pear Juice-1**	n.d	2.1
**Pear Juice-2**	n.d	2.7
**Pear Juice-3**	n.d	2.1
**Lemon Juice**	n.d	n.d
**Orange Juice**	n.d	n.d

Table S7. Quantification of **L-Tartaric** and **L-Malic** acids content in the complex mixtures.

10.1 Prosecco@1

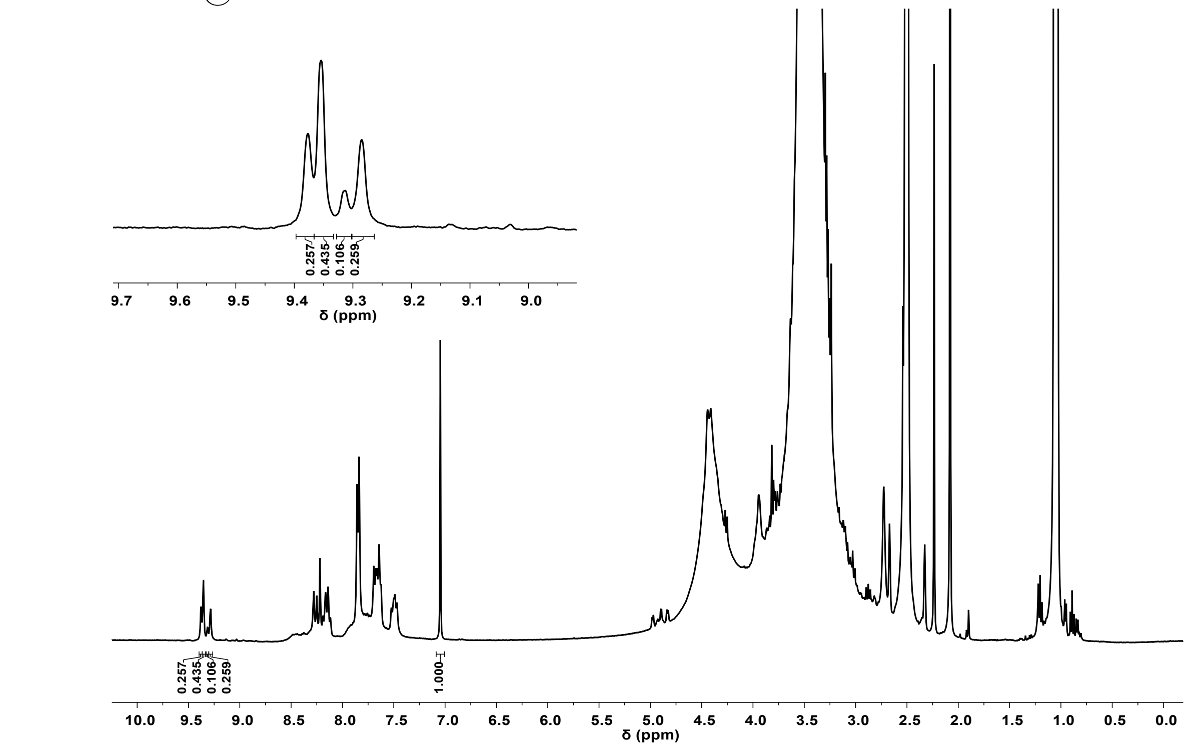

Figure S48^{1}H-NMR spectrum (400 MHz, 301 K, DMSO-d_6) of the system formed adding Prosecco wine without pre-treatment to the DMSO-d_6 solution containing complex **2** and ethylenediamine.

10.2 Chianti@1

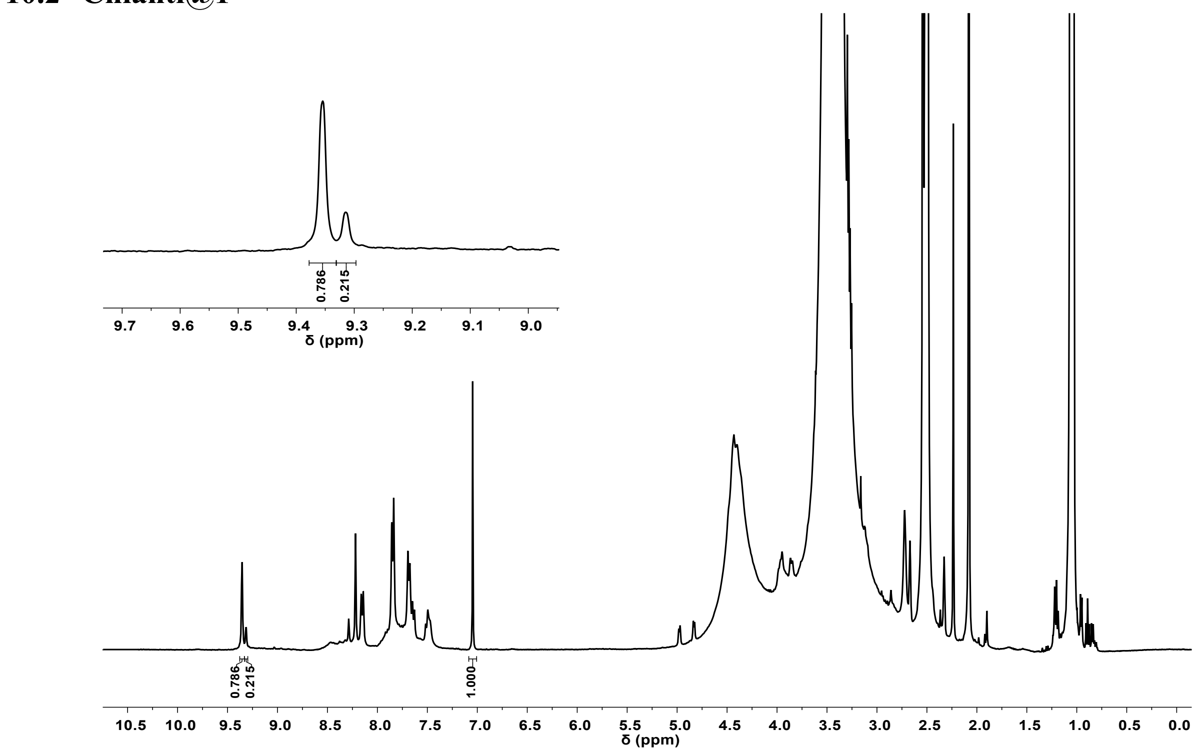

Figure S49 ^{1}H-NMR spectrum (400 MHz, 301 K, DMSO-d_6) of the system formed adding Chianti wine without pre-treatment to the DMSO-d_6 solution containing complex **2** and ethylenediamine.

10.3 Chardonnay@1

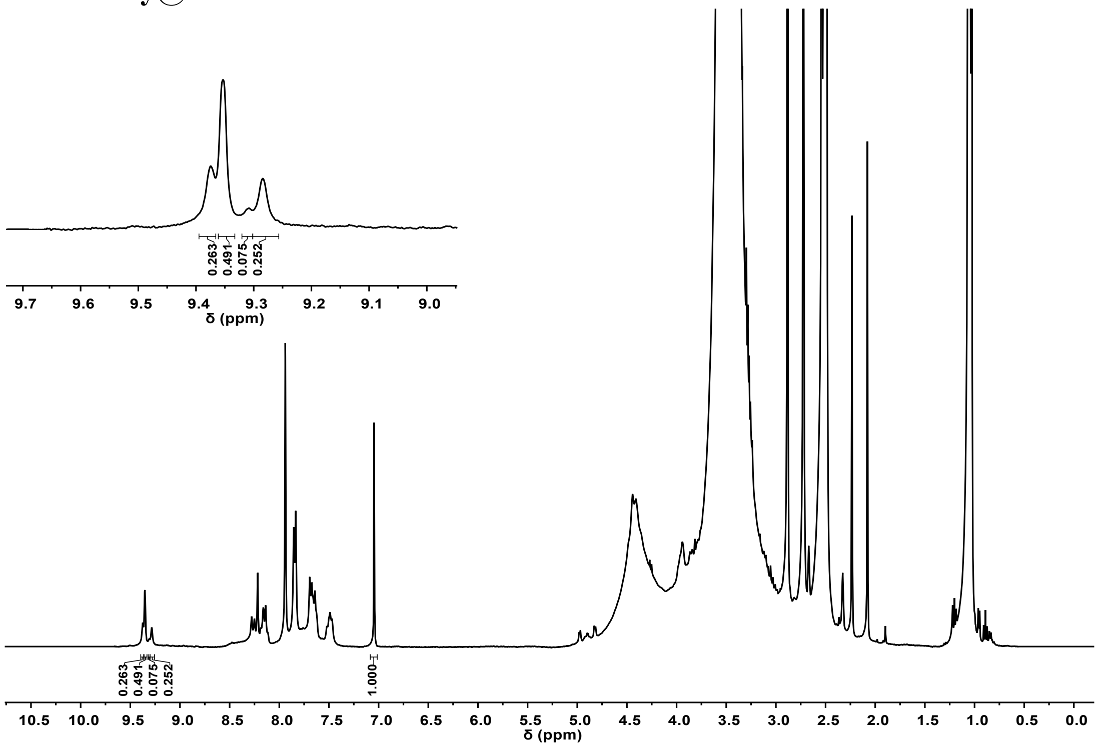

Figure S50 ^{1}H-NMR spectrum (400 MHz, 301 K, DMSO-d_6) of the system formed adding Chardonnay wine without pre-treatment to the DMSO-d_6 solution containing complex **2** and ethylenediamine.

10.4 Valpolicella@1

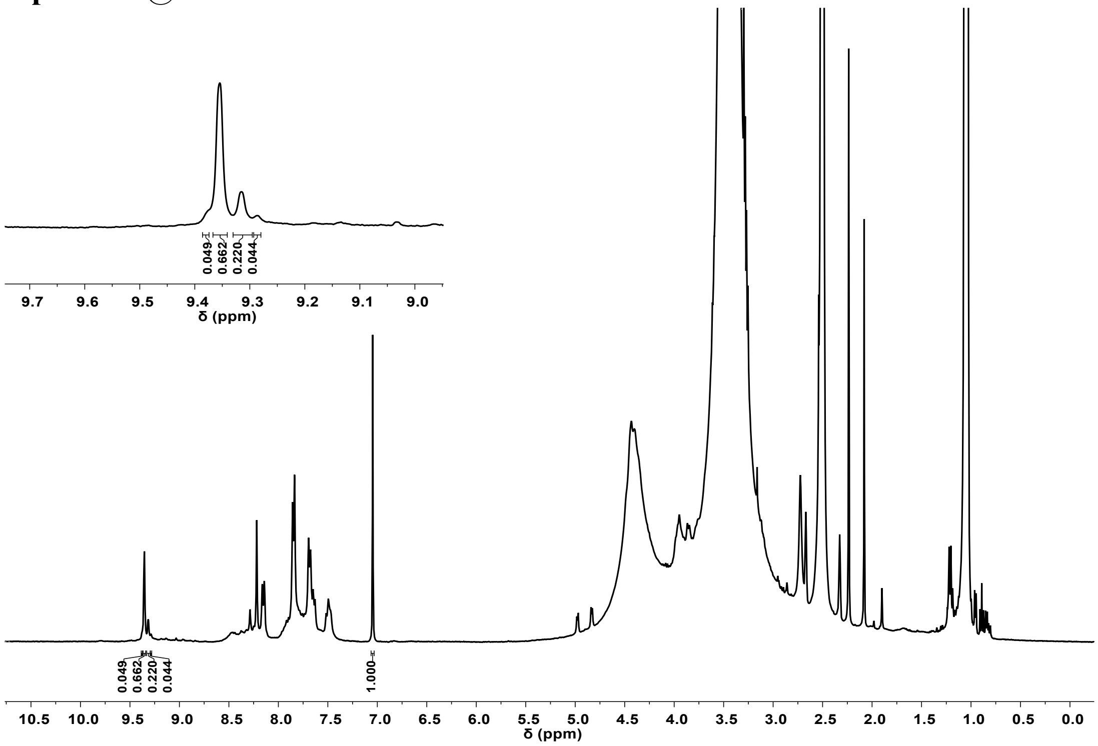

Figure S51 ^{1}H-NMR spectrum (400 MHz, 301 K, DMSO-d_6) of the system formed adding Valpolicella wine without pre-treatment to the DMSO-d_6 solution containing complex **2** and ethylenediamine.

10.5 Müller-Thurgau@1

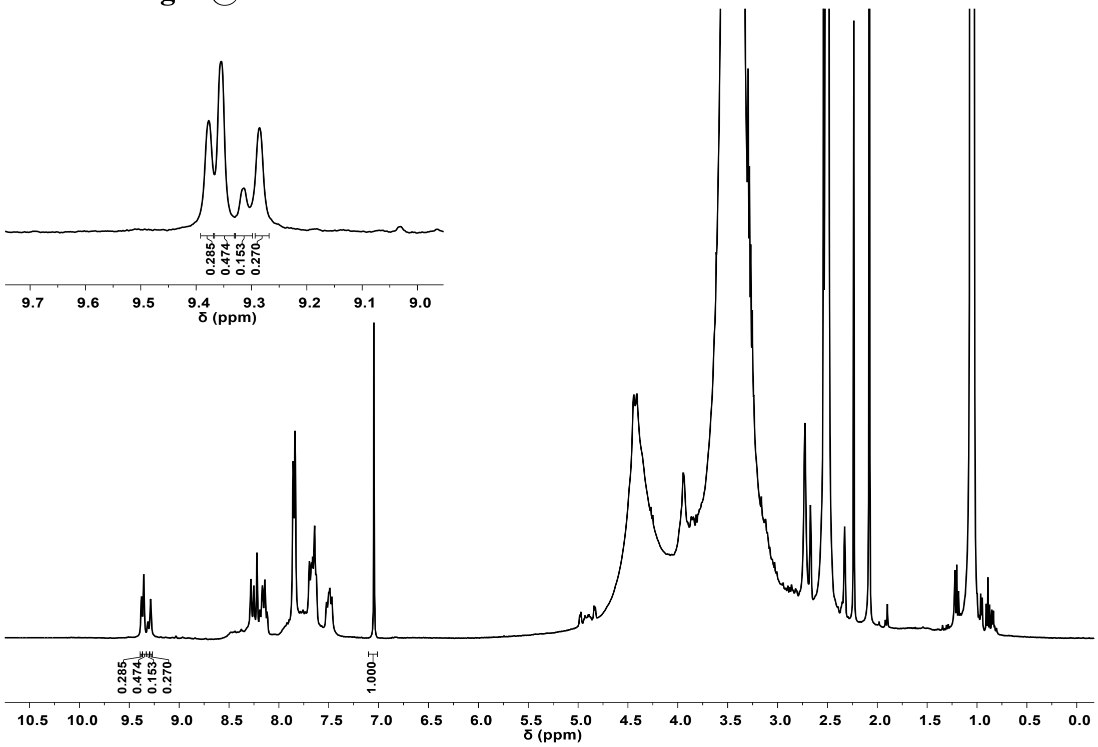

Figure S52 ^{1}H-NMR spectrum (400 MHz, 301 K, DMSO-d_6) of the system formed adding Müller-Thurgau wine without pre-treatment to the DMSO-d_6 solution containing complex **2** and ethylenediamine.

10.6 Barbera@1

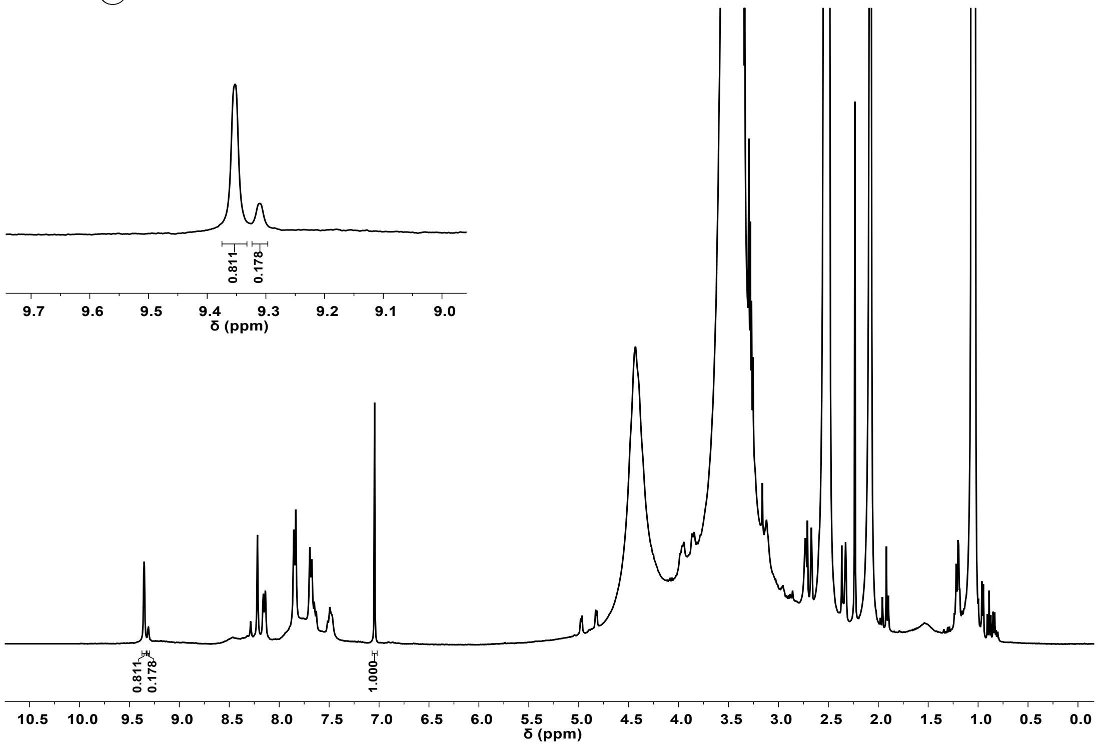

Figure S53 ^{1}H-NMR spectrum (400 MHz, 301 K, DMSO-d_6) of the system formed adding Barbera wine without pre-treatment to the DMSO-d_6 solution containing complex **2** and ethylenediamine.

10.7 Blueberry juice-1@1

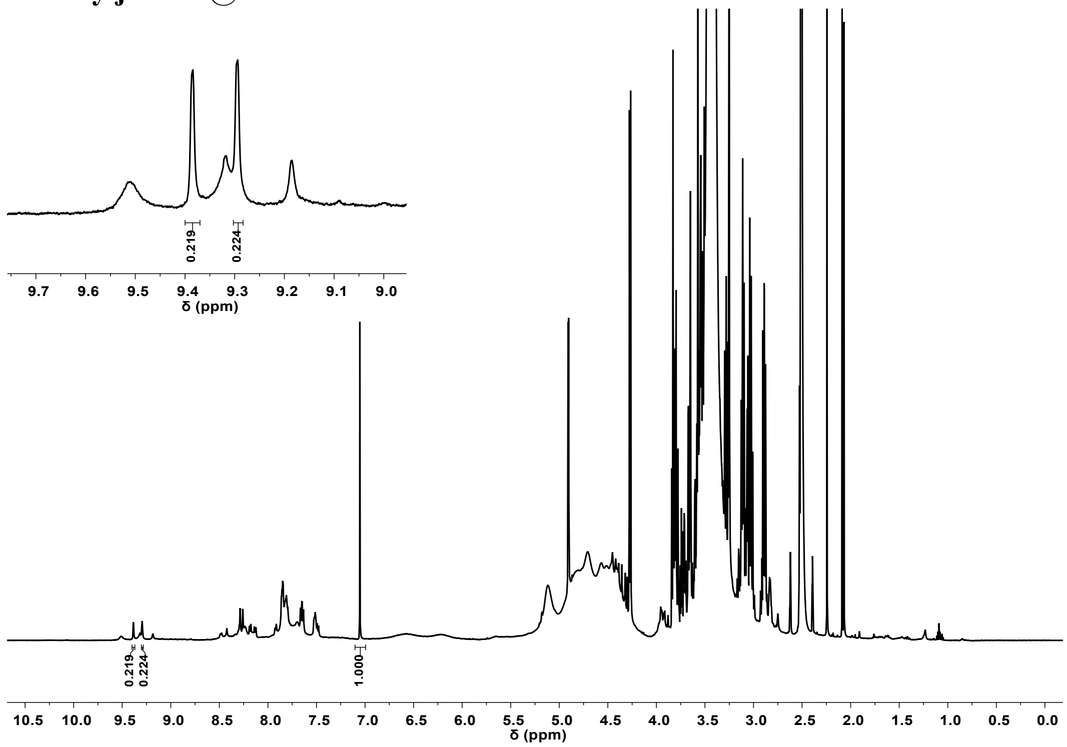

Figure S54 ^{1}H-NMR spectrum (600 MHz, 301 K, DMSO-d_6) of the system formed adding Blueberry juice without pre-treatment to the DMSO-d_6 solution containing complex **2** and ethylenediamine.

10.8 Blueberry juice-2@1

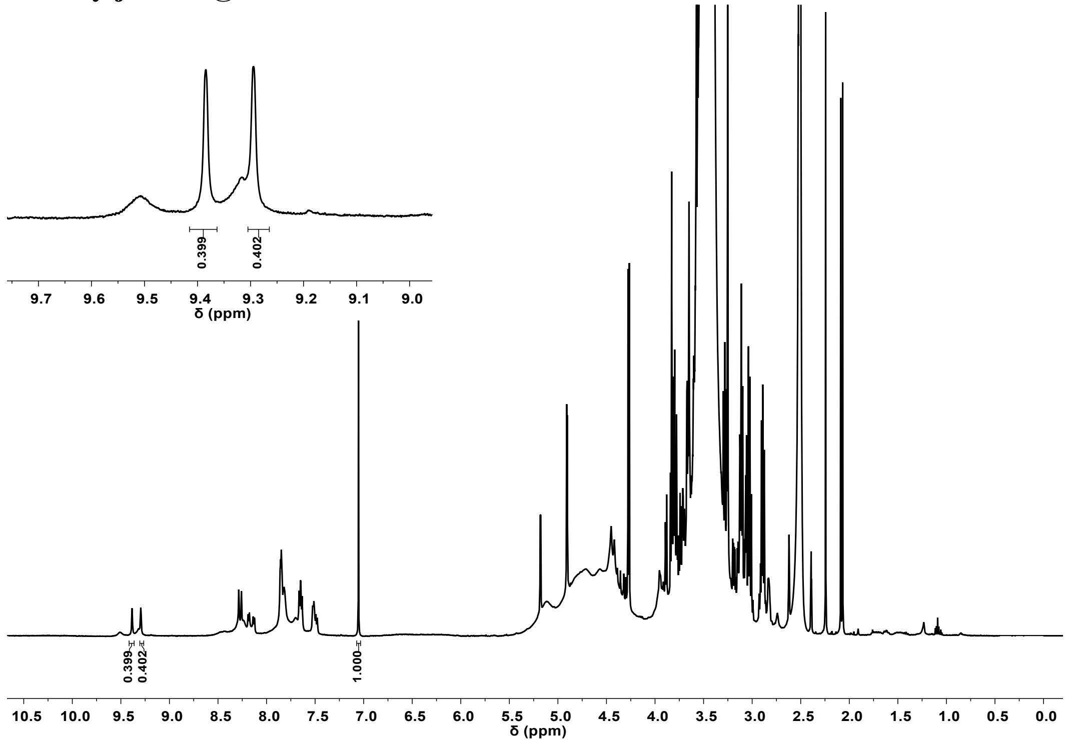

Figure S55 ^{1}H-NMR spectrum (400 MHz, 301 K, DMSO-d_6) of the system formed adding Blueberry juice without pre-treatment to the DMSO-d_6 solution containing complex **2** and ethylenediamine.

10.9 Blueberry juice-3@1

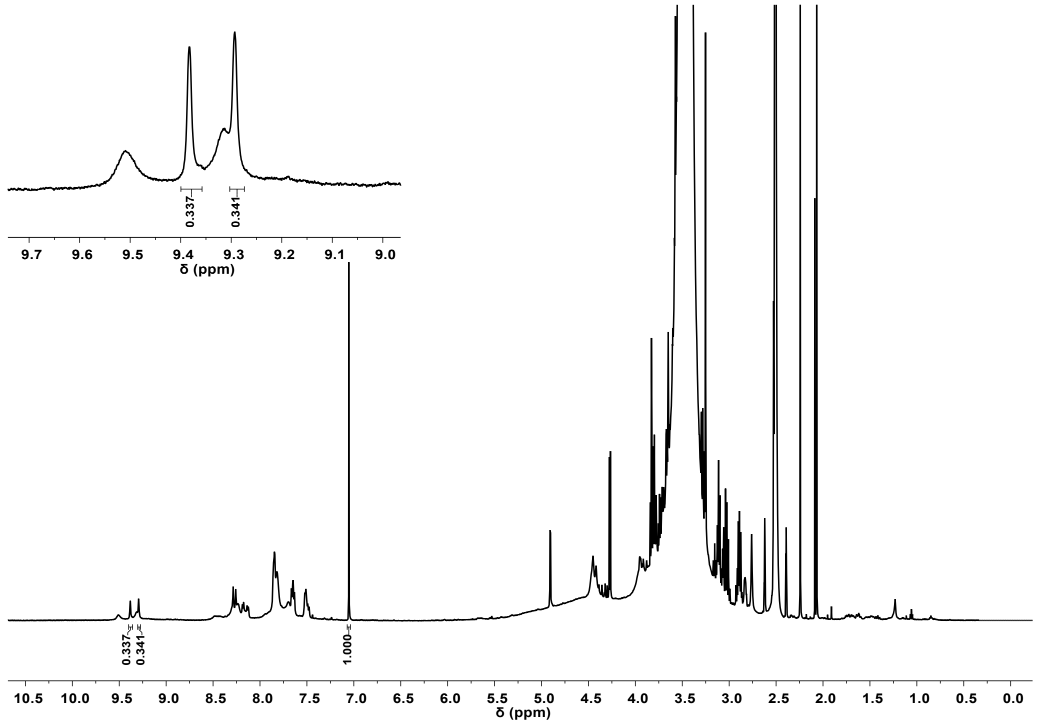

Figure S56 ^{1}H-NMR spectrum (600 MHz, 301 K, DMSO-d_6) of the system formed adding Blueberry juice without pre-treatment to the DMSO-d_6 solution containing complex **2** and ethylenediamine.

10.10 Apple Juice-1@1

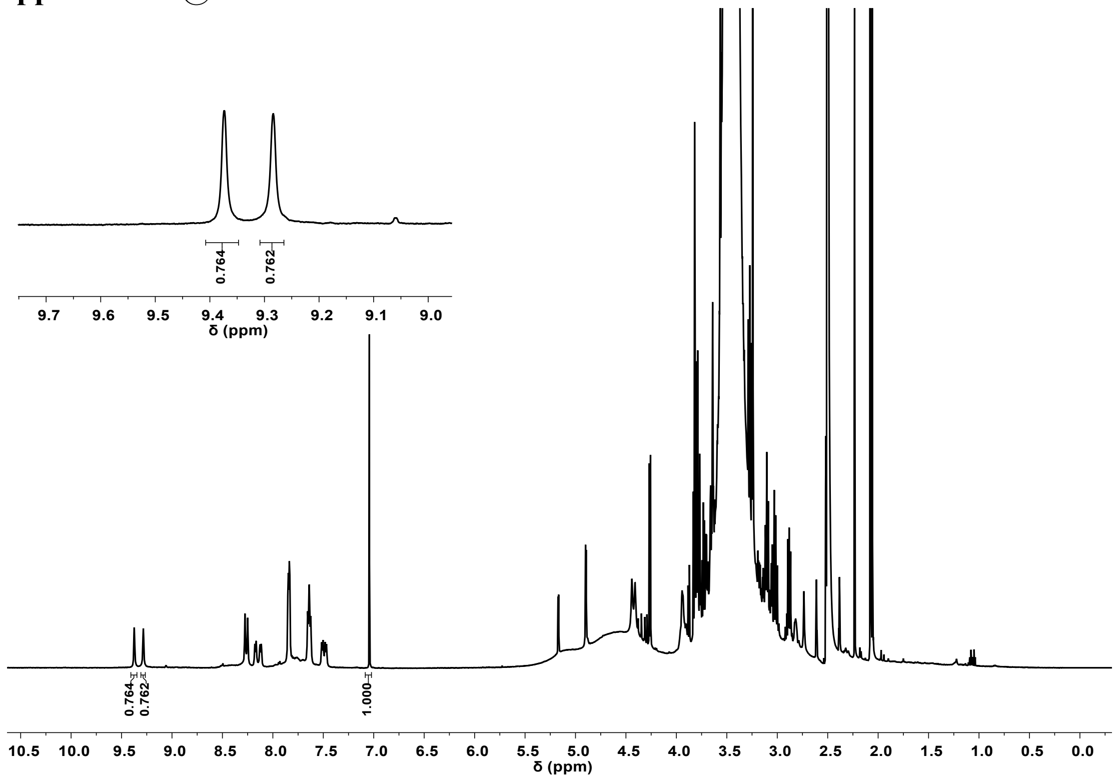

Figure S57 ^{1}H-NMR spectrum (400 MHz, 301 K, DMSO-d_6) of the system formed adding Apple juice without pre-treatment to the DMSO-d_6 solution containing complex **2** and ethylenediamine.

10.11 Apple Juice-2@1

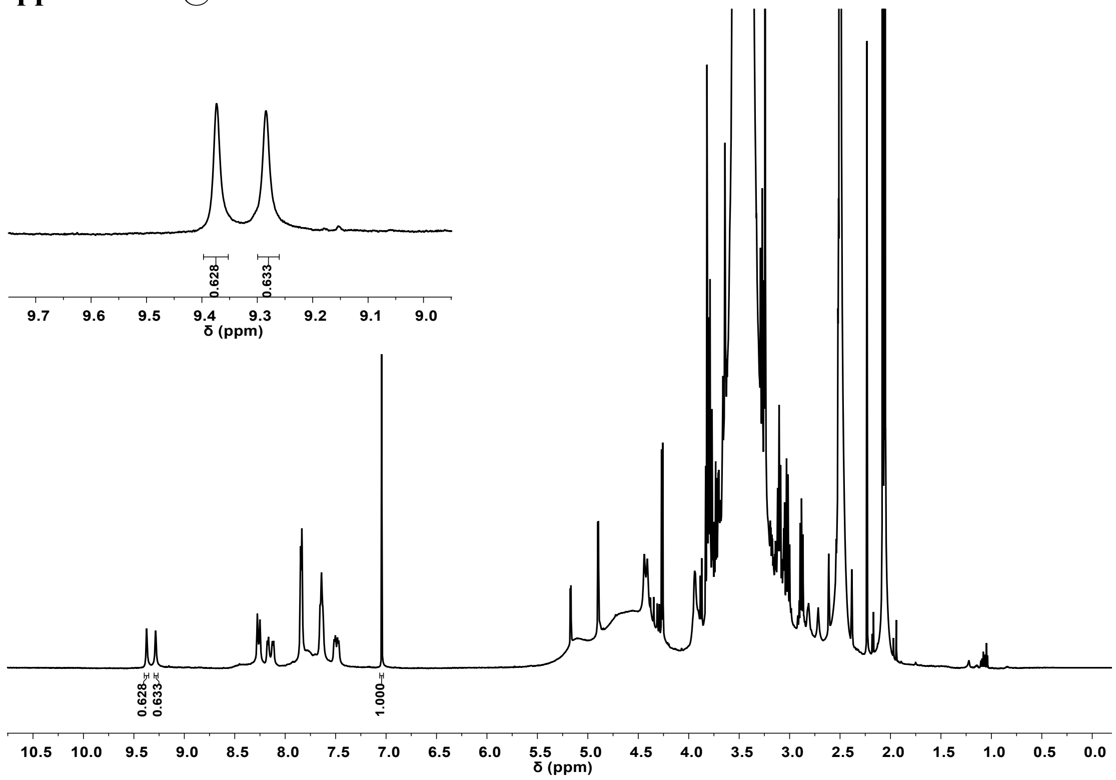

Figure S58 ^{1}H-NMR spectrum (600 MHz, 301 K, DMSO-d_6) of the system formed adding Apple juice without pre-treatment to the DMSO-d_6 solution containing complex **2** and ethylenediamine.

10.12 Apple Juice-3@1

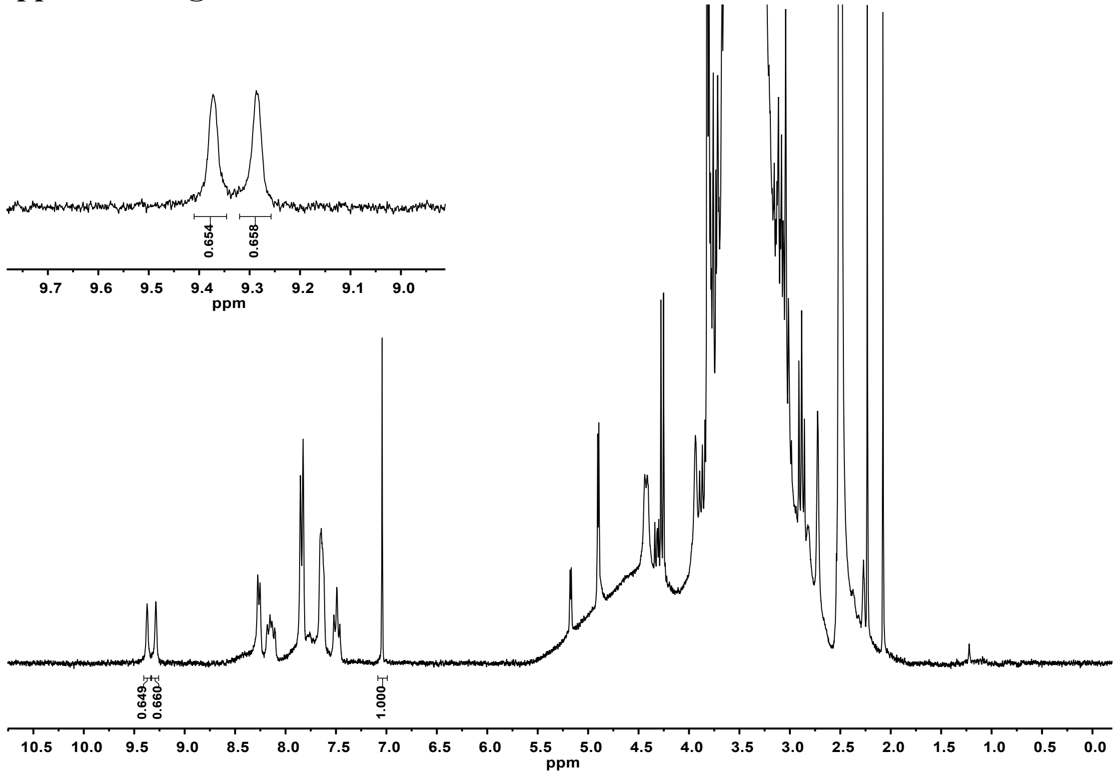

Figure S59 ^{1}H-NMR spectrum (300 MHz, 301 K, DMSO-d_6) of the system formed adding Apple juice without pre-treatment to the DMSO-d_6 solution containing complex **2** and ethylenediamine.

10.13 Pear Juice-1@1

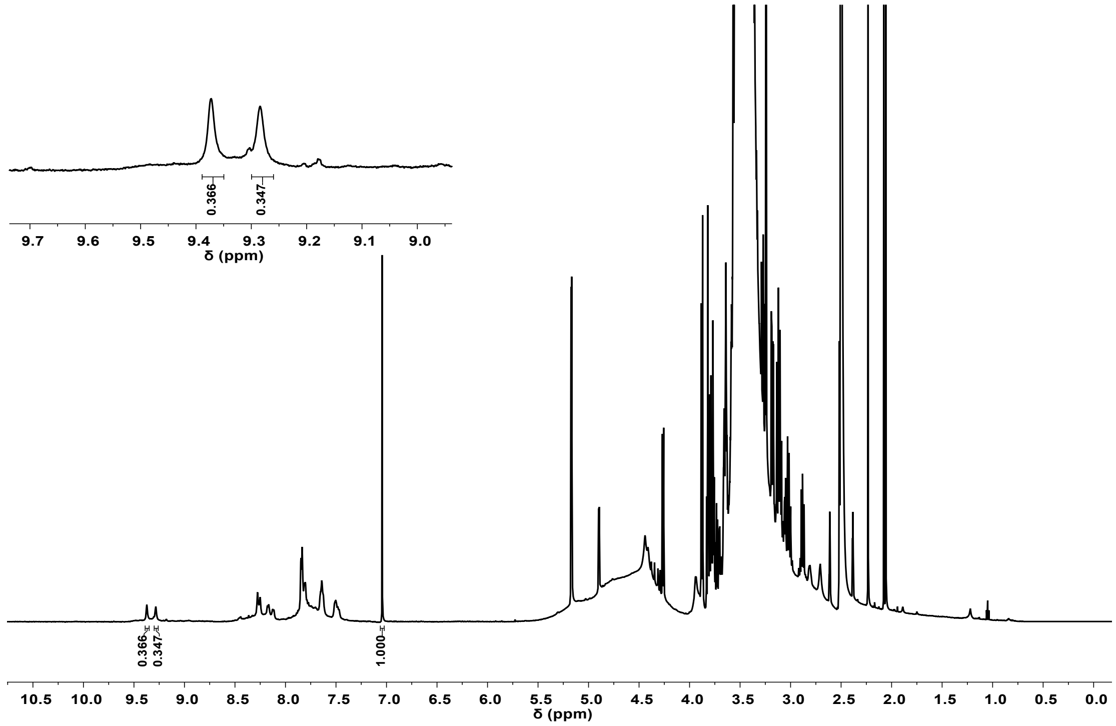

Figure S60 ^{1}H-NMR spectrum (400 MHz, 301 K, DMSO-d_6) of the system formed adding Pear juice without pre-treatment to the DMSO-d_6 solution containing complex **2** and ethylenediamine.

10.14 Pear Juice-2@1

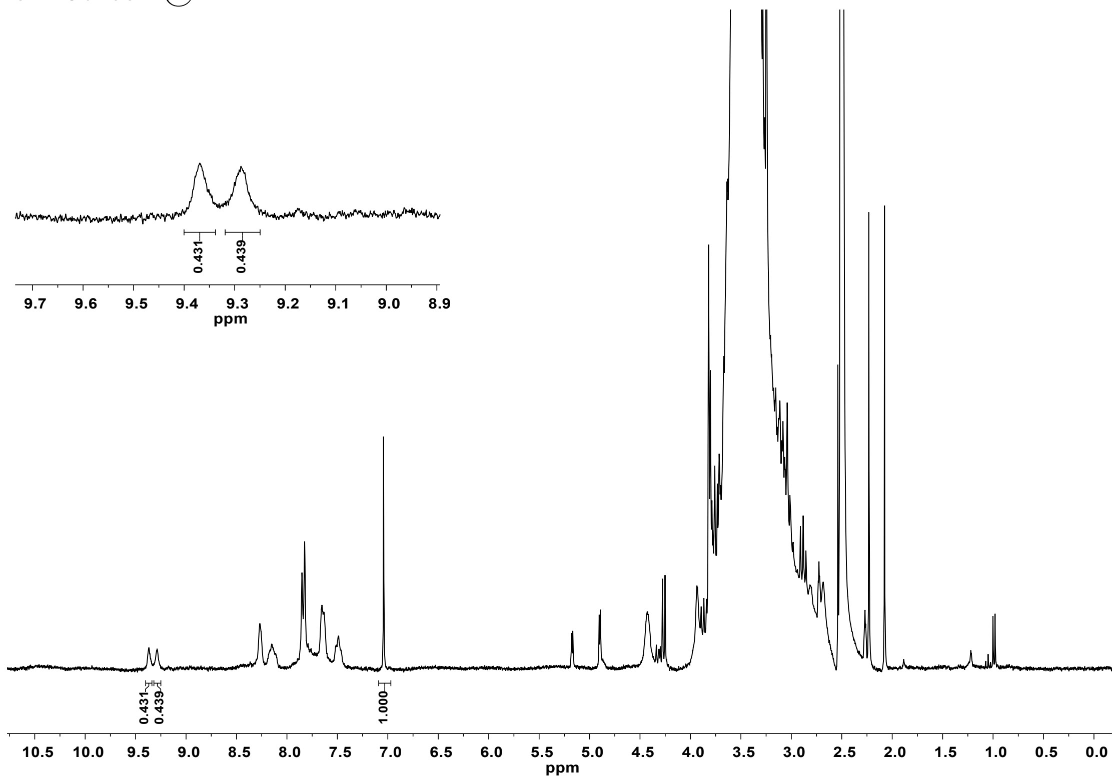

Figure S61 ^{1}H-NMR spectrum (300 MHz, 301 K, DMSO-d_6) of the system formed adding Pear juice without pre-treatment to the DMSO-d_6 solution containing complex **2** and ethylenediamine.

10.15 Pear Juice-3@1

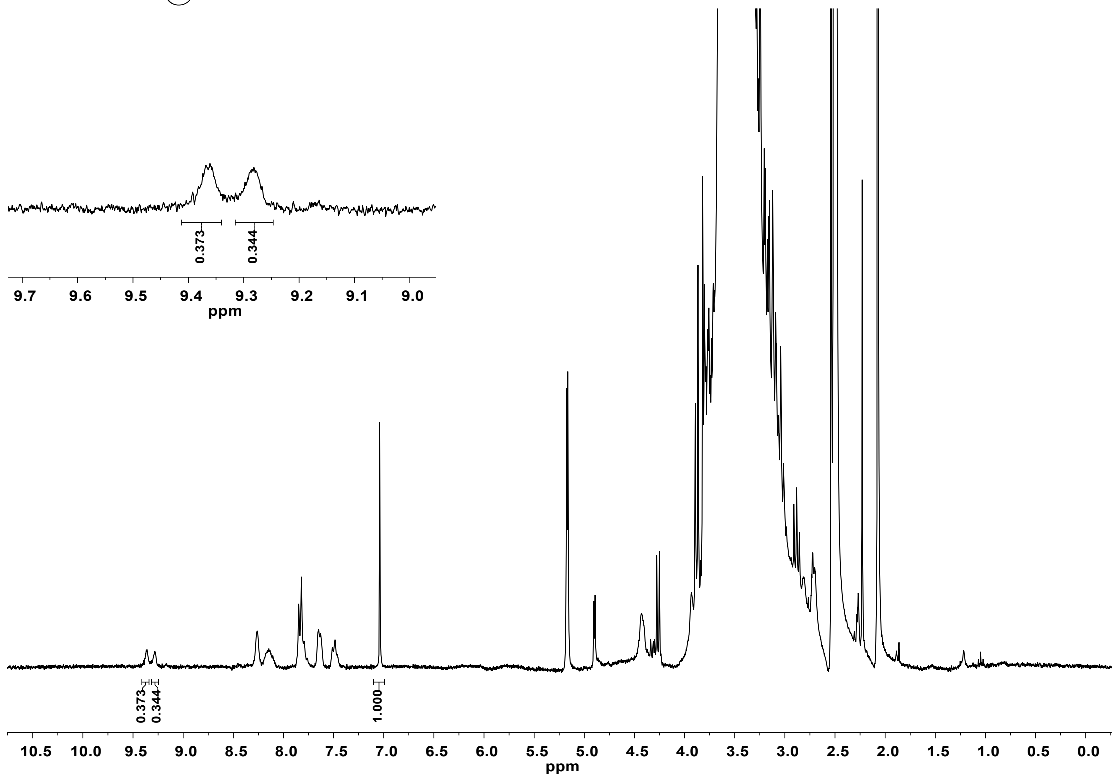

Figure S62 ^{1}H-NMR spectrum (300 MHz, 301 K, DMSO-d_6) of the system formed adding Pear juice without pre-treatment to the DMSO-d_6 solution containing complex **2** and ethylenediamine.

10.16 Lemon Juice@1

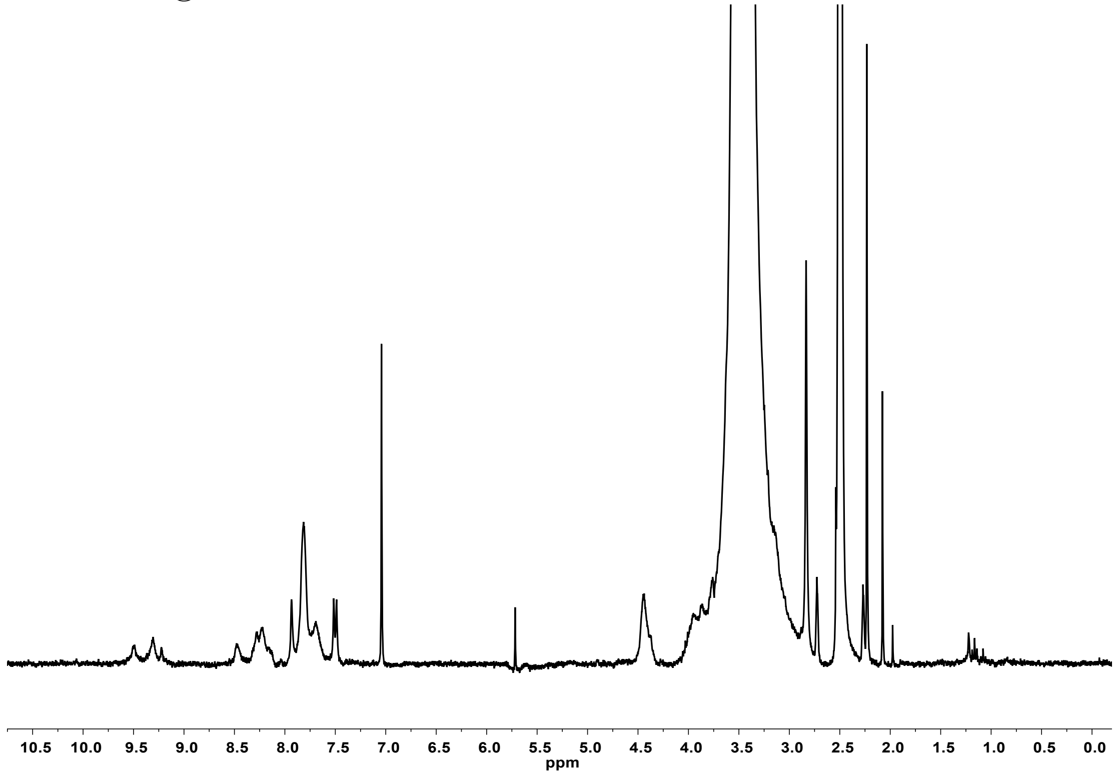

Figure S63 ^{1}H-NMR spectrum (300 MHz, 301 K, DMSO-d_6) of the system formed adding Lemon juice without pre-treatment to the DMSO-d_6 solution containing complex **2** and ethylenediamine.

10.17 Orange Juice@1

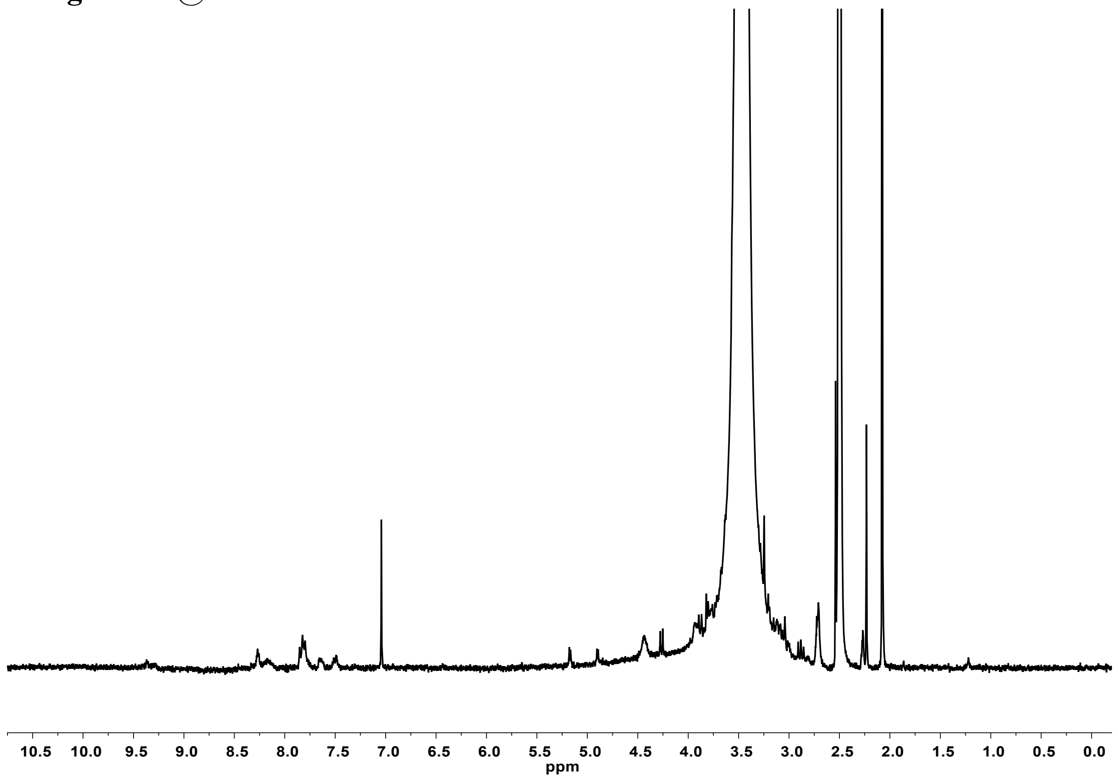

Figure S64 ^{1}H-NMR spectrum (300 MHz, 301 K, DMSO-d_6) of the system formed adding Orange juice without pre-treatment to the DMSO-d_6 solution containing complex **2** and ethylenediamine.

11 Coordinates of optimized best structures

11.1 Tar@1 entry 10

Symbol	X	Y	Z
N	-5,920551	1,889005	8,073172
C	-5,110964	-4,335355	-6,040088
C	7,222166	5,388284	-2,475688
C	-4,881288	2,394142	7,537057
C	-4,664952	-2,967232	-5,712816
C	-4,777935	-2,471885	-4,406722
C	-4,122126	-2,137106	-6,707542
C	-4,393545	-1,16717	-4,101755
H	-5,192448	-3,105726	-3,626515
C	-3,743326	-0,838966	-6,407614
H	-4,018348	-2,533147	-7,712086
C	-3,890828	-0,323806	-5,104525
H	-4,530213	-0,789723	-3,092012
H	-3,318069	-0,215796	-7,189552
C	-4,877468	2,830973	6,128453
C	-6,058146	2,810571	5,367822
C	-3,683761	3,23799	5,518222
C	-6,040854	3,193587	4,036112
H	-6,97565	2,482913	5,844901
C	-3,662132	3,610661	4,176347
H	-2,76538	3,264772	6,099474
C	-4,84221	3,59833	3,417574
H	-6,95968	3,153756	3,457336
H	-2,731176	3,947344	3,728139
C	5,767936	5,519854	-2,263164
C	5,246285	6,593108	-1,521902
C	4,883576	4,574831	-2,800266
C	3,877252	6,733331	-1,359534
H	5,937985	7,310292	-1,092858
C	3,507054	4,708409	-2,628661
H	5,275368	3,73859	-3,374158
C	2,983038	5,802206	-1,923278
H	3,491543	7,568692	-0,781333
H	2,836473	3,988089	-3,089539
C	-4,853184	4,036205	2,005924
C	-5,904639	4,814092	1,496552
C	-3,82189	3,708271	1,114346
C	-5,881326	5,233905	0,172942
H	-6,719887	5,113255	2,147762
H	-3,000898	3,074038	1,427661
C	-4,80643	4,874882	-0,634291
H	-6,679768	5,849954	-0,229271
C	1,5254	6,04679	-1,856568

C	0,581426	5,023913	-1,672169
C	1,023677	7,346618	-2,031899
H	0,883265	3,998952	-1,482481
C	-0,346281	7,568931	-2,056293
H	1,710182	8,171563	-2,193479
C	-1,207232	6,486937	-1,90607
H	-0,746072	8,565166	-2,219441
C	-3,616441	1,106628	-4,845403
C	-3,082741	1,58043	-3,635712
C	-3,94661	2,069763	-5,812232
H	-2,786744	0,90208	-2,841654
C	-3,775738	3,420511	-5,541973
H	-4,383584	1,759342	-6,755684
C	-3,273826	3,80356	-4,30375
H	-4,059583	4,173857	-6,270438
H	7,542149	4,557248	-3,123498
H	-3,930336	2,506466	8,080874
N	-0,74698	5,236068	-1,703791
N	-3,791591	4,119328	-0,164593
N	-2,9195	2,89133	-3,372549
N	-3,335781	5,44772	-2,497737
Zn	-2,232058	3,667685	-1,529725
H	-5,532579	-4,92014	-5,207317
N	-4,46681	-0,580056	8,805897
C	-2,348193	-6,4573	-6,325611
C	-4,427668	-1,049161	7,621771
C	8,645736	3,692757	0,092412
C	-1,605122	-6,458885	-5,051776
C	-0,542837	-5,567708	-4,849806
C	-1,967393	-7,331315	-4,01242
C	0,146915	-5,543867	-3,639552
H	-0,25081	-4,890368	-5,648571
C	-1,274391	-7,317471	-2,812627
H	-2,799558	-8,008853	-4,171383
C	-0,20347	-6,426004	-2,606284
H	0,983347	-4,862238	-3,512064
H	-1,578646	-7,988467	-2,013908
C	8,046096	2,348915	0,189481
C	8,562149	1,27633	-0,556512
C	6,939305	2,126438	1,0196
C	8,001772	0,013472	-0,447779
H	9,407743	1,459728	-1,210916
C	6,366738	0,860525	1,121054
H	6,530322	2,949265	1,6009
C	6,902246	-0,218919	0,401332
H	8,409543	-0,802352	-1,038554
H	5,527134	0,703303	1,792379
C	-3,15237	-1,429749	6,984119
C	-1,940192	-1,311979	7,68416

C	-3,129897	-1,927923	5,674405
C	-0,752872	-1,728544	7,104816
H	-1,960043	-0,90403	8,689153
C	-1,935348	-2,34279	5,087767
H	-4,059761	-2,016165	5,117797
C	-0,731664	-2,275268	5,806007
H	0,17417	-1,625509	7,661991
H	-1,94916	-2,764025	4,086532
C	6,385371	-1,592169	0,587356
C	7,263662	-2,685384	0,634532
C	5,020914	-1,863023	0,777606
C	6,777094	-3,957819	0,905249
H	8,330557	-2,528873	0,51111
H	4,270222	-1,079219	0,746222
C	5,414079	-4,130387	1,118256
H	7,450757	-4,805646	0,984177
C	0,519153	-2,866427	5,280011
C	0,885211	-2,831042	3,924395
C	1,379344	-3,55937	6,147726
H	0,292953	-2,285014	3,195241
C	2,512181	-4,192499	5,656238
H	1,132263	-3,638182	7,201287
C	2,777805	-4,138282	4,292758
H	3,168556	-4,750573	6,316866
C	0,552181	-6,468058	-1,3359
C	1,028386	-5,308796	-0,703849
C	0,833496	-7,69253	-0,708898
H	0,833005	-4,318554	-1,102572
C	1,557764	-7,719841	0,475855
H	0,510645	-8,621191	-1,168799
C	1,996563	-6,519363	1,02536
H	1,794891	-8,661414	0,961969
H	-5,33535	-1,201684	7,016642
H	8,227141	4,457436	0,765509
N	1,976097	-3,458185	3,4457
N	4,548001	-3,09752	1,03377
N	1,734098	-5,334719	0,440223
N	3,664503	-5,314873	2,343665
Zn	2,502497	-3,548959	1,404089
H	-1,954686	-5,796656	-7,114032
C	0,491597	-0,294464	-0,396198
H	1,103244	0,474826	-0,876262
C	-0,548059	0,38808	0,524865
H	-0,013399	0,953771	1,298367
C	1,426287	-1,168098	0,469643
O	1,307421	-2,450254	0,318131
O	2,205469	-0,618831	1,259404
C	-1,455592	1,350528	-0,262086
O	-2,653015	1,042982	-0,401362

O	-0,924075	2,438307	-0,710761
O	-0,157273	-1,014067	-1,423597
H	-0,640126	-1,723505	-0,965954
O	-1,347872	-0,622557	1,120847
H	-2,248651	-0,447372	0,778161
N	8,049983	6,19134	-1,933811
N	9,581721	3,948493	-0,733773
N	-5,009583	-4,802303	-7,221063
N	-3,396307	-7,1637	-6,485867
C	-4,136394	-7,026756	-7,724677
H	-3,549163	-6,556227	-8,528634
H	-4,452217	-8,022248	-8,056013
C	-5,400851	-6,176253	-7,465176
H	-5,96807	-6,61865	-6,63123
H	-6,023235	-6,203725	-8,36708
C	-5,814861	1,365814	9,42073
H	-4,931546	1,739705	9,96147
H	-6,714883	1,647823	9,9782
C	-5,746954	-0,177312	9,354203
H	-6,602992	-0,548964	8,769496
H	-5,823355	-0,567951	10,375436
C	10,052995	5,315005	-0,840338
H	9,777306	5,936247	0,02601
H	11,1448	5,301956	-0,930588
C	9,467818	5,953739	-2,122235
H	9,684821	5,296318	-2,978318
H	9,963195	6,917425	-2,284084
C	-2,708637	6,657813	-1,958974
H	-2,970882	7,555638	-2,538427
H	-3,095454	6,811768	-0,944416
C	-3,115293	5,262732	-3,935578
H	-2,094104	5,591366	-4,162029
H	-3,790686	5,881729	-4,544693
C	-4,733927	5,291086	-2,087461
H	-5,182992	4,507685	-2,709358
H	-5,322914	6,205294	-2,253271
C	4,836434	-5,480507	1,478452
H	5,610295	-6,112214	1,940028
H	4,506668	-5,996846	0,568883
C	2,768574	-6,474053	2,325951
H	3,308229	-7,419522	2,486133
H	2,064052	-6,367579	3,159628
C	3,979639	-4,838487	3,69403
H	4,314541	-5,650002	4,356921
H	4,811502	-4,128963	3,615883

11.2 Tar@1 entry 17

Symbol	X	Y	Z
N	-4,455642	-4,064749	7,660921
C	2,765285	-5,255446	-6,871237
C	-4,038377	-4,135086	6,459074
C	4,345378	7,972633	1,271768
C	3,272667	-5,033846	-5,503655
C	3,426791	-3,736019	-4,998676
C	3,566744	-6,125644	-4,67012
C	3,85942	-3,527425	-3,690674
H	3,20789	-2,882044	-5,635017
C	4,001337	-5,919602	-3,371242
H	3,434668	-7,127543	-5,064242
C	4,155601	-4,617064	-2,857212
H	3,992393	-2,512483	-3,327011
H	4,195988	-6,778253	-2,734743
C	4,744923	6,561489	1,431652
C	6,007288	6,123982	0,997521
C	3,856987	5,627034	1,981299
C	6,368556	4,792143	1,121042
H	6,684375	6,849619	0,559695
C	4,214721	4,285574	2,09808
H	2,877804	5,953292	2,323379
C	5,480982	3,8497	1,676267
H	7,341199	4,471259	0,75846
H	3,516113	3,581662	2,541297
C	-2,662801	-3,731024	6,105387
C	-1,795121	-3,235455	7,092701
C	-2,197936	-3,82579	4,786457
C	-0,494397	-2,885195	6,77221
H	-2,165354	-3,142956	8,108056
C	-0,888243	-3,473562	4,461812
H	-2,860772	-4,199571	4,009686
C	-0,00755	-3,019433	5,456222
H	0,152071	-2,489763	7,550382
H	-0,542702	-3,587783	3,438541
C	5,903902	2,441566	1,835126
C	7,232413	2,110908	2,140709
C	4,996764	1,377271	1,708813
C	7,596319	0,780732	2,311652
H	7,969999	2,894635	2,280765
H	3,948381	1,539242	1,477346
C	6,631315	-0,210752	2,176128
H	8,61649	0,512736	2,569056
C	1,424031	-2,76188	5,181118
C	1,900918	-2,260227	3,958055
C	2,389041	-3,060978	6,157374

H	1,219862	-1,987812	3,156247
C	3,737632	-2,857377	5,900208
H	2,082585	-3,487528	7,106539
C	4,121338	-2,368402	4,657076
H	4,488476	-3,09742	6,646712
C	4,637477	-4,429107	-1,472096
C	4,173715	-3,38709	-0,653258
C	5,584343	-5,300627	-0,909962
H	3,418839	-2,684722	-0,992911
C	6,007333	-5,122696	0,401464
H	6,003696	-6,103018	-1,508461
C	5,48974	-4,062868	1,138933
H	6,73928	-5,790683	0,84534
H	-4,671468	-4,491847	5,631213
H	3,358704	8,248322	1,676403
N	3,210616	-2,080641	3,702965
N	5,34801	0,088338	1,876161
N	4,598722	-3,202591	0,608384
N	5,828296	-2,404799	2,902507
Zn	3,98069	-1,528356	1,814672
H	2,56686	-4,353225	-7,471117
C	0,537903	-0,073659	-0,238777
H	0,635559	0,840364	-0,830822
C	-0,730437	0,027233	0,63242
H	-0,600167	0,861389	1,335793
C	1,762966	-0,161173	0,699441
O	2,428748	-1,272605	0,66179
O	2,013134	0,801496	1,43407
C	-2,001592	0,290541	-0,190808
O	-2,936063	-0,526225	-0,094131
O	-2,044288	1,367736	-0,898629
O	0,431578	-1,150267	-1,147295
H	0,350331	-1,940762	-0,586173
O	-0,875665	-1,199932	1,335619
H	-1,790109	-1,485628	1,129681
N	5,097873	8,807685	0,671718
N	2,545273	-6,431776	-7,307709
C	1,929733	-6,600401	-8,606832
H	1,904145	-5,673747	-9,201668
H	2,492726	-7,359375	-9,16214
C	0,487236	-7,123204	-8,407463
H	0,522717	-7,992783	-7,733892
H	0,09738	-7,447109	-9,378311
C	-5,83394	-4,395395	7,957949
H	-6,36357	-4,856264	7,10926
H	-5,844913	-5,096357	8,800884
C	4,609079	10,149244	0,431999
H	3,716852	10,401379	1,026444
H	5,407229	10,858922	0,677322

C	6,972201	-1,672988	2,348941
H	7,875887	-1,783645	2,966714
H	7,202859	-2,110362	1,369875
C	5,861459	-3,836379	2,588325
H	6,838625	-4,289418	2,812603
H	5,12542	-4,337681	3,228785
C	5,575317	-2,118708	4,318586
H	6,227421	-2,701454	4,98571
H	5,807762	-1,061176	4,492322
N	-6,789785	-2,261638	7,235124
N	-0,359693	-6,055086	-7,908893
N	3,052153	9,584818	-1,380027
C	-5,92261	-1,360544	6,989449
C	3,137075	8,395053	-1,829909
C	-0,571431	-5,979203	-6,654156
C	-5,991812	-0,549232	5,758896
C	-4,858281	0,138227	5,304228
C	-7,170907	-0,498927	4,998685
C	-4,884515	0,831502	4,096095
C	-7,208359	0,217949	3,812416
C	-6,061823	0,881227	3,33607
C	-1,380521	-4,884833	-6,080472
C	-1,42783	-4,674308	-4,695269
C	-2,119217	-4,037066	-6,921327
C	-2,200188	-3,646768	-4,153387
C	-2,905934	-3,03148	-6,384044
C	-2,966226	-2,824704	-4,991868
C	1,928041	7,591447	-2,101221
C	0,661672	8,197781	-2,117086
C	2,023403	6,214198	-2,344399
C	-0,469553	7,448681	-2,399406
C	0,883194	5,453697	-2,602532
C	-0,377053	6,066235	-2,652011
C	-3,88661	-1,789532	-4,46777
C	-5,181085	-1,682249	-5,000019
C	-3,528816	-0,862526	-3,476528
C	-6,036606	-0,680703	-4,561955
C	-5,590076	0,211282	-3,594055
C	-1,602205	5,3226	-3,026283
C	-1,958298	4,08812	-2,460162
C	-2,488181	5,869238	-3,967436
C	-3,658961	5,198045	-4,295628
C	-3,935048	3,984726	-3,675462
C	-6,124611	1,605703	2,047793
C	-5,138806	1,44042	1,063849
C	-7,206976	2,439335	1,733028
C	-7,275075	3,042397	0,482553
C	-6,256962	2,814123	-0,437164
N	-3,098284	3,441956	-2,767341

N	-4,351894	0,11928	-3,062499
N	-5,198735	2,027193	-0,145605
C	-6,288212	3,424882	-1,8198
C	-6,47519	1,325073	-3,079789
C	-5,185995	3,197902	-3,992991
N	-5,666885	2,514008	-2,787852
Zn	-3,809042	1,669605	-1,731988
H	-5,063685	-1,167205	7,650039
H	4,101055	7,904263	-2,033761
H	-0,16896	-6,712456	-5,938373
H	-3,943876	0,113519	5,891762
H	-8,041292	-1,04069	5,353305
H	-3,996781	1,358019	3,755922
H	-8,123078	0,236015	3,225779
H	-0,860409	-5,325271	-4,034614
H	-2,065118	-4,195201	-7,993135
H	-2,241493	-3,511762	-3,075878
H	-3,471807	-2,383812	-7,048382
H	0,593759	9,26102	-1,912815
H	2,999147	5,734606	-2,336635
H	-1,44265	7,932546	-2,410129
H	0,978586	4,391355	-2,81119
H	-5,511846	-2,391186	-5,752357
H	-2,546695	-0,888418	-3,01401
H	-7,039445	-0,586903	-4,967109
H	-1,33447	3,602241	-1,716723
H	-2,249323	6,815171	-4,44339
H	-4,352496	5,609076	-5,022904
H	-4,303318	0,767337	1,218979
H	-7,990814	2,607457	2,465282
H	-8,113232	3,679315	0,216673
H	-5,711798	4,357842	-1,823243
H	-7,319741	3,686453	-2,09853
H	-7,281123	1,535336	-3,798055
H	-6,954203	1,006498	-2,146494
H	-4,953153	2,432799	-4,743099
H	-5,952748	3,852689	-4,432428
C	-6,580212	-3,111341	8,393508
H	-5,995681	-2,620882	9,186444
H	-7,555836	-3,395807	8,800865
C	4,278546	10,298345	-1,073143
H	5,140819	9,940488	-1,655616
H	4,123807	11,360137	-1,29166

11.3 Tar@1 entry 9

Symbol	X	Y	Z
C	-7,963453	-5,140878	-5,788788
C	8,772664	6,301854	-3,671539
C	0,253213	-2,982323	10,678232
N	-6,521742	-5,069647	-5,655365
N	8,604745	4,922234	-3,258892
N	0,536705	-3,586582	9,391227
C	-6,018241	-4,946558	-4,491335
C	1,258936	-2,941485	8,563577
C	7,613622	4,272292	-3,727083
C	-4,561845	-4,819876	-4,289156
C	-4,029106	-4,693712	-2,999039
C	-3,683073	-4,841034	-5,38501
C	-2,650915	-4,636765	-2,797275
C	-2,312984	-4,78822	-5,186343
C	-1,770993	-4,710949	-3,887962
C	7,296035	2,908093	-3,267051
C	6,101741	2,292346	-3,664053
C	8,164259	2,217618	-2,405534
C	5,777547	1,013998	-3,216428
C	7,850705	0,938594	-1,972702
C	6,654041	0,314247	-2,373821
C	1,520205	-3,451185	7,204233
C	1,051775	-4,714936	6,808436
C	2,230718	-2,672604	6,280995
C	1,307024	-5,189372	5,531452
C	2,478866	-3,14368	4,993594
C	2,030431	-4,414778	4,603901
C	6,372455	-1,079984	-1,969194
C	7,392335	-2,044063	-1,974014
C	5,088705	-1,51273	-1,601505
C	7,102955	-3,364994	-1,656605
C	5,793538	-3,708889	-1,33657
C	2,358755	-4,971273	3,273525
C	2,344117	-4,192466	2,106822
C	2,725477	-6,318349	3,130313
C	3,065938	-6,818486	1,880191
C	3,049422	-5,96605	0,780991
C	-0,309321	-4,836983	-3,691733
C	0,397945	-4,163881	-2,681502
C	0,425554	-5,722651	-4,496229
C	1,774763	-5,937942	-4,251121
C	2,384129	-5,26579	-3,198124
N	2,694968	-4,66874	0,899295
N	4,806474	-2,792969	-1,301025

N	1,705298	-4,378605	-2,439817
C	3,832239	-5,513825	-2,835308
C	5,405649	-5,129359	-0,994063
C	3,393562	-6,460324	-0,607805
N	4,017242	-5,386879	-1,386206
Zn	2,791977	-3,488041	-0,850707
C	-8,486143	-3,832925	-6,426133
C	-1,210826	-2,488665	10,697416
C	8,226353	7,228937	-2,559416
N	-8,356905	-2,75589	-5,464225
N	-1,340286	-1,339972	9,822409
N	6,780083	7,136081	-2,526467
C	-7,381931	-1,94629	-5,594533
C	6,231922	6,475845	-1,584288
C	-1,949746	-1,484105	8,713142
C	-7,10613	-0,903576	-4,589065
C	-7,997717	-0,673223	-3,528538
C	-5,924648	-0,153479	-4,658236
C	-7,712966	0,286371	-2,569743
C	-5,62865	0,799551	-3,686925
C	-6,522756	1,036291	-2,631521
C	-2,055309	-0,379335	7,740851
C	-1,530724	0,889711	8,037045
C	-2,677952	-0,581462	6,50188
C	-1,65187	1,928806	7,128179
C	-2,794491	0,460495	5,584194
C	-2,296042	1,736185	5,890164
C	4,769861	6,284049	-1,525415
C	3,933253	6,851502	-2,501141
C	4,190341	5,543514	-0,486457
C	2,557034	6,724863	-2,404451
C	2,806162	5,411345	-0,390007
C	1,967227	6,025444	-1,332507
C	-2,502713	2,882555	4,97846
C	-2,813362	4,153447	5,488838
C	-2,438691	2,763033	3,580887
C	-3,063557	5,212012	4,625157
C	-3,006642	4,991797	3,252757
C	0,500001	6,067531	-1,143807
C	-0,248475	5,001013	-0,619128
C	-0,200308	7,25222	-1,424726
C	-1,554578	7,350469	-1,139833
C	-2,203083	6,263876	-0,564348
C	-6,248551	2,074535	-1,615849
C	-4,957899	2,292667	-1,112597
C	-7,269803	2,901721	-1,1242
C	-6,971663	3,898098	-0,203555
C	-5,659195	4,05062	0,232377
N	-1,55839	5,102074	-0,324827

N	-2,691974	3,783879	2,743292
N	-4,66859	3,246767	-0,208955
C	-5,281687	5,125219	1,227456
C	-3,285911	6,095411	2,255121
C	-3,660214	6,336946	-0,162879
N	-3,886288	5,531629	1,041665
Zn	-2,721929	3,592173	0,590091
C	-1,532027	1,426787	-0,532934
O	-1,741934	1,870394	0,659049
O	-1,881103	2,025648	-1,568991
C	-0,859288	0,053797	-0,691432
C	0,528818	-0,017521	-0,027451
C	1,058507	-1,468444	-0,150077
O	2,207772	-1,623925	-0,724026
O	0,369061	-2,397545	0,295733
H	-8,478264	-5,304403	-4,828987
H	-8,206501	-5,970433	-6,462893
H	8,255869	6,536336	-4,614991
H	9,841827	6,504812	-3,795841
H	0,919098	-2,137498	10,914116
H	0,36794	-3,747374	11,454449
H	-6,635711	-4,93657	-3,579133
H	1,715144	-1,969831	8,810542
H	6,929631	4,698597	-4,477523
H	-4,698205	-4,668893	-2,142277
H	-4,101609	-4,916814	-6,382979
H	-2,258918	-4,587994	-1,785079
H	-1,650297	-4,803854	-6,047314
H	5,424468	2,816892	-4,333626
H	9,079774	2,707219	-2,090777
H	4,86045	0,540903	-3,556694
H	8,529044	0,420563	-1,300012
H	0,490745	-5,306544	7,524061
H	2,602546	-1,694727	6,577203
H	0,926282	-6,164101	5,238689
H	3,060855	-2,538501	4,303755
H	8,39955	-1,762761	-2,265287
H	4,251811	-0,825993	-1,526023
H	7,876841	-4,126318	-1,680208
H	2,008036	-3,162133	2,125756
H	2,77208	-6,961279	4,003611
H	3,36166	-7,855957	1,757819
H	-0,086783	-3,437839	-2,035674
H	-0,076057	-6,281494	-5,279516
H	2,341954	-6,647942	-4,845203
H	4,153214	-6,495861	-3,214044
H	4,46932	-4,763308	-3,318421
H	5,477646	-5,273292	0,090927
H	6,106506	-5,841297	-1,455478

H	4,028174	-7,356856	-0,54822
H	2,471187	-6,759229	-1,119166
H	-7,933277	-3,646253	-7,359851
H	-9,547045	-3,964898	-6,665329
H	-1,874616	-3,321432	10,416967
H	-1,453524	-2,174762	11,719104
H	8,69806	6,956178	-1,602581
H	8,502025	8,260374	-2,806219
H	-6,680062	-1,997894	-6,441485
H	6,812148	6,019979	-0,766728
H	-2,422629	-2,434065	8,418057
H	-8,904365	-1,266564	-3,47588
H	-5,231176	-0,318897	-5,479067
H	-8,405409	0,439541	-1,746282
H	-4,716037	1,38385	-3,767302
H	-1,036563	1,035448	8,991736
H	-3,089242	-1,558627	6,260779
H	-1,229724	2,900114	7,371272
H	-3,315529	0,291425	4,645783
H	4,388969	7,403034	-3,316628
H	4,826643	5,089875	0,269432
H	1,927169	7,171253	-3,169105
H	2,377898	4,874476	0,451851
H	-2,897165	4,298619	6,561135
H	-2,167226	1,827918	3,101847
H	-3,325936	6,193069	5,009427
H	0,20978	4,034409	-0,429035
H	0,332315	8,111724	-1,818216
H	-2,096971	8,272637	-1,324307
H	-4,115428	1,701416	-1,451291
H	-8,283708	2,790151	-1,495501
H	-7,744963	4,567764	0,160534
H	-5,97622	5,975156	1,149113
H	-5,381401	4,729973	2,245524
H	-2,343309	6,578867	1,973308
H	-3,917605	6,872629	2,710076
H	-3,96427	7,385701	-0,029058
H	-4,284325	5,923204	-0,963831
O	-0,681857	-0,221998	-2,073013
H	-1,136299	0,515463	-2,529165
H	-1,506887	-0,702075	-0,225746
O	1,38743	0,958622	-0,58245
H	1,40143	0,76343	-1,535871
H	0,42949	0,212641	1,037653

11.4 Mal@1 entry 15

Symbol	X	Y	Z
C	-0,447041	-3,576425	-10,356024
C	0,725859	10,91589	2,12122
C	1,566568	-7,831547	7,737706
N	0,478322	-2,871994	-9,489824
N	1,6285	9,781524	2,123649
N	2,365604	-7,234607	6,685209
C	0,661423	-3,306691	-8,306636
C	2,394126	-5,964899	6,58686
C	1,555506	8,941306	1,168516
C	1,556927	-2,613246	-7,360228
C	1,69542	-3,076249	-6,044898
C	2,292981	-1,4866	-7,763582
C	2,564259	-2,447105	-5,155526
C	3,164502	-0,865044	-6,883146
C	3,325067	-1,342182	-5,567619
C	2,392765	7,726724	1,145969
C	2,220232	6,764485	0,142024
C	3,371468	7,512233	2,130684
C	3,015598	5,621353	0,105719
C	4,171563	6,381462	2,089726
C	4,015912	5,422111	1,070118
C	3,129386	-5,293203	5,498098
C	3,917753	-6,03328	4,601353
C	3,044693	-3,903777	5,33799
C	4,613984	-5,394932	3,586912
C	3,733324	-3,262235	4,310978
C	4,537252	-3,997832	3,426831
C	4,943453	4,273427	0,98465
C	6,301895	4,422146	1,30193
C	4,528217	3,00598	0,544889
C	7,174947	3,352756	1,144721
C	6,685859	2,139805	0,67381
C	5,335563	-3,327873	2,377642
C	4,839098	-2,246385	1,634527
C	6,646723	-3,74095	2,091353
C	7,392998	-3,077434	1,125007
C	6,823626	-2,000881	0,451848
C	4,339719	-0,734601	-4,678959
C	4,136448	-0,558595	-3,302246
C	5,589827	-0,341721	-5,184743
C	6,564993	0,159063	-4,331515
C	6,285228	0,269245	-2,973068
N	5,563812	-1,596314	0,707943
N	5,374798	1,969238	0,390768
N	5,079321	-0,074192	-2,475721

C	7,310489	0,784566	-1,984222
C	7,598422	0,954708	0,451117
C	7,572664	-1,24418	-0,625023
N	7,119659	0,148757	-0,676442
Zn	4,815065	0,086888	-0,384081
C	-1,72704	-2,732606	-10,541199
C	0,27763	-8,427072	7,129098
C	-0,402117	10,674302	3,150747
N	-2,503916	-2,75867	-9,317149
N	-0,626521	-7,355342	6,760432
N	-1,32296	9,68014	2,635152
C	-2,521489	-1,716558	-8,584813
C	-1,260719	8,493732	3,096458
C	-0,788707	-7,088613	5,525071
C	-3,236395	-1,690909	-7,294618
C	-4,024833	-2,78	-6,887579
C	-3,13291	-0,576668	-6,451337
C	-4,703492	-2,742411	-5,679288
C	-3,80313	-0,54279	-5,230617
C	-4,607686	-1,621423	-4,832496
C	-1,660184	-5,984262	5,080566
C	-2,410801	-5,244449	6,009347
C	-1,758383	-5,659942	3,720741
C	-3,258449	-4,235141	5,581079
C	-2,602243	-4,638772	3,288999
C	-3,381482	-3,924342	4,212503
C	-2,126234	7,41716	2,578605
C	-3,1322	7,695564	1,63848
C	-1,960411	6,098084	3,02157
C	-3,968588	6,686825	1,187383
C	-2,791844	5,0803	2,558843
C	-3,823157	5,364519	1,65078
C	-4,371744	-2,919223	3,766887
C	-5,61151	-2,800501	4,412663
C	-4,14509	-2,077887	2,665069
C	-6,559169	-1,899253	3,943197
C	-6,262881	-1,120683	2,830458
C	-4,800703	4,328768	1,249998
C	-4,439554	2,992348	1,011757
C	-6,162944	4,648516	1,133347
C	-7,089768	3,664278	0,813319
C	-6,64673	2,359805	0,624541
C	-5,389696	-1,569705	-3,578175
C	-4,871775	-1,02394	-2,395018
C	-6,709902	-2,045525	-3,526942
C	-7,447403	-1,939041	-2,353644
C	-6,857583	-1,362188	-1,232824
N	-5,342353	2,036493	0,726036
N	-5,065779	-1,206448	2,208487

N	-5,583361	-0,924001	-1,260522
C	-7,599441	-1,21152	0,079966
C	-7,265075	-0,138347	2,265241
C	-7,597994	1,237269	0,26525
N	-7,116122	-0,035335	0,810361
Zn	-4,811404	-0,016924	0,484213
H	-0,724766	-4,570864	-9,973024
H	0,02771	-3,698439	-11,336387
H	0,274236	11,107095	1,135366
H	1,287831	11,804848	2,428632
H	1,290921	-7,116815	8,528732
H	2,143656	-8,647407	8,187336
H	0,17105	-4,217747	-7,928926
H	1,871557	-5,304678	7,296748
H	0,867961	9,070659	0,318013
H	1,133303	-3,948673	-5,720738
H	2,169897	-1,124517	-8,77876
H	2,687775	-2,848731	-4,153319
H	3,720433	0,00904	-7,211446
H	1,464883	6,918088	-0,624767
H	3,487626	8,252666	2,914963
H	2,884349	4,902346	-0,698033
H	4,916695	6,228987	2,86585
H	3,968508	-7,109934	4,724116
H	2,442144	-3,319388	6,029026
H	5,209248	-5,985023	2,89526
H	3,678565	-2,180573	4,222721
H	6,680405	5,385934	1,627204
H	3,492036	2,792067	0,298472
H	8,233738	3,464245	1,357718
H	3,821249	-1,892064	1,758055
H	7,088371	-4,5603	2,64988
H	8,413044	-3,378819	0,906622
H	3,190709	-0,811602	-2,833638
H	5,808748	-0,471746	-6,23983
H	7,543909	0,437569	-4,709651
H	8,326762	0,635907	-2,378182
H	7,176856	1,865138	-1,854607
H	7,593076	0,315452	1,342154
H	8,635241	1,295242	0,311426
H	8,65838	-1,3241	-0,465213
H	7,362317	-1,701907	-1,599333
H	-1,443069	-1,71467	-10,850929
H	-2,325366	-3,187527	-11,338737
H	0,548283	-9,075334	6,28085
H	-0,211288	-9,039495	7,895437
H	0,050127	10,386867	4,112725
H	-0,949691	11,613469	3,287535
H	-2,003494	-0,786677	-8,868194

H	-0,564482	8,208571	3,900913
H	-0,295203	-7,665653	4,726925
H	-4,091205	-3,643877	-7,54041
H	-2,531474	0,2754	-6,758729
H	-5,300797	-3,597911	-5,375488
H	-3,735152	0,343566	-4,605637
H	-2,31761	-5,488162	7,062345
H	-1,180824	-6,22303	2,991574
H	-3,824096	-3,669354	6,316353
H	-2,683301	-4,423255	2,227463
H	-3,243506	8,715297	1,285424
H	-1,183446	5,869	3,746901
H	-4,738541	6,920382	0,457024
H	-2,665264	4,070207	2,93783
H	-5,848656	-3,443649	5,254128
H	-3,207826	-2,08477	2,113935
H	-7,53179	-1,81782	4,418828
H	-3,402306	2,667571	1,021833
H	-6,496977	5,662649	1,328328
H	-8,146752	3,89961	0,733193
H	-3,853404	-0,652632	-2,33948
H	-7,167667	-2,461634	-4,418986
H	-8,477399	-2,280109	-2,312246
H	-8,684725	-1,177548	-0,097208
H	-7,408829	-2,094045	0,702419
H	-7,078651	0,855747	2,689052
H	-8,285243	-0,423395	2,562918
H	-8,617671	1,476214	0,602981
H	-7,641363	1,137615	-0,826288
C	0,602853	-0,379995	0,201018
H	0,576514	-1,368065	-0,267239
C	-0,633178	0,444285	-0,188788
H	-0,638021	0,576872	-1,286041
C	1,930302	0,302969	-0,134633
O	2,970725	-0,47549	-0,10291
O	1,978609	1,518538	-0,396392
C	-1,953618	-0,270984	0,168932
O	-3,01189	0,455369	-0,03383
O	-1,954059	-1,435498	0,579924
O	-0,641729	1,710941	0,453408
H	0,217894	2,103829	0,200404
H	0,584347	-0,560943	1,283441

11.5 Mal@1 entry 16

Symbol	X	Y	Z
N	-2,267442	-9,84064	1,389274
C	4,58713	4,584021	6,656634
C	1,88057	3,439835	-7,918906
C	-2,007299	-8,938408	0,527737
C	5,020689	3,66325	5,5892
C	4,41369	2,40825	5,448619
C	6,029482	4,035541	4,685558
C	4,797432	1,542633	4,427007
H	3,637488	2,106131	6,147569
C	6,423452	3,168695	3,678143
H	6,487148	5,013071	4,794257
C	5,813821	1,907938	3,531542
H	4,331398	0,564235	4,347801
H	7,195404	3,478161	2,978714
C	-0,793506	-8,104974	0,62348
C	0,165254	-8,345148	1,621869
C	-0,572484	-7,065029	-0,289584
C	1,326505	-7,590413	1,675253
H	-0,01967	-9,139398	2,337339
C	0,589273	-6,297923	-0,231189
H	-1,308097	-6,866445	-1,065319
C	1,567608	-6,565401	0,739432
H	2,053964	-7,789343	2,457602
H	0,754453	-5,517924	-0,969092
C	2,427397	2,389049	-7,039318
C	2,715745	1,11127	-7,546672
C	2,671965	2,649568	-5,684318
C	3,266057	0,139459	-6,727001
H	2,506569	0,909702	-8,591848
C	3,218689	1,671013	-4,856212
H	2,449616	3,63312	-5,277682
C	3,543744	0,406806	-5,371734
H	3,473103	-0,845454	-7,136375
H	3,430408	1,907333	-3,817236
C	2,86677	-5,856925	0,731185
C	4,056029	-6,543898	1,018848
C	2,984087	-4,501438	0,382963
C	5,27975	-5,894474	0,913806
H	4,024608	-7,598261	1,274359
H	2,118677	-3,884851	0,155724
C	5,307504	-4,559647	0,526243
H	6,208422	-6,425908	1,098226
C	4,241643	-0,60706	-4,551099
C	3,9872	-0,790869	-3,182631
C	5,241676	-1,412797	-5,119854

H	3,215531	-0,223355	-2,669993
C	5,94125	-2,3225	-4,337559
H	5,497685	-1,294296	-6,167705
C	5,639931	-2,421177	-2,983714
H	6,731227	-2,93191	-4,76577
C	6,274337	0,982855	2,473571
C	5,380563	0,201785	1,72609
C	7,63913	0,843567	2,175983
H	4,306614	0,277747	1,859716
C	8,050401	-0,042052	1,187248
H	8,374979	1,409106	2,739253
C	7,091595	-0,783557	0,50337
H	9,103873	-0,167205	0,955621
H	1,728424	4,427839	-7,456228
H	-2,665118	-8,73607	-0,332339
N	4,670473	-1,667078	-2,424929
N	4,170595	-3,871946	0,28304
N	5,778271	-0,661573	0,776235
N	6,48314	-2,815028	-0,720802
Zn	4,394006	-1,863074	-0,348865
H	3,866785	4,176522	7,383443
N	-5,028165	-8,822005	1,969486
C	1,988085	6,706692	5,518514
C	-4,574031	-7,796998	2,574615
C	-1,596152	3,084711	-8,34012
C	0,791272	5,948836	5,103582
C	0,527329	5,71835	3,746654
C	-0,112107	5,459347	6,061697
C	-0,627638	5,0479	3,34743
H	1,220269	6,087523	2,994515
C	-1,26329	4,79721	5,666925
H	0,107204	5,624081	7,111251
C	-1,552521	4,59738	4,302404
H	-0,83179	4,918176	2,288168
H	-1,945564	4,421159	6,42441
C	-2,292041	3,192297	-7,043545
C	-2,650053	4,44828	-6,526374
C	-2,59401	2,042614	-6,301652
C	-3,308019	4,54449	-5,310789
H	-2,400325	5,334325	-7,100214
C	-3,245114	2,138219	-5,07357
H	-2,325533	1,064659	-6,693491
C	-3,621658	3,390881	-4,566009
H	-3,564916	5,525247	-4,919766
H	-3,498425	1,23245	-4,529223
C	-4,998526	-6,432939	2,206095
C	-6,023886	-6,227567	1,268084
C	-4,374887	-5,318792	2,783165
C	-6,424362	-4,942487	0,9361

H	-6,493106	-7,094901	0,815919
C	-4,765407	-4,02684	2,438887
H	-3,583666	-5,465463	3,514357
C	-5,804821	-3,820396	1,519139
H	-7,213289	-4,801767	0,202056
H	-4,289706	-3,176023	2,919097
C	-4,383811	3,516349	-3,305384
C	-5,434384	4,441023	-3,191125
C	-4,117314	2,724316	-2,179099
C	-6,156728	4,535394	-2,008843
H	-5,700035	5,062448	-4,040418
H	-3,295711	2,016137	-2,175733
C	-5,830195	3,700173	-0,945189
H	-6,977876	5,23935	-1,913959
C	-6,290015	-2,458236	1,208175
C	-5,416277	-1,37342	1,039001
C	-7,663151	-2,188949	1,095483
H	-4,33927	-1,498371	1,065061
C	-8,101785	-0,892323	0,855745
H	-8,383443	-2,989422	1,232974
C	-7,161787	0,125683	0,728856
H	-9,161879	-0,667106	0,788459
C	-2,851886	4,013508	3,90232
C	-3,004718	3,186493	2,777601
C	-4,017172	4,320156	4,623722
H	-2,156883	2,887996	2,167328
C	-5,250784	3,844903	4,198387
H	-3,960187	4,972769	5,488827
C	-5,314905	3,059471	3,052916
H	-6,160373	4,10638	4,730419
H	-3,839878	-7,869883	3,392282
H	-1,413681	2,063195	-8,708826
N	-5,839396	-0,117478	0,81256
N	-4,824316	2,804803	-1,038034
N	-4,201072	2,724639	2,366718
N	-6,581483	2,480621	1,043329
Zn	-4,49357	1,563871	0,628179
H	2,626804	7,086723	4,705628
N	1,606199	3,208104	-9,141396
N	-1,221952	4,120735	-8,979893
N	5,012175	5,783519	6,714865
N	2,250752	6,912142	6,748052
C	3,460632	7,63135	7,095542
H	3,933231	8,124652	6,231725
H	3,202447	8,39577	7,837658
C	4,473823	6,658267	7,738083
H	3,975485	6,11271	8,554608
H	5,296429	7,246544	8,159831
C	-3,499682	-10,593504	1,258837

H	-3,963704	-10,494342	0,265056
H	-3,275654	-11,651439	1,436715
C	-4,508505	-10,126251	2,332581
H	-4,011818	-10,134908	3,315195
H	-5,34245	-10,836465	2,354338
C	-0,467729	3,944781	-10,20533
H	-0,543928	2,926355	-10,617017
H	-0,845923	4,654299	-10,949826
C	1,019589	4,267378	-9,937996
H	1,092333	5,257745	-9,461702
H	1,540756	4,302686	-10,901608
C	6,374404	-3,381087	-2,069725
H	7,355153	-3,637236	-2,497144
H	5,806856	-4,316263	-1,996873
C	7,465393	-1,733197	-0,615318
H	7,462354	-1,183454	-1,564376
H	8,488704	-2,111884	-0,472711
C	6,612041	-3,816293	0,342325
H	6,866407	-3,28482	1,267467
H	7,429114	-4,527983	0,15019
C	-6,557514	3,787702	0,380482
H	-7,566535	4,200627	0,235198
H	-6,021949	4,488018	1,032196
C	-6,634666	2,556509	2,507783
H	-7,457912	3,193432	2,864247
H	-6,829097	1,545771	2,885946
C	-7,566536	1,55984	0,467843
H	-8,582557	1,747693	0,846264
H	-7,594415	1,737123	-0,614431
C	-0,766542	-0,353006	-0,081215
H	-0,976799	-1,181654	0,602552
C	0,683658	0,124802	0,06604
H	0,846133	0,478198	1,09831
C	-1,8042	0,747487	0,142821
O	-3,011333	0,328011	0,366053
O	-1,496157	1,95447	0,086516
C	1,704162	-1,003437	-0,1931
O	2,924675	-0,691041	0,127933
O	1,356124	-2,08516	-0,679344
O	0,989768	1,161349	-0,859595
H	0,296022	1,832402	-0,695103
H	-0,905032	-0,755917	-1,091963

11.6 Mal@1 entry 11

Symbol	X	Y	Z
N	2,411419	-4,647485	8,653631
C	7,048253	-1,050006	-5,580307
C	-8,839265	0,864043	-2,960421
C	1,363145	-4,324502	8,005798
C	5,753349	-1,694771	-5,287847
C	5,392398	-2,002555	-3,969137
C	4,863455	-2,016443	-6,326174
C	4,187976	-2,645672	-3,689631
H	6,070565	-1,756151	-3,155755
C	3,667701	-2,660044	-6,051191
H	5,140001	-1,758882	-7,343035
C	3,316464	-3,003038	-4,730482
H	3,948152	-2,911123	-2,663636
H	2,990002	-2,892263	-6,868194
C	1,156302	-4,758093	6,611365
C	2,062086	-5,63421	5,990845
C	0,067385	-4,273886	5,874622
C	1,872841	-6,020773	4,673699
H	2,90985	-5,993713	6,564277
C	-0,117486	-4,651617	4,546812
H	-0,642672	-3,599018	6,346065
C	0,779211	-5,536889	3,929758
H	2,593033	-6,685421	4,204262
H	-0,983381	-4,286175	4,001431
C	-7,894992	-0,221571	-2,633265
C	-8,288641	-1,291005	-1,811838
C	-6,58832	-0,20173	-3,139044
C	-7,408296	-2,325513	-1,537378
H	-9,296005	-1,290672	-1,409232
C	-5,698189	-1,235415	-2,854473
H	-6,271917	0,620947	-3,775653
C	-6,102249	-2,322597	-2,065022
H	-7,728846	-3,143088	-0,89728
H	-4,702619	-1,221654	-3,289949
C	0,568023	-6,002534	2,543084
C	0,809601	-7,338564	2,185733
C	0,104759	-5,152989	1,527854
C	0,572282	-7,766695	0,886102
H	1,149619	-8,04478	2,936532
H	-0,068782	-4,100196	1,721446
C	0,092937	-6,857178	-0,051159
H	0,737468	-8,801738	0,602871
C	-5,223364	-3,495636	-1,864337
C	-3,842262	-3,388615	-1,636837
C	-5,753032	-4,793905	-1,941455

H	-3,359099	-2,424595	-1,515629
C	-4,91735	-5,896645	-1,82851
H	-6,812485	-4,932946	-2,13182
C	-3,554243	-5,693115	-1,640252
H	-5,311549	-6,904932	-1,911715
C	2,100139	-3,80539	-4,474545
C	1,31757	-3,654046	-3,318324
C	1,70998	-4,809518	-5,375008
H	1,542547	-2,901224	-2,569263
C	0,623002	-5,624044	-5,086248
H	2,2912	-4,984839	-6,274457
C	-0,074445	-5,427444	-3,899786
H	0,33767	-6,428385	-5,757375
H	-8,474035	1,624578	-3,668158
H	0,573128	-3,690216	8,437195
N	-3,029281	-4,455925	-1,536133
N	-0,133486	-5,566249	0,271354
N	0,260607	-4,440696	-3,040232
N	-1,271314	-6,463405	-2,040677
Zn	-0,885686	-4,322261	-1,264111
H	7,69771	-0,853293	-4,71275
N	3,057177	-1,811275	9,196944
C	6,625819	2,393944	-6,181548
C	3,434125	-1,558947	8,006025
C	-8,743482	3,306353	-0,572383
C	6,023809	3,039636	-5,000241
C	4,632985	3,179779	-4,903479
C	6,828222	3,501523	-3,945571
C	4,052645	3,765252	-3,78055
H	4,000835	2,832401	-5,717137
C	6,252997	4,097172	-2,833936
H	7,903406	3,379078	-4,022278
C	4,855722	4,240481	-2,732827
H	2,973973	3,888059	-3,73501
H	6,889274	4,436572	-2,021009
C	-7,382974	3,868759	-0,483772
C	-6,98043	4,912863	-1,332984
C	-6,465924	3,359888	0,445565
C	-5,706312	5,448425	-1,230011
H	-7,689716	5,290476	-2,06185
C	-5,181033	3,889398	0,54326
H	-6,765585	2,551384	1,107972
C	-4,788703	4,954554	-0,282336
H	-5,410403	6,250704	-1,90057
H	-4,49629	3,501306	1,29221
C	2,838911	-0,458404	7,224033
C	1,858043	0,37238	7,790582
C	3,250146	-0,216356	5,906409
C	1,344503	1,440221	7,072988

H	1,526585	0,166679	8,802837
C	2,730129	0,854089	5,180812
H	4,002283	-0,85713	5,45262
C	1,788658	1,715549	5,764191
H	0,583831	2,068763	7,527764
H	3,093542	1,046076	4,175332
C	-3,472784	5,608293	-0,112003
C	-3,349414	7,002765	-0,212215
C	-2,310075	4,889119	0,206895
C	-2,129684	7,615196	0,046071
H	-4,221046	7,608136	-0,439869
H	-2,310287	3,806952	0,295666
C	-1,037719	6,827461	0,393825
H	-2,032013	8,696031	0,012445
C	1,348394	2,955027	5,08575
C	1,134991	3,047119	3,700387
C	1,182035	4,1337	5,830688
H	1,195421	2,169086	3,06343
C	0,846949	5,32219	5,197555
H	1,357384	4,123492	6,901431
C	0,692935	5,331305	3,816002
H	0,734858	6,241968	5,76337
C	4,265784	4,922399	-1,560304
C	3,080305	4,478206	-0,954738
C	4,871056	6,062561	-1,008984
H	2,563334	3,587057	-1,294263
C	4,28786	6,704223	0,076496
H	5,778162	6,46094	-1,452779
C	3,103479	6,197531	0,603042
H	4,735799	7,597356	0,501739
H	4,221644	-2,140112	7,50062
H	-8,997663	2,538523	0,17527
N	0,833016	4,205341	3,083554
N	-1,12679	5,482009	0,449711
N	2,513101	5,099847	0,093365
N	0,981527	6,603009	1,749136
Zn	0,640206	4,428944	0,997786
H	5,932444	2,140961	-6,999155
C	-0,10394	0,574141	-0,457661
H	-1,019056	0,468828	-1,054047
C	-0,013433	-0,593129	0,54859
H	-0,903539	-0,573004	1,184323
C	-0,160617	1,917324	0,275045
O	0,755941	2,781001	-0,043928
O	-1,046608	2,136176	1,114563
C	0,070282	-1,946882	-0,18317
O	1,194916	-2,449001	-0,354251
O	-1,039422	-2,485473	-0,574509
O	1,125345	-0,448096	1,382844

H	1,814236	-0,990404	0,951817
N	-10,003761	0,911825	-2,445467
N	-9,565299	3,683905	-1,470119
N	7,386181	-0,745631	-6,770266
N	7,870816	2,129283	-6,235826
C	8,371585	1,404672	-7,38732
H	7,675482	1,4158	-8,240583
H	9,318425	1,858652	-7,700516
C	8,646306	-0,061534	-6,984221
H	9,30036	-0,074866	-6,098303
H	9,168394	-0,55273	-7,813498
C	2,604377	-4,084778	9,9754
H	1,676075	-3,689602	10,416371
H	3,003258	-4,864518	10,633581
C	3,643528	-2,943054	9,887258
H	4,55321	-3,323723	9,396944
H	3,898296	-2,632295	10,906941
C	-10,85261	3,022661	-1,550523
H	-11,109003	2,462949	-0,637492
H	-11,623579	3,781268	-1,725781
C	-10,848979	2,049309	-2,752524
H	-10,525865	2,59605	-3,651892
H	-11,872099	1,689628	-2,907997
C	-2,589005	-6,852364	-1,531494
H	-2,991828	-7,735257	-2,050072
H	-2,471621	-7,131149	-0,47729
C	-1,225312	-6,324848	-3,500255
H	-2,173288	-5,87837	-3,823026
H	-1,141598	-7,296232	-4,010003
C	-0,17626	-7,263749	-1,48435
H	0,72025	-7,074893	-2,086411
H	-0,376634	-8,343741	-1,543275
C	0,298298	7,437517	0,75593
H	0,15927	8,472492	1,1036
H	0,938492	7,484683	-0,133391
C	2,427704	6,832177	1,798494
H	2,677322	7,902243	1,859078
H	2,809868	6,367207	2,715303
C	0,348912	6,604311	3,071489
H	0,632532	7,482252	3,670637
H	-0,73635	6,656014	2,927635
H	0,758138	0,551592	-1,130758

12 References

(1) Gaussian 16, Revision C.01, M. J. Frisch, G. W. Trucks, H. B. Schlegel, G. E. Scuseria, M. A. Robb, J. R. Cheeseman, G. Scalmani, V. Barone, G. A. Petersson, H. Nakatsuji, X. Li, M. Caricato, A. V. Marenich, J. Bloino, B. G. Janesko, R. Gomperts, B. Mennucci, H. P. Hratchian, J. V. Ortiz, A. F. Izmaylov, J. L. Sonnenberg, D. Williams-Young, F. Ding, F. Lipparini, F. Egidi, J. Goings, B. Peng, A.Petrone, T. Henderson, D. Ranasinghe, V. G. Zakrzewski, J. Gao, N. Rega, G. Zheng, W. Liang, M. Hada, M. Ehara, K. Toyota, R. Fukuda, J. Hasegawa, M. Ishida, T. Nakajima, Y. Honda, O. Kitao, H. Nakai, T. Vreven, K. Throssell, J. A. Montgomery Jr., J. E. Peralta, F. Ogliaro, M. J. Bearpark, J. J. Heyd, E. N. Brothers, K. N. Kudin, V. N. Staroverov, T. A. Keith, R. Kobayashi, J. Normand, K. Raghavachari, A. P. Rendell, J. C. Burant, S. S. Iyengar, J. Tomasi, M. Cossi, J. M. Millam, M. Klene, C. Adamo, R. Cammi, J. W. Ochterski, R. L. Martin, K. Morokuma, O. Farkas, J. B. Foresman, D. J. Fox, Gaussian, Inc., Wallingford CT, 2016.

(2) GaussView, Version 6, Dennington, R., Keith, T. A., Millam, J. M., Semichem Inc., Shawnee Mission, KS, 2016.

(3) Bruhn, T.; Schaumlöffel, A. N. U.; Hemberger, Y.; Bringmann, G. SpecDis : Quantifying the Comparison of Calculated and Experimental Electronic Circular Dichroism Spectra. **2013**, *25* (4), 243–249. https://doi.org/10.1002/chir.

(4) Gaussian. Gaussian 16, Revision C.01, Frisch, M. J., Trucks,.

(5) Bruhn, T.; Schaumlöffel, A.; Hemberger, Y.; Bringmann, G. SpecDis: Quantifying the Comparison of Calculated and Experimental Electronic Circular Dichroism Spectra. *Chirality* **2013**, *25* (4), 243–249. https://doi.org/10.1002/chir.22138.

(6) Debie, E.; De Gussem, E.; Dukor, R. K.; Herrebout, W.; Nafie, L. A.; Bultinck, P. A Confidence Level Algorithm for the Determination of Absolute Configuration Using Vibrational Circular Dichroism or Raman Optical Activity. *ChemPhysChem* **2011**, *12* (8), 1542–1549. https://doi.org/10.1002/cphc.201100050.

(7) Evans, R.; Deng, Z.; Rogerson, A. K.; McLachlan, A. S.; Richards, J. J.; Nilsson, M.; Morris, G. A. Quantitative Interpretation of Diffusion-Ordered NMR Spectra: Can We Rationalize Small Molecule Diffusion Coefficients? *Angew. Chemie - Int. Ed.* **2013**, *52* (11), 3199–3202. https://doi.org/10.1002/anie.201207403.

(8) Macchioni, A.; Ciancaleoni, G.; Zuccaccia, C.; Zuccaccia, D. Determining Accurate Molecular Sizes in Solution through NMR Diffusion Spectroscopy. *Chem. Soc. Rev.* **2008**, *37* (3), 479–489. https://doi.org/10.1039/b615067p.
